# The First Review on Nano‐Agricultural Applications of MXene and MBene‐Based Materials for Plant‐Immunoengineering, Controlled Protection, and Inducing Biostimulation Mechanisms

**DOI:** 10.1002/adma.202510350

**Published:** 2025-10-16

**Authors:** Alireza Rafieerad, Ahmad Amiri, Maik Böhmer, Soofia Khanahmadi

**Affiliations:** ^1^ Institute for Molecular Biosciences Johann Wolfgang Goethe Universität 60438 Frankfurt am Main Germany; ^2^ Institute for Biology and Biotechnology of Plants University of Münster Schlossplatz 8 48143 Münster Germany; ^3^ Regenerative Medicine Program Institute of Cardiovascular Sciences St. Boniface Hospital Research Centre Department of Physiology and Pathophysiology Rady Faculty of Health Sciences University of Manitoba Winnipeg Manitoba R2H 2A6 Canada; ^4^ Russell School of Chemical Engineering University of Tulsa Tulsa OK 74104 USA; ^5^ Department of Mechanical Engineering The University of Tulsa Tulsa OK 74104 USA

**Keywords:** biostimulation, biotic/abiotic stresses, defense/growth enhancement mechanisms, MXenes/MBenes, nano‐agriculture, plant‐immunoengineering, sustained release of agrochemicals

## Abstract

Producing quality food crops with a focus on climate and environmental improvement policies has become central to modern farming and sustainability strategies. However, the rising world population and food demand have region‐dependently pushed these boundaries to the overuse of agrochemical inputs. These include plant antimicrobials, pesticides, and soil fertilizers, applied to boost crop yields, reaching a critical juncture. The reliance on agrochemicals has been proven effective in priming, plant growth, and enhancing defense/resistance to biotic stressors, such as phytopathogens and invasive organisms, as well as abiotic pressures, including heat, drought, salinity, and light stress, by increasing nutrient absorption and innate immunity or adaptive stress resistance. However, increasing concerns about the safety, cost, and environmental impact of agrochemicals have intensified the necessity for applying sustainable precision technologies. Nano‐agriculture has introduced emerging possibilities for utilizing low‐dimensional biomaterials for plant protection/stimulation applications, once these technologies are proven safe. Among them, carbon‐based MXenes and derivatives (MBenes) show potential due to their high surface‐to‐volume area, biocompatibility at controlled doses, and tunable physicochemical/biological properties. These unique specifications support targeted delivery and sustained release, while also enhancing plant growth and stress tolerance. This comprehensive review covers their effect on seed germination, seedling maturation, plant‐immunoengineering, priming, eliciting, stomatal closure, antimicrobial actions, and gene or phytohormone regulation. It also discusses their role as sustainable carriers for the delivery and release of agrochemicals and plant protection by nano‐design, aiming to reduce agrochemical consumption. Lastly, we discuss the current environmental regulations for nanomaterials and recommend rational outlooks for future work.

## Graphical abstract

MXene and MBene nanomaterials show significant potential in addressing critical challenges in biomedicine, applied biology, agriculture, and the environment. From a nano‐agricultural perspective, this relatively young field has witnessed emerging advances towards applications for plant‐immunoengineering, biostimulation, and controlled delivery/sustained release of agrochemicals. These strategies encompass the design, fabrication, surface modification, and post‐functionalization of MXene/MBene‐based biomaterials, enabling the enhancement of plant defense mechanisms against biotic/abiotic stresses, as well as the promotion of growth for high‐yield production to reduce the reliance on agrochemical inputs and alleviate their risks to human health and the environment. This innovative review presents comprehensive discussions on the proposed mechanisms, modes of action, biocompatibility, and future outlooks.

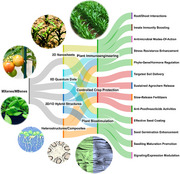

## 1 Introduction

The necessity of treating agricultural plants and crops with antimicrobials, anti‐pests/insecticides, and ultimately soil‐fertilizer supplementation is referred to as the three primary subjects. First, they are rich sources of natural nutrients and, as such, are constantly attacked by invading organisms, including phytopathogens, pests, insects, nematodes, etc.^[^ ^1^, ^2^, ^3^, ^4^, ^5^, ^6^ ^]^ Second, they are sessile and cannot move or migrate to protect themselves against harsh environmental conditions, such as severe heat, drought, salinity, and light stresses such as excessive sun irradiation or growth at long dark cycles. Even though they naturally have sophisticated immune responses and different structural/chemical defense mechanisms to tackle these biotic and abiotic stresses to their best capacity and capability, this reliance has been shown to be insufficient in warding off the invaders and destroying them or resisting abiotic stressors.^[^ ^7^, ^8^, ^9^, ^10^, ^11^, ^12^ ^]^ These sustained stresses can ultimately cause substantial losses in the yield and quality of agricultural plants, limiting their production to be lower than 30–70% of their genetic potential in a region or climate‐dependent manner.^[^ ^13^ ^]^ To control or alleviate the occurrence of extensive plant root/shoot damage, agricultural management strategies have been developed and employed at different scales around the globe. The third challenge refers to the insufficient capability of plants to obtain all their essential nutrients in a usable form through root/shoot absorption/uptake mechanisms, ecosystem cycles, and/or sustainable interactions with beneficial micro/organisms. Even though plants innately operate these interactional mechanisms for survival, photosynthesis, and growth, their capacity to sustainably acquire these requirements is limited, and fertilization is required to compensate and produce high‐yielding/quality crops.^[^ ^12^, ^13^, ^14^, ^15^, ^16^ ^]^

Due to the increasing trend of the world's population, food demands, and universal economic competition, the agriculture and farming industry has remained among the top technological sectors.^[^ ^17^, ^18^, ^19^, ^20^ ^]^ Thereby, extensive ongoing research and development investigations have been conducted to increase and improve the production and technology of agricultural foods. The sectors of modern agriculture have proven effective in large‐scale production of high‐yield crops with variable levels of success in controlling food demands. Despite these management strategies, the increasing concerns regarding the risks of large‐scale implantation of agrochemicals to human health, farmworker safety, climate change, and other environmental impacts have pressurized modern agriculture.^[^ ^21^, ^22^, ^23^, ^24^, ^25^, ^26^, ^27^ ^]^ In this context, universal attempts to improve the biocompatibility and safety of agrochemical‐based technologies both in production and implantation aspects, as well as longer‐term plans to replace agrochemical products with more sustainable and eco‐friendly approaches, have already established emerging paradigms aimed at improving climate change and reducing safety risks to human health. These global consideration policies have been focused on the forefront of the agricultural fields towards designing, developing, and applying newer and greener‐generation technologies.

In parallel, over recent years, despite all these management considerations, global farming has faced critical challenges in producing quality foods with balanced sustainability and safety factors. Indeed, the established plans for high‐yield, greener agriculture have not been thoroughly matched with the currently existing limitations of providing higher amounts of crops that could satisfy the tremendously increased food demands in the future. The world's population is expected to reach over 9.5 billion by 2050. It is also predicted to further rise to ≈11 billion by the end of the current century.^[^ ^28^ ^]^ This ongoing statistic, coupled with the existing challenges on sustainable production of high‐quality crops, while avoiding the reliance on excessive consumption of agrochemicals, has significantly affected natural resources and already placed double pressure on today's agricultural sectors and farmers universally relative to the management policies of each farming region/land. This contradictory statement of farming more crops while considering health and environmental constraints is already on the verge of its tipping capacity, which needs to be urgently modified with combined multimodal strategies or replaced by more eco‐economically friendly alternatives. In this regard, one of the most critical aspects to be addressed is the increased carbon dioxide and other gases in the atmosphere, representing a crucial threat to climate change, which directly and indirectly affects human and crop security.^[^ ^19^, ^22^, ^23^, ^24^, ^25^, ^26^, ^27^ ^]^

According to the available reports, these alterations in the planet's landscape and atmosphere continue to adversely influence ecological interactions such as carbon cycle, climate changes, and increased earth's temperature, further highlighting the necessity of prompt actions for developing or implementing greener plant antimicrobials, growth promotors, defense boosters, and drug delivery agents for multimodal treatments, aiming to reduce the consumption of agrochemical products.^[^ ^19^, ^20^, ^21^, ^22^, ^23^, ^24^, ^25^, ^26^, ^27^, ^28^ ^]^ These emerging factors hold a significant burden to the global agricultural strategies and food chains in terms of both industrial processing and market competition, sustainability, and health aspects. As such, the agricultural sectors have strategized region‐dependent management to balance economic profits and environmental considerations. The esteemed sustainable agriculture is considered based on regional management strategies to support the production of sufficient food for the growing populations and provide economic livelihood profits to farmers while maintaining the quality of products and improving climate change. In particular, these policy considerations have been recently reported by “The Organization for Economic Cooperation and Development” to simultaneously adapt to emerging climate changes and support farming practices within the framework of sustainable agriculture.^[^ ^29^, ^30^, ^31^ ^]^ This development strategy is expected to improve the variety of corresponding technologies, including the applications of advanced tools, innovative methodologies, and precise agricultural productions.

More recently, it has been proposed that such scenarios might be further promoted by applying nanotechnology, paving the way forward for further practices of bioactive nanomaterials in future farming and their real‐world applications in agricultural inputs.^[^ ^19^, ^32^, ^33^, ^34^, ^35^, ^36^, ^37^, ^38^, ^39^, ^40^, ^41^, ^42^ ^]^ In this regard, the European Commission commented in 2020 on the “Farm to Fork Strategy” within the wider Green Deal management policy. In this particular document, Europe has aimed for significant reductions in the consumption of agrochemical pesticides (50%), nutrient losses (at least 50%), and chemical fertilization (at least 20%) by the end of 2030.^[^ ^43^, ^44^ ^]^ This management strategy also covers the plan to increase the organic cultivation of the corresponding agricultural lands to 25%. Given the profound impact of climate change on agriculture and due to the importance of related policy considerations, we dedicated a particular sub‐section before the conclusion part to further discuss the current regulatory frameworks in global agricultural managements, including the “European Green Deal” policies, “Farm to Fork Strategy”, “EFSA” guidance on nanomaterial safety in food and agriculture, and other existing regulations and constrains within real‐world implementation and emerging considerations for future nano‐technological opportunities in agriculture.

In this direction, one of the notable steps made in enhancing sustainability was the policy of developing highly biocompatible plant biostimulants as potential alternatives to agrochemicals. This strategy is expected to enhance the crops’ growth, quality, and yield more eco‐economically, anticipating safely improving plants’ physiological, metabolic, and stress defense response without causing significant adverse effects. This concern includes the health‐related quality of plants and their surrounding environments, including soil, underground water, and beneficial microbiomes and organisms. Indeed, an ideal biostimulant must be sustainable, biocompatible, and also safe to humans and ecosystems. These compounds could be extracts/substances/materials from natural or nature‐inspired resources, such as bio‐wastes and biopolymers with high biodegradability due to their carbonaceous compositions. Despite the potential of natural biostimulants, their applications are limited and suffer from long‐term effects, multifunctionality, and multiple treatments.

Nanotechnology‐enabled strategies have shown promise in addressing these challenges, either alone or in combination with current agrochemical applications to fulfill the as‐defined objectives of sustainable agriculture.^[^ ^34^, ^45^, ^46^, ^47^, ^48^, ^49^ ^]^ These approaches involve the direct and/or indirect impacts of nano‐ and quantum‐engineered materials for controlled delivery of pesticides and for plant protection and biostimulations. There have also been unprecedented possibilities to combine these nanosystems with natural plant biostimulants for synergistic strategies, benefiting from lower production costs and decreased risks of nano‐toxicity at higher doses or more prolonged exposures. This rational nano‐strategy may act as a future gold‐standard solution for producing high‐yield and low‐cost crops, while maintaining their quality.

Among these nanomaterial‐enabled technologies, ample attention has been given to advanced carbon‐based materials, particularly bioactive compositions of MXenes and their akin derivatives (MBenes) for various applications in plant protection and biostimulation due to their unique and tunable physicochemical/biological properties. Thus, due to the significance of MXene/MBene‐based nanomaterials in this interdisciplinary field, we have dedicated a standalone background sub‐section in the following part, describing their emergence, synthesis strategies, properties, and potential for agricultural applications.

Given that this emerging field is both timely and essential, in this innovative and comprehensive review, we presented a classified roadmap on the existing literature of MXenes and MBenes and their bioactivity impacts on plant protection and biostimulation in a one‐stop‐read systematic review of the state‐of‐the‐art advancements in reported nano‐agricultural applications of MXene/MBene‐based materials, including nanosheets, quantum dots, hybrid systems, and derived heterostructures in a time‐dependent and novelty fashion. We first discussed the basic principles and strategies for fabricating MXenes/MBenes, including top‐down and bottom‐up approaches, as well as the reported structural and surface modifications related to their property enhancement. We then discussed the entire literature in detail in three main application categories: “controlled delivery/sustained release,” “plant immunoengineering,” and “biostimulation.” Of note, we explored their reported properties under normal and stress conditions in seeds, seedlings, model plants, and crops, discussing the proposed modes of action and biological mechanisms induced by these materials. Lastly, we compared their biocompatibility properties and discussed the challenges toward their practical applications, providing constructive outlooks and rational ideas for future studies in this rapidly evolving field.

## 2 “MXene/MBene Nanomaterials”: Synthesis, Bio‐Properties, and Agricultural Potential

MXenes are the latest‐ and largest‐class of discovered carbon‐based nanomaterials. Since the first report on the fabrication of the first composition of 2D MXene sheets and 0D MXene quantum dots, they have received significant credit due to their unique multifunctional properties.^[^ ^50^, ^51^, ^52^, ^53^, ^54^, ^55^, ^56^ ^]^ MXenes have the formula of M_n+1_X_n_T_x_ (n = 1‐4), where “M” stands for one or more transition metals in the periodic chemical elements, “X” denotes carbon and/or nitrogen, and “T_x_” represents various surface functional groups (e.g., oxygen, nitrogen, hydrogen, fluorine, and/or chlorine‐based terminals. These tunable materials have been reported to possess hydrophilic surface properties.^[^ ^57^, ^58^, ^59^, ^60^, ^61^, ^62^, ^63^, ^64^ ^]^ Further, specific compositions of MXene materials at controlled concentrations have also been reported to offer high levels of biological compatibility with a wide range of bio‐systems such as cells, tissues, organs, mammals, and living plant systems.^[^ ^43^, ^64^, ^65^, ^66^ ^]^ Bioactive MXenes also have the intrinsic capability to be selectively taken up by cells and tissues to effectively stimulate, inhibit, deliver, detect, or monitor specific biological and immunological responses^.^ Moreover, bioactive MXenes have shown potential properties for future applications in the era of biological and environmental sensing. Thereby, the emergence of MXene nanotechnology in agriculture has established a promising field with emerging reports of its applications in plant‐immunoengineering, protection, and biostimulation. The field remains dynamic, with lots of room for advancing its current technology in terms of production optimization, property modification, and new bio‐applications.

More recently, computational predictions and experimental developments of a new derivative family of low‐dimensional materials akin to MXenes, known as transition‐metal borides (MBenes), have been reported.^[^ ^67^, ^68^, ^69^ ^]^ This emerging class of nanomaterials has general formulas of MB, MB_2,_ M_2_B_2_, and M_3_B_4_, as well as hybrid phases ((M′_2/3_M″_1/3_)_2_B_2_ and M′_4/3_M″_2/3_B_2_), where “M′” represents transition metals in the periodic table of elements such as titanium, chromium, manganese, iron, molybdenum, tungsten, and “M″” can theoretically include scandium, yttrium, zirconium, niobium, and hafnium. Striking similarities in structure and properties of MBenes with MXenes highlight the potential of this class of low‐dimensional materials for bio‐applications, such as antibacterial and anticancer properties, as well as their effects on plant biostimulation.^[^ ^70^, ^71^, ^72^, ^73^ ^]^ To date, in comparison to MXenes, a lower frequency of MBenes has been reported. However, this parallel field is also growing fast in both terms of synthesis and applications. MBenes have been reported to possess high structural and physico‐mechanical properties, highlighting their suitability for applications that require more durability and long‐term stability of bioactive and electroactive‐functional materials. Hence, their biocompatibility for agricultural applications, including plant carriers or enhancers, has been thoroughly discussed and compared with the MXene counterparts. **Table** **1** represents some of these biocompatibility properties across diverse living biosystems.

**Table 1:** The biocompatibility/nanotoxicity of representative MXenes/MBene‐based materials and their working doses and tested time points in different biological systems and plant models.

MXenes/MBenes chemical compositions	Tested working doses	Treatment/exposure durations	In vitro/In vivo/In Planta Models	Biocompatibility or nano‐toxicity inferences	Refs.
2D Ti_3_C_2_T_x_ nanosheets	100 and 500 µg mL^−1^	for 7 days	Human umbilical vein endothelial cells HUVECs	No significant change in the viability of these cells was reported in Ti_3_C_2_T_x_‐treated groups compared to the control.	[74]
0D Ti_3_C_2_T_x_ and Nb_2_CT_x_ MXene quantum dots	0 to 100 µg mL^−1^	for 24 h	HUVEC cells	Ti‐based quantum dots are reported to induce some cytotoxicity effects at a higher dose (100 µg mL^−1^). Nb‐based quantum dots did not cause obvious toxicity.	[75]
2D Ti_3_C_2_T_x_ MXene nanosheets	0 to 100 µg mL^−1^	for 7 days	Human mesenchymal stem cells	Ti_3_C_2_T_x_ sheets with doses over 50 µg mL^−1^ are reported with some cytotoxicity to these cells, but at lower concentrations (<50 µg mL^−1^), significant cytotoxicity is reported. Also, a dose lower than <20 µg mL^−1^ accelerated osteogenic differentiation.	[76]
2D Ti_3_C_2_T_x_ MXene nanosheets	0 to 25 µg mL^−1^	for 24 h	Neural stem cells and derived differentiated secondary cells	These Ti_3_C_2_T_x_ sheets with doses higher than >25 µg mL^−1^ are reported with cytotoxicity. No toxicity is reported at 12.5 µg mL^−1^.	[77]
Mono/oligo/ multi‐layered Ti_3_C_2_T_x_ MXene nanosheets and TiC/Ti_2_AlC/Ti_3_AlC_2_ MAX‐phase bulks	10 to 400 µg mL^−1^	up to 48 h	Human fibroblast cell lines (MSU1.1)	A dose‐manner cytotoxicity is reported in these MAX phases. But the layered MXene nanosheets showed no significant cytotoxicity compared to MAX‐phases and controls.	[78]
2D Nb_2_CT_x_ MXene nanosheets	0 to 200 µg mL^−1^	for 24 h	Murine breast cancer 4T1 and U87 cell lines	No significant cytotoxic effects to these cells at doses up to 200 µg mL^−1^ with laser intervention	[79]
0D Ti_2_N‐based MXene quantum dots	0 to 80 ppm	for 24 h	4T1, U87, and 293T cells	These quantum dots are reported to possess high biocompatibility at doses.	[80]
2D Ti_3_C_2_T_x_ MXene nanosheets	25 to 200 µg mL^−1^	for 96 h after fertilization	Zebrafish embryo model	Ti_3_C_2_T_x_ at doses up to 100 µg mL^−1^ showed no significant signs of teratogenic/neurotoxicity without any adverse effects on neuromuscular activity.	[81]
Ti_3_C_2_T_x_ MXene nanosheets	0 to 200 µg mL^−1^	for 4 h	Vero E6 cells	Showed no obvious toxicity to the cells at tested doses.	[82]
2D Ta_4_C_3_T_x_ MXene‐soybean phospholipid nanosheets	5 to 20 mg kg^−1^	for 30 days	Mouse model (in vivo)	Showed in vitro*/in vivo* biocompatibility and superior photothermal‐conversion performance with the ablation of tumors	[83]
Non‐oxidized Ti_3_C_2_T_x_‐based MXene quantum dots	25 to 200 µg mL^−1^	for 6 h and up to 12 days	Normal retina pigment epithelium (RPE) cells	Showed excellent biocompatibility with normal retinal pigment epithelium cells without any significant damage in C57BL/6 mice eyes	[84]
0D Ti_3_C_2_ MXene‐based quantum dots	10 to 20 µg mL^−1^	for 24 h	RAW264.7 cells	No significant cytotoxicity was reported in these treated cells compared to the control group.	[85]
N‐/P‐functionalized Ti_3_C_2_ MXene‐based quantum dots	0 to 100 µg mL^−1^	for 24 h	THP‐1 monocytes	These quantum dots showed a modest cytotoxicity effect at lower concentrations below 25 µg mL^−1^.	[86]
2D V_2_C MXenzyme	0 to 400 µg mL^−1^	for 24 h	L929 and PC12 cells	Showed no obvious cytotoxicity to these cells at doses up to 200 µ µg mL^−1^.	[87]
0D/1D/2D Ti_3_C_2_T_x_ MXene‐based heterostructures	0 to 100 µg mL^−1^	up to 8 days	*Nicotiana benthamiana / Arabidopsis thaliana* Col‐0 seed/seedling	Showed no significant cytotoxicity to these seeds/seedlings and maturation processes at the tested concentrations	[65]
0D/1D/2D Ti_3_C_2_T_x_ MXene‐based heterostructures	40 and 100 µg mL^−1^	for 48 h	*Nicotiana benthamiana / Arabidopsis thaliana* Col‐0 mature plants	Showed no visible and significant cytotoxicity to these model plants at the tested concentrations	[65]
0D/1D/2D Left‐/right‐handed chiral Ti_3_C_2_T_x_‐based MXene heterostructures	10, 88 and 100 µg mL^−1^	up to 8 days	*Arabidopsis thaliana* Col‐0 mature seed/seedling	Showed no significant cytotoxicity to these model seeds/seedlings at the tested concentrations	[66]
0D/1D/2D Right‐handed chiral Ti_3_C_2_T_x_‐based MXene colloids	100 µg mL^−1^	up to ∼4 weeks (28 days)	*Arabidopsis thaliana* Col‐0 mature plants	Showed no visible and significant cytotoxicity to these plants at the tested concentrations	[66]
2D MoAlB@MBene nanosheets	0 to 1000 mg dm^−3^	4or 48 and 72 h	Higher plants *L. sativum*, *S. alba*, and *S. saccharatum*	No reported significant cytotoxicity to these plants (roots) and the surrounding soil area at tested doses.	[73]

### 2.1 An Overview of Possible Strategies for the Production and Modification of MXenes/MBenes

**Figure 1:**
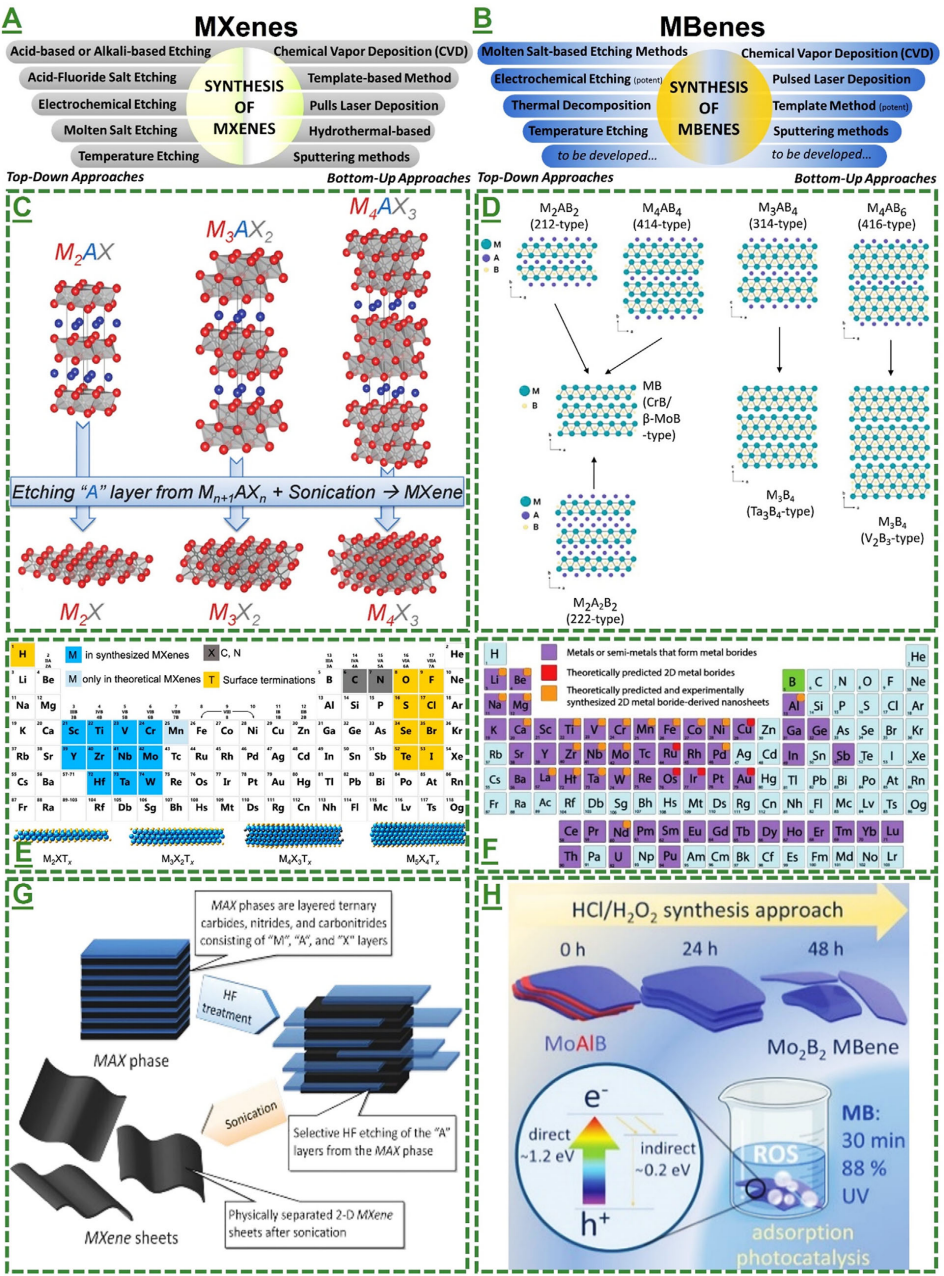
Generally available strategies for fabricating MXenes/MBene‐based nanosheets. A‐D) The schematic representation of top‐down and bottom‐up methods, including approaches for constructing 2D MXene and MBene nanosheets by eliminating “A” layers from their bulky 3D MAX/MAB‐phase precursors using different etchants. Plus, other methods to form layered MXene/MBene materials through physical/chemical reactions, such as chemical vapor deposition or laser/template‐based synthesis. Top‐down methodologies are reported for large‐scale and quality MXenes with relative control over their flake size, surface terminations, intercalations, and interlayer spacing. Bottom‐up approaches also have their advantages over top‐down methods, such as direct deposition of MXene films/layers on defined substrates, more control over their chemical reaction and stoichiometries, higher purity, and crystallization degrees, or probably a more eco‐friendly process due to lower waste/disposal likely with some additional costs and challenges associated with complex equipment. C,D) Represented schematic illustration depicting the chemical structure of the MAX/MAB‐phases and their corresponding MXenes/MBenes. C) Reproduced with permission.^[^ ^53^ ^]^ Copyright 2013, WILEY‐VCH Verlag GmbH & Co. KGaA, Weinheim. D) Reproduced with permission.^[^ ^69^ ^]^ Copyright 2023 Wiley‐VCH GmbH. E,F) The as‐designed periodic table of MAX/MAB‐phases and derived MXenes/MBenes (in theory or by synthesis) illustrating chemical composition possibilities of MXenes/MBenes. The represented elements used to prepare MXenes/MBenes are classified based on color‐coding, along with representative structure. The panels differentiate the molecular structure of MXenes from MBenes, depicting experimental possibilities or theoretical calculation properties of these materials in a periodic fashion. E) Reproduced with permission.^[^ ^88^ ^]^ Copyright 2021, American Chemical Society. F) Reproduced with permission.^[^ ^89^ ^]^ Copyright 2021 American Chemical Society. G) A schematic illustration of a typical synthesis step‐by‐step route for converting representative titanium aluminum carbide MAX phase to titanium carbide‐based MXene sheets by HF‐acid‐based etching/exfoliation method. G) Reproduced with permission.^[^ ^53^ ^]^ Copyright 2013, WILEY‐VCH Verlag GmbH & Co. KGaA, Weinheim. H) Schematic representation of an innovative wet‐chemical etching for synthesis/delamination of a molybdenum boron‐based MBene nanosheet structure from its 3D MAB phase (MoAlB). H) Reproduced with permission.^[^ ^90^ ^]^ Copyright 2021 Wiley‐VCH GmbH. The designed route is dedicated to this specific MBene composition and not as a common acid‐etching of MAX phases or the standard synthesis procedure for all MAB phases. Despite the potential of this method, its feasibility to convert other MAB‐phases to MBenes needs to be tested. However, following their report, several studies have extended the introduced route. This method and microwave/ultrasound‐assisted paths, are among the most promising and scalable reported methods. The panels (C–H) are merged with permission from references, Journals Licenses/Copyright.^[^ ^53^, ^88^, ^89^, ^90^ ^]^

It has been extensively reported that the synthesis process, including experimental parameters and concentrations, plays a key role in defining, designing, and improving the atomic structure and nano/quantum configuration of MXenes/MBenes. Besides, their biocompatibility and bioactivity, either in the form of sheets or quantum dots, are highly sensitive to size, interlayer spacing/lateral thickness, doping, and surface properties. Indeed, most of these factors are reported to be directly or indirectly influenced by the applied synthesis and functionalization parameters, as well as the quality of the used precursors and handling operations. Since the emergence of MXenes/MBenes, the field has remained dynamic for new designs and rational improvements in their synthesis, and recently, with reliance on greener and sustainable production. The possible routes for synthesizing MXenes/MBenes are categorized into two primary approaches, including top‐down and bottom‐up methods. **Figure** **1** represents the schematic illustrations and periodic vacancies of MXenes and MBenes, depicting the method variations, common synthesis processes, and chemical composition possibilities for fabricating 2D MXene/MBene nanosheets. In the particular case of MBenes, despite some similarities with MXenes, their fabrication is to some extent different and needs specific considerations.^[^ ^68^, ^69^, ^70^, ^71^, ^72^, ^73^ ^]^ In the following sections, we have discussed some of these aspects.

#### 2.1.1 Methods for Synthesis of MXene Nanosheets

In particular, top‐down approaches have been widely used to convert 3D MAX‐phase bulks into their planar layered materials. The formed MXene nanosheets, including accordion‐like multi‐, oligo, and mono‐layered, and delaminated nanosheets, have been developed and improved over time by applying new methods alongside hybridization with combinational synthesis strategies. As presented in the adapted figure, acid‐based (e.g., hydrofluoric/hydrochloric acid (HF/HCl), acid‐fluoride salt (sodium/lithium/potassium), and inorganic alkali metal fluoride etching methods are among the most common routes. They act by significantly removing the “A” layers (elements of group 13, 14 of the periodic table) from the structure of their MAX phases. These exfoliations have been expanded by different techniques, including physicochemical intercalation, doping, and delamination, forming such layered MXenes with improved surface and structural properties. In bottom‐up approaches, reliance is more based on molecular self‐assembly, benefiting from atomic utilization with higher control within the product microstructure. For MXenes, processes such as chemical vapor deposition (CVD), template growth, and molten salt synthesis are relatively limited. These include precise control of reaction stoichiometry and layer thickness, achieving uniform large‐area films, and controlling surface termination

#### 2.1.2 Methods for Synthesis of MBene Nanosheets

Likewise, several comparative top‐down and bottom‐up synthesis methods have been reported so far for MBenes (see the panels in Figure 1 and Figure S1, Supporting Information).^[^ ^53^, ^69^, ^88^, ^89^, ^90^, ^91^, ^92^, ^93^, ^94^, ^95^ ^]^ Compared to MXenes, for which acid etching is the most common way to remove “A” layers from their MAX‐phase pairs, a relatively lower efficiency of corrosive acids, such as HF, has been found for the synthesis and exfoliation of MBene nanosheets. This also includes fewer available created MAB phases and stronger bonds between M─B compared to M─C bonds, which makes the selective layer etching harder. Also, the smaller size of boron atoms compared to oxygen or nitrogen leads to a denser structure, which requires a longer etching process or further impedes etching. Finally, there is a lack of a universal etchant agent that can efficiently remove “A” layers for MAB phases. The etching agents, such as HF or lithium fluoride (LiF), are not highly efficient and may damage the boride lattices. Rather, milder acid/basic etchants (e.g., HCl/sodium hydroxide) have proven effective in constructing layered MBenes.

In bottom‐up synthesis, challenges and advantages differ between MXenes and MBenes. In particular, MBenes synthesis requires high temperatures (usually ≥1200 °C) to form M─B bonds.^[^ ^69^ ^]^ Also, boron's high melting point and low reactivity make vapor‐phase deposition difficult. This can lead to the formation of bulk borides or 3D boron‐based crystals instead of layered 2D nanosheets. Controlling their number and phase purity requires extensive optimization. MBenes are mainly synthesized through bottom‐up methods, particularly CVD. Solid‐state reactions also show potential, since the salt acts as a flux. This promotes growth reactions and forms layered boride crystals, which can be controlled to convert into exfoliated nanosheets.^[^ ^69^ ^]^

As can be seen in the adapted figures, several vacancies are reported for fabricating MBene compositions. This encompasses either using MAB‐phase structures (single or double “A”‐layer phases) for mild etching treatments or those routes that rely on using bulk metal boride‐based powder precursors as starting materials for solvothermal defragmentation. The represented schematic and adapted tabulation suggest that, unlike MXenes, the creation of certain MBenes is not straightforward and cannot be obtained in a one‐pot reaction. Rather, to fully etch the MAB phases or thoroughly convert the precursors into MBene nanosheets, two or more steps may be required. However, the construction of 2D MBenes under milder etching conditions has received growing attention.^[^ ^69^ ^]^ The possibility of synthesizing MBenes using greener and more sustainable etching procedures is an advantage for their large‐scale production and for practical applications.^[^ ^73^ ^]^ Indeed, the method's feasibility and the quality of products are the assets.

In particular, when comparing the crystal structure of MXenes with MBenes, significant variations become evident. Despite the MXenes, which are mostly dependent on the number of “M” layers, the crystal structures of MBenes are different. They are classified into different groups, including the compounds of “212” phases (Cmmm crystal symmetry), “414” phases (Immm symmetry), and “222” phases with Cmcm symmetry crystal structures.^[^ ^89^ ^]^ Another group of these materials consists of the “314” structures (Pmmm symmetry), and the other phase in these groups is formed by the “416” structures (Cmmm symmetry). MBene phases can be generated to structure corresponding materials such as CrB/β‐MoB (with a Cmcm symmetry), Ta_3_B_4_/Cr_3_B_4_ type (with Immm symmetry), and V_2_B_3_/Cr_4_B_6_ type MBenes with a Cmcm symmetry. This configuration subsequently leads to the formation of mainly orthorhombic symmetric crystal structures, which can be identified in distinct phases, including MAB, M_2_AB_2_, M_3_AB_4_, and M_4_AB_6_. The hexagonal structures (P63/mmc symmetries) are identifiable in M_2_AC, M_3_AC_2_, and M_4_AC_3_ MXene pairs.

#### 2.1.3 Methods for Conversion of 2D MXenes/MBenes to Their Quantum Dots or Heterostructures

**Figure 2:**
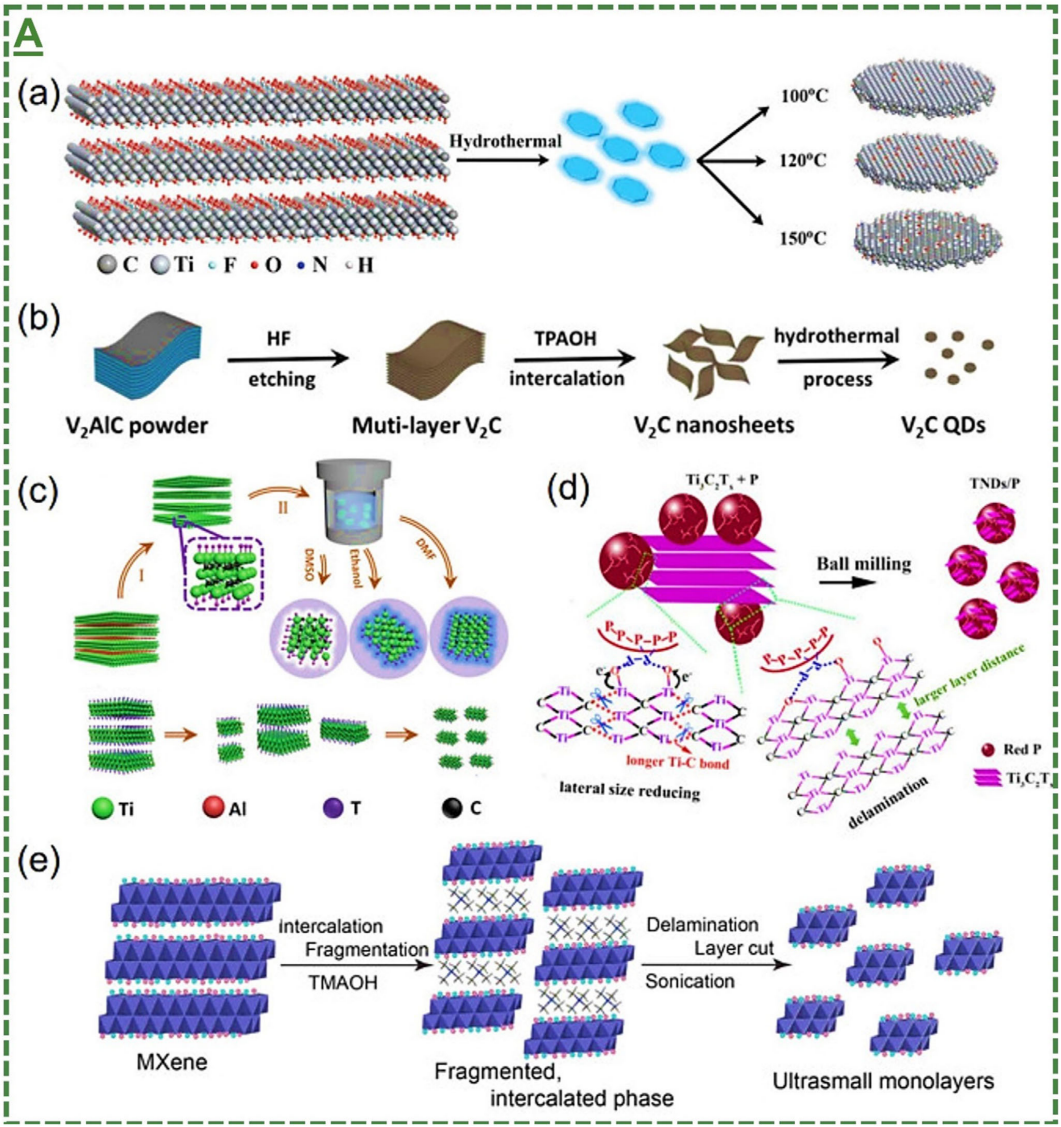
The Schematic illustration of the most common or possible strategies for converting 2D MXenes and potentially MBenes to 0D quantum dots. A) Representation of the reported methods for fabricating quantum dots with stable surface oxides and other surface functionalization routes. The most typical approach is based on hydrothermal treatment, where flake powder of a 3D bulky MAX phase or its 2D MXene nanosheets are treated to reduce their size and ultimately dimension and form quantum dots. A) Adapted with permission.^[^ ^91^ ^]^ Copyright 2024, Elsevier B.V.

Moreover, **Figures** **2** and S2 (Supporting Information) represent some of the reported schematic workflows and atomic structures of distinct MXene nanosheet‐derived quantum dots, and a novel chiral engineering strategy to form highly stable and biocompatible MXene‐based heterostructures. Likewise, different methods have been introduced to fabricate MXenes/MBene quantum dots by breaking down the larger flakes of MXene/MBene blocks into smaller pieces or sizes and reducing their dimension from 3D or 2D to 0D with in situ formation of stable surface transition‐metal oxide layers. Hydrothermal, solvothermal, microwave irradiation, laser/ultrasounds, ball milling, molten salt, intercalation, and pyrolysis methods are frequently used methods in preparing different chemical compositions and sizes of MXene/MBene‐based quantum dots.^[^ ^91^, ^92^, ^93^, ^94^, ^95^ ^]^ These dimensionless materials have been shown to possess enhanced active surface area, optical absorption, colloidal durability, and dispersibility properties in aqueous media compared to their layered materials.

These dimensionless quantum dots have been reported to possess unique physicochemical properties, highlighting their potential for targeted bio‐applications. Additionally, their fabrication offers several advantages, including simple operation, straightforward conversion, relatively fast production, low energy consumption, scalability, and the feasibility of using abundant precursors. However, some applications face method‐dependent limitations, such as harsh reaction conditions, environmental impacts from large‐scale waste, low yields, and batch‐to‐batch variances. The chiral modified Ti_3_C_2_T_x_‐based MXene nanosheets and quantum dots‐derived heterostructures have been shown to possess enhanced stability and biocompatibility properties. Universal efforts are underway to address these challenges and improve synthesis quality. Such progress has broadened the scope of MXenes/MBenes for future investigations. Advances in this area may help bring the technology closer to practical investigation and ultimately real‐world bio‐related applications.

### 2.2 Biocompatibility Comparison and Prediction of MXenes/MBenes for Biotechnological Uses

The field of MXenes/MBenes is expected to further advance, with expanding applications in biotechnology and agriculture. One of the key aspects that promotes their robust bio‐applications is rational synthesis and post‐modification. These optimizations provide inspiration for improving their biocompatibility and reducing their potential nanotoxicological profiles. Because MBenes exhibit relatively higher stability, their biocompatibility is found to be competitive with MXenes, due to the high carbon and nitrogen's chemical similarity with boron. However, a thorough understanding of how MXenes and MBenes interact with biological systems remains necessary before drawing solid conclusions about their properties. Additionally, environmental safety is a key factor in consideration when prioritizing their applications. As mentioned earlier, the current literature on comparing their biocompatibility/nanotoxicity behavior is limited. Therefore, computer‐based predictions can offer a valuable resource for estimating their bio‐related properties.

Technically, a proper computational or chemical toxicity prediction relies on identifying each specific chemical or compound, but standardized IDs or SMILES codes for MXenes/MBenes are not widely available, given their novelty. In this review, using the “ProTox‐3.0” tool as a chemical toxicity predictor to estimate and compare the biocompatibility of available MXenes and MBenes, as well as their closely related or similar compositions, provides insights into their initial toxicity predictions, which can pave the way towards their experimental assessments and safety evaluation for practical applications. As shown in Figures S3–S21 (Supporting Information), the radar and network plots predicted an initial chemistry‐dependent biocompatibility prediction evaluation or toxicity class of these compositions. Notably, these predictions may not fully project the biocompatibility of complex MXene and MBene structures; and they need to be carefully validated by detailed experiments and systemic data in bio‐systems and surrounding environments for each composition.

The predictions of these MXenes/MBenes and related metal‐oxide formulations suggest that they are not generally categorized as toxic compounds (at least at defined dose thresholds and in short‐term interactions or moderate‐period exposures). As can be seen, the majority of predictions show inactivity (marked green) or with low probability (marked pink). However, the precautionary considerations for predicting the materials’ toxicity have been limited to a few specific applications (marked red with different activity intensity). It should be noted that several publications have experimentally shown a high biocompatibility of MXenes and MBenes with various biological systems (Table 1). Likewise, the rationally proposed bio‐interactions and mechanisms of MBenes for several biotechnological applications,^[^ ^89^ ^]^ alongside previous reports for MXenes, it is expected that their bioactive compositions will be the building blocks of future nano‐biotechnology (see Figure S22, Supporting Information). This significant potential is envisioned to be applicable as well in precision agriculture. Thus, robust computer‐assisted and machine learning toxicity predictions (especially those with low toxicity probability) can provide pre‐evaluation on the biocompatibility or toxicity behavior of novel chemicals, paving the way for systemic experimental studies. These predictions, alongside the previously reported biocompatibility evaluations of MXene and MBene‐based materials, can be considered an initial guide for prioritizing the synthesis and optimization of the materials. Obviously, the bioactive properties of each MXene/MBene need to be compared with its akin pair in the same biological model.

## 3 Nano‐Agricultural History of MXenes/MBenes for Plant Protection and Biostimulation

**Figure 3:**
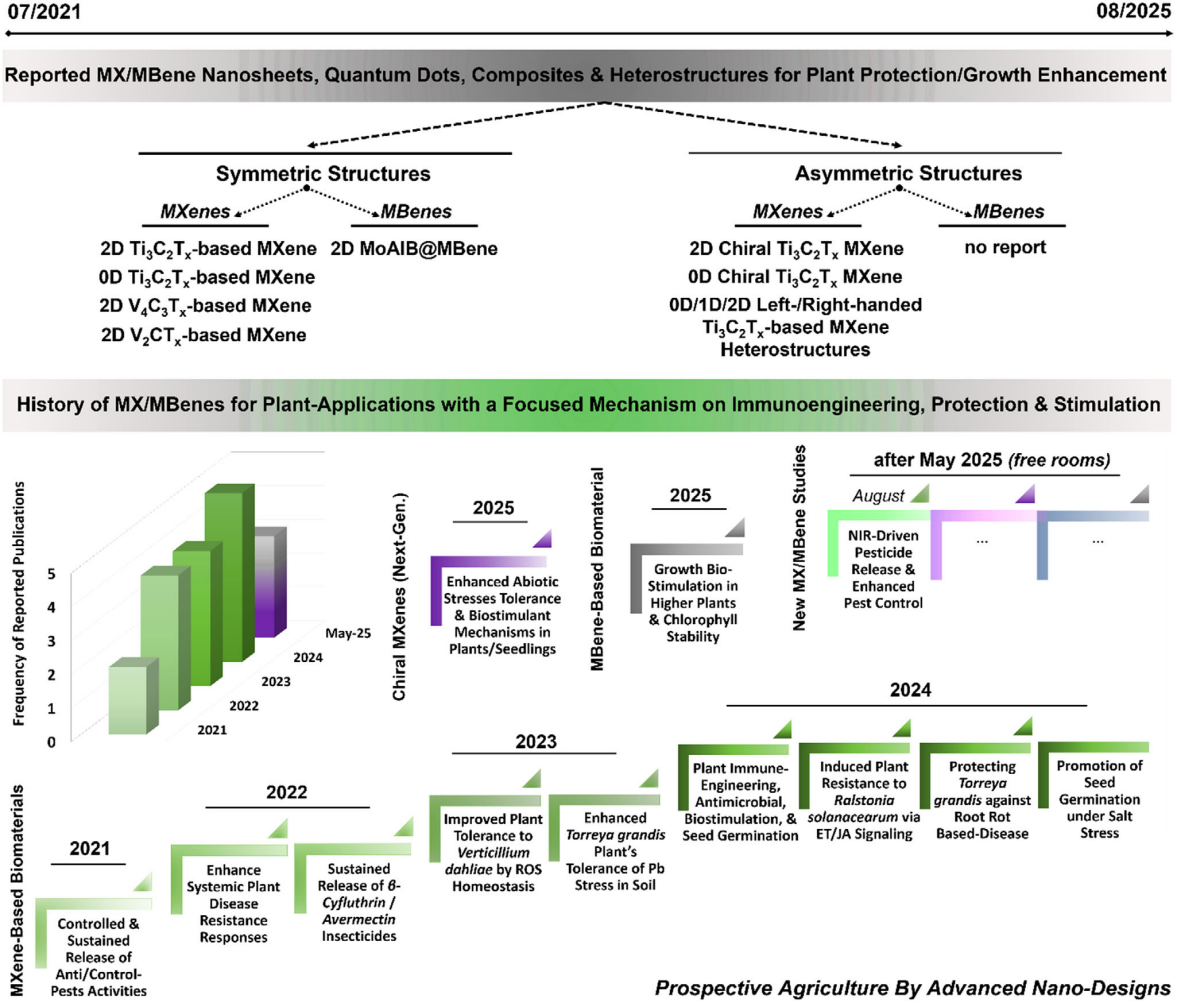
The history of MXene and MBene‐based biomaterials in nano‐agricultural applications.

The roadmap on the potential role of MXenes/MBenes for protecting and biostimulating agricultural plants and crops is displayed in **Figure** **3**. This emerging field of nano‐agriculture has reported significant advancements and research progress in its applications, either alone or in combination for synergistic effects with current agrochemical approaches. In this section, their applications have been discussed in detail based on the review workflow. Every significant reported milestone in tested plants or agricultural‐related models was described in a publication‐time or properties‐based fashion. In particular, starting from 2021‐2022, three pilot studies have uncovered for the first time the impact of MXene nanosheets for anti‐pest/insect‐controlled delivery and sustained release applications of distinct agrochemicals. This literature has been further expanded by our pioneering pre‐print and subsequent publications on novel applications of MXenes for plant immunoengineering and biostimulation. This emerging era has further witnessed other innovative reports on new applications of MXenes/MBenes as next‐generation antimicrobial agents, plant enhancers, innate/adaptive immunity boosters, and seed germination promoters.

### 3.1 Comprehensive Discussions of the Classified Properties/Applications

**Figure 4:**
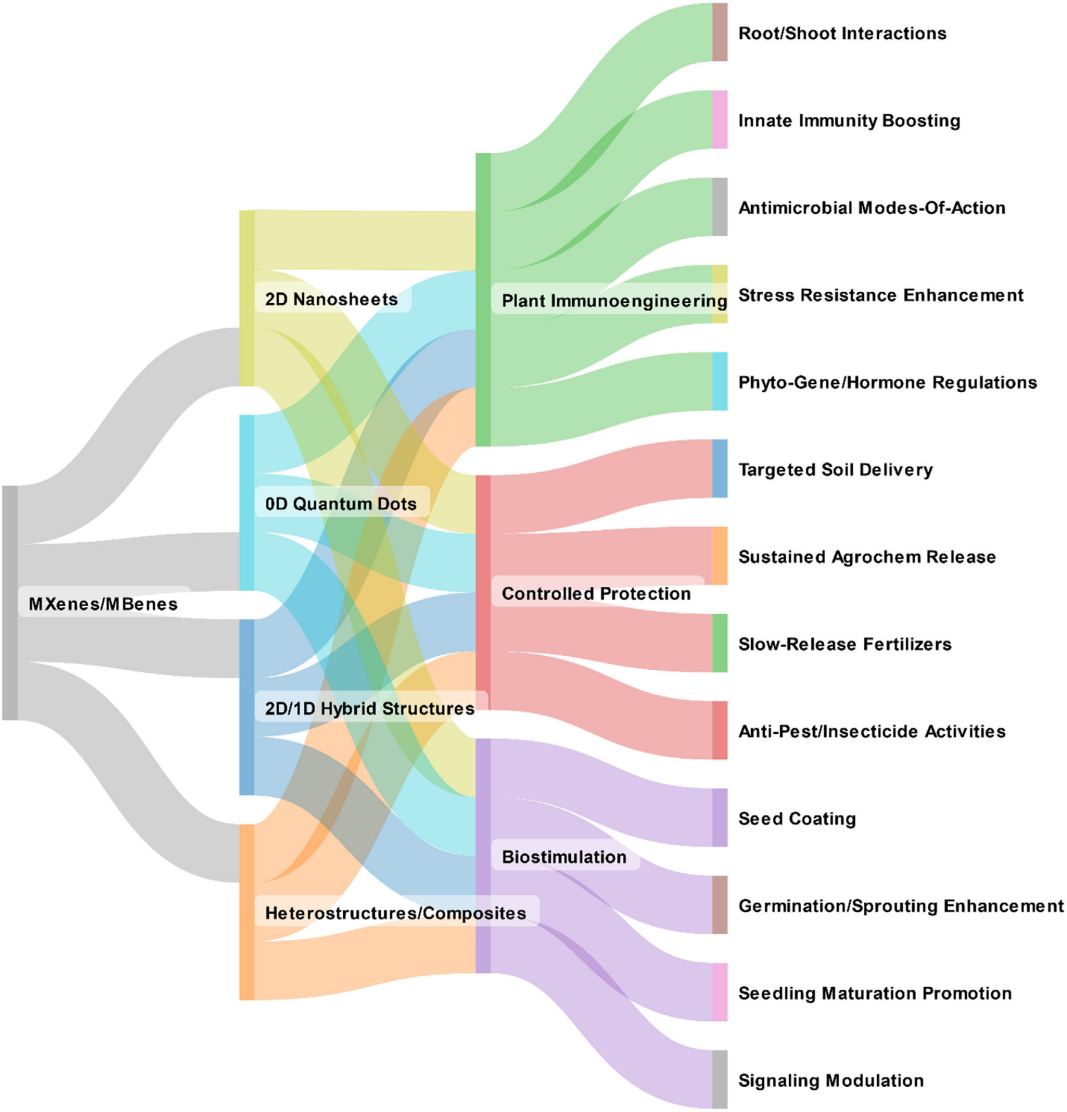
The probability tree diagram of MXenes/MBenes’ roles as plant carriers and enhancers for agricultural applications. This figure presents the overall workflow of this review, including 3 main categories. It covers extensive discussions of related topics in the field and regulations.  It is created by giving equal probability to each material and for application for fair presentation (total frequency of parent to new nodes, summation of all components’ probability in cluster ≈1.0). It was made at “SankeyMATIC” Open Diagram web tool by Steve Bogart (https://sankeymatic.com/).

Based on the workflow in **Figure** **4**, we included a brief introduction for each section, a detailed results and discussion, and an integrated summary highlighting key takeaways. Its tutorial style is then expanded by the current “Agricultural Management Regulations” and considerations for optimizing biocompatibility and functions. We first provided a tabulated summary at a glance (**Table** **2**) to consolidate key parameters, including material type, working doses, delivery systems, release profiles, and bioactivities. Accordingly, the related literature review is described. These detailed discussions enabled effective cross‐comparison between the literature for improved readability. This section also covers the currently existing regulations and future outlooks.

**Table 2:** The summary tabulation of published works on nano‐agricultural applications of MXenes/MBenes for controlled protection, plant immunoengineering, and biostimulation.

Material name/composition	Model/system type	Working dose/loading%	Reported key properties or function(s)	Refs.
Ti_3_C_2_T_x_‐polydopamine MXene nanosheets	In vitro *Emamectin benzoate* (EB) anti‐pest delivery/release	0.0002–2%	Showed pesticide activity with a loading rate of over 45% for sustained release (e.g., 2 weeks) post‐spray	[96]
Ti_3_C_2_T_x_‐based MXene nanosheets	In vitro *Avermectin* (AV) anti‐pest delivery/release	LC (%) = W_AV pesticide_/W_MXene carrier_ ×100	Showed loading capability of over 80% with improved water solubility for pH‐response delivery and slow‐release with high photostability under UV	[97]
Ti_3_C_2_T_x_ MXene hybrid tunic acid	In vitro *β‐cyfluthrin* insecticide delivery/release	N.A.	Showed the capability of a nano‐delivery system with sustained release for improved leaf affinity and insecticidal activity against *Culex pipiens pallen*	[98]
Ti_3_C_2_T_x_‐based MXene nanosheets	In vitro AV pesticide delivery/release	0.5 mg AV in 5 mL acetone with 50 mL of MXene 10 mg mL^−1^)	Improved delivery/slow‐release of AV against the *Spodoptera frugiperda* pest with ≈40% photothermal conversion efficiency	[99]
Ti_3_C_2_T_x_‐containing EMPP/CD@PEG	*Zea mays* (Maize) seedling and plants	0.25 to 1 mg mL^−1^ with 47.5% photothermal conversion	Showed insecticidal activity against *Spodoptera frugiperda*, *Corn borer*, and *Mythimna separata (LC_50:_ 0.080, 0.125, 0.104* mg L^−1^	[100]
Ti_3_C_2_T_x_‐based MXene aqueous colloids	*Arabidopsis thaliana* plant	10‐200 µg mL^−1^	Induced ROS generation for activating prompt eliciting	[65, 101]
Ti_3_C_2_T_x_‐based MXene aqueous colloids	*Arabidopsis thaliana* plant	40 µg mL^−1^	Induced sustained priming response	[65, 101]
Ti_3_C_2_T_x_‐based MXene aqueous colloids	*Arabidopsis thaliana* plant	0.01 to 40 µg mL^−1^	Induced immediate stomatal closure mechanism in plants upon foliar spray	[65, 101]
Ti_3_C_2_T_x_‐based MXene aqueous colloids	*Arabidopsis thaliana* plant	10‐100 µg mL^−1^ (in vitro) 25–100 µg mL^−1^ (in planta)	Showed antibacterial mode‐of‐action in vitro and in planta against *Pseudomonas syringae* pathovar *tomato*‐DC3000	[65, 101]
Ti_3_C_2_T_x_‐based MXene aqueous colloids	*Nicotiana benthamiana* plant	40 µg mL^−1^	Showed antiviral mode of action in planta against TMV	[65, 101]
Ti_3_C_2_T_x_‐based MXene aqueous colloids	*Arabidopsis thaliana* plant	10–200 µg mL^−1^	Showed antifungal activity in vitro against *Fusarium graminearum*	[65, 101]
Ti_3_C_2_T_x_‐based MXene aqueous colloids	*Arabidopsis thaliana* plant	40 µg mL^−1^	Induced biostimulatory responses in plants	[65, 101]
Ti_3_C_2_T_x_‐based MXene aqueous colloids	*Arabidopsis thaliana* plant	40 µg mL^−1^	Enhanced phyto‐hormone/gene expressions	[65, 101]
Ti_3_C_2_T_x_‐ polyethyleneimine‐MXene quantum dots	Gossypium hirsutum* L (Cotton) plant	50 mg L^−1^	Showed efficiency in scavenging RSO with improved plant's tolerance to *Verticillium wilt* caused by *Verticillium dahliae* fungi (root absorption application)	[102]
V_4_C_3_T_x_‐based MXenzymes	*Pisum sativum* (pea) seed	200 mM NaCl and V_4_C_3_ MXene	Showed efficient ROS scavenging for the promotion of seed germination under salt stress	[103]
Ti_3_C_2_T_x_‐based MXene nanosheets	*Torreya grandis* plant	25‐100 mg L^−1^	Improved root rot disease and antifungal activity for higher plant/soil quality	[104]
Ti_3_C_2_T_x_‐based MXene nanosheets	*Torreya grandis* plant	MXene/Pb ≈10–200 mg kg^−1^	Enhanced plant tolerance against Pb stress	[105]
V_2_C‐based MXene nanosheets		12.5‐400 mg L^−1^	Induced plant resistance to *Ralstonia solanacearum* via the ET/JA signaling pathway inducer in crop protection	[106]
E‐Ti_3_C_2_T_x_ MXene nanosheets	*Solanum lycopersicum* (tomato) plant	1200 µg mL^−1^ (10 mL per treatment)	Root irrigation of this MXene did not cause a significant risk to soil beneficial bacteria in the tomato rhizosphere (reversible to normal ratio in 6 days)	[107]
L‐/D‐handed chiral Ti_3_C_2_T_x_‐MXene heterostructure	*Arabidopsis thaliana* seed/seedling	40,100 µg mL^−1^ 88 µg mL^−1^	Induced bio‐stimulatory response for enhanced seed germination, seedling sprouting, and maturation	[66]
L‐/D‐handed chiral Ti_3_C_2_T_x_‐MXene heterostructure	*Arabidopsis thaliana* seed/seedling	88 µg mL^−1^	Induced bio‐stimulatory response for enhanced germination and growth under salt stress	[66]
D‐handed chiral Ti_3_C_2_T_x_‐MXene heterostructure	*Arabidopsis thaliana* plant	100 µg mL^−1^	Enhanced plant tolerance against abiotic conditions (salinity, drought, and light stress) in both greenhouse and climate chamber	[66]
MoAlB@MBene nanosheets	Seedling/plant *L. sativum*, *S. alba*, and *S. saccharatum*	125‐250 mg dm^−3^	Enhanced root/sprout growth of *L. sativum*, *S. alba*, and *S. saccharatum* post‐seed coating alongside increased chlorophylls	[73]

### 3.2 Reported Application(s) for Controlled Delivery and Sustained Release of Pesticides/Insecticides

Nanomaterial‐based drug delivery systems in agricultural applications enable efficient and precise, controlled release of fertilizers, growth promoters, stimulants, and pesticides, minimizing waste and environmental impact. By enhancing stability, solubility, and targeted action, nano‐based substances improve efficiency while reducing chemical exposure to non‐target organisms and promoting sustainable agriculture. As shown, two different studies introduced an innovative application of MXene for controlled delivery and sustained release of agrochemicals to plant/soil media. These studies have reported the pesticide delivery application of titanium carbide MXene (Ti_3_C_2_T_x_)‐based nanosheets as a novel and promising carrier agent for targeted delivery of typical anti‐pest products, including *Emamectin benzoate* (EB) and *Avermectin*. In particular, Wu et al. explored the impact of a hybrid MXene‐based nanomaterial (Ti_3_C_2_T_x_ and polydopamine) for controlled EV delivery to improve its efficiency and environmental impact on agricultural inputs.^[^ ^96^ ^]^

**Figure 5:**
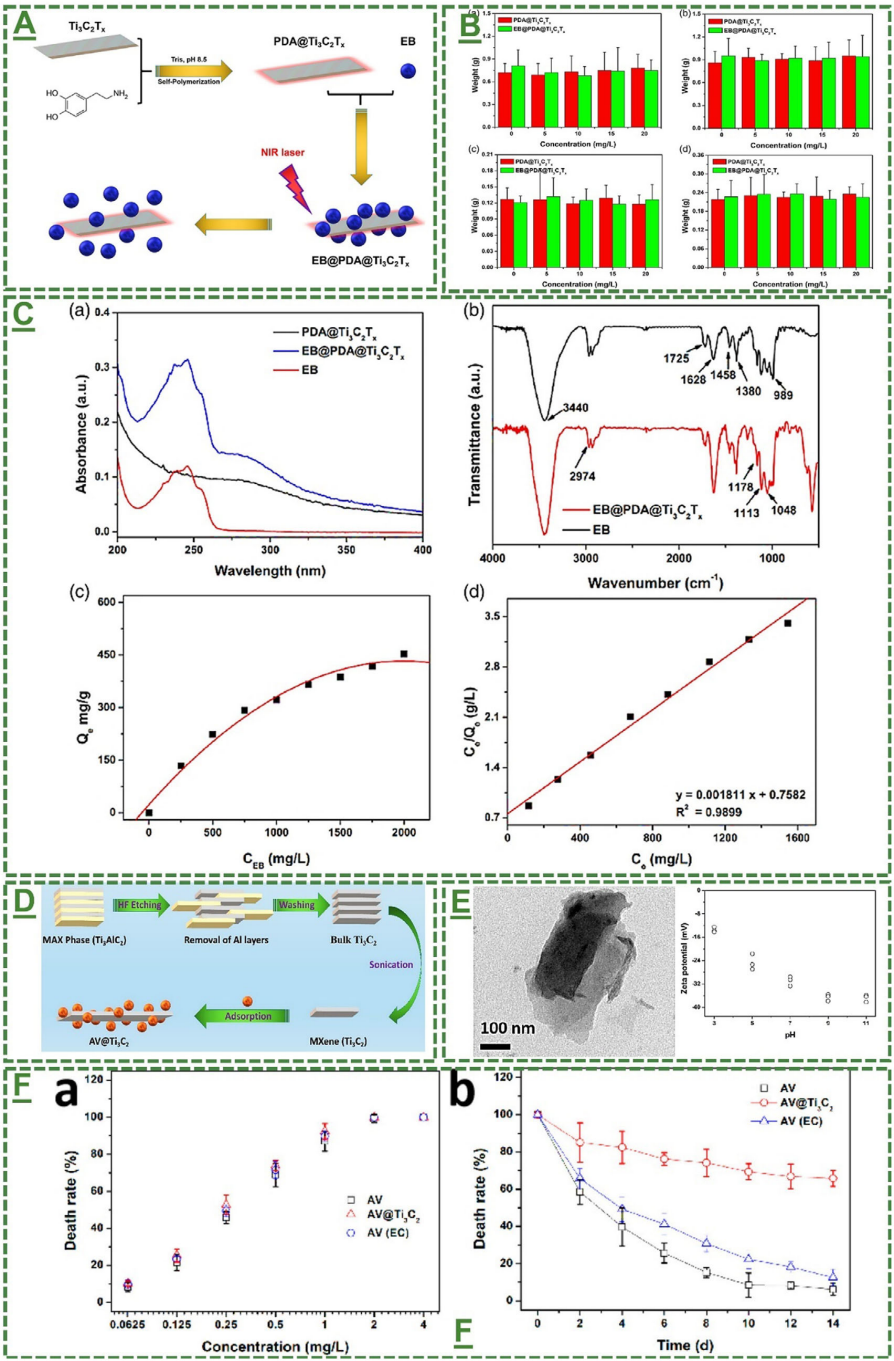
A) Schematic illustration of the study plan for designing and preparing a polydopamine‐modified Ti_3_C_2_T_x_ MXene as an innovative nanocarrier for controlled delivery and sustained release delivery of EB antipest activity. B,C) The physicochemical characterization of this material before and after modification. The UV–vis and FTIR spectra of EB and polydopamine‐Ti_3_C_2_T_x_, and EB‐ polydopamine‐Ti_3_C_2_T_x_ MXene, and the pesticide loading capacity of the hybrid MXene at different concentrations of EB ranging from 0 to 2000 mg L^−1^ with its Langmuir model. It also depicts the release process of EB‐polydopamine‐Ti_3_C_2_T_x_ before and after 10 min NIR treatment at 808 nm and 2 W cm^−2^ for each time point. The germination rate/efficiency of maize seeds and growth of maize seedlings after treatment with polydopamine‐Ti_3_C_2_T_x_ and EB‐polydopamine‐Ti_3_C_2_T_x_ MXene system (*, **, and *** are represented for the *p* values of lower than 0.05, 0.01, and 0.001, respectively, *n* = 3). A–C) Reproduced with permission.^[^ ^96^ ^]^ Copyright 2021, Society of Chemical Industry. D) The schematic of the workplan for preparing a Ti_3_C_2_T_x_ MXene‐AV sustained delivery design. E) Morphology TEM characterization of the prepared formulation and measured negatively charged at different pH levels ranging from 3 to 11. F) the release activity and anti‐pest behaviour of designed systems compared to control samples, based on two factors, including time and dose. D–F): Reproduced with permission.^[^ ^97^ ^]^ Copyright 2021, American Chemical Society.

Their work primarily focused on preparing and enhancing the delivery properties of this material through near‐infrared responsive release mechanisms. The nanosheets of Ti_3_C_2_T_x_ were first surface‐modified with polydopamine and then loaded with EB to fabricate an innovative pesticide‐delivery platform for plant protection. The rationale behind designing this delivery system over conventional applications of EB or other agrochemical pesticides is most likely due to the unique physicochemical/biological properties of Ti_3_C_2_T_x_ and the advantages of its high active surface area and ionic conductivity, along with an enrichment with various bioactive functional groups, which enable improved loading and sustained release properties of pesticides. Additionally, integrating polydopamine as a biomimetic polymer with a known capability for in situ self‐polymerization on surfaces contributed to effectively enhancing the biocompatibility, surface reactivity, and hydrophilicity of the material. The designed system is reported to bond with this pesticide through physisorption, where a weak van der Waals force (non‐covalent) allows the EB's molecules to non‐permanently attach to the surface of modified MXene without any chemicals or enhanced crosslinking/encapsulating methods (see **Figure** **5**).

According to their results, pesticide activity with a loading rate of over 45% was achieved by this hybrid material. In addition, they reported that the as‐designed system could not only improve the loading capacity of EB but also impart the intrinsic photothermal properties of pristine Ti_3_C_2_T_x_ MXene due to its strong ability to absorb near‐infrared light and convert it into heat for enhanced photothermal applications and controlled release of the EB. Indeed, the increase in the local temperature of the pesticide‐loaded carrier can contribute to a sustained release way over several days or a few weeks to significantly reduce the susceptibility of pesticide loss to the environment through water flow and minimize the frequency of required pesticide treatments. To confirm this ability, they performed an in vitro assay to evaluate the efficiency of the as‐designed nanocarrier in terms of remaining active for a sufficient time (e.g., 14 days) towards potential applications for long‐lasting plant protection against specific pests. This strategy holds a massive promise to reduce the health risks, environmental contamination concerns, and farming costs by avoiding the over‐application of pesticides if its nanosafety for agricultural input is robustly confirmed. Notably, as one of the crucial safety aspects of nanocarriers, further detailed mechanisms and long‐term biocompatibility analysis may still be required to validate the robustness of these nano‐enabled approaches. The authors assessed the impact of this nanocarrier and its EB‐loaded composite formulation on seed germination, seed‐to‐seedling transition, and seedling growth, suggesting reasonable biocompatibility without any significant adverse effects on the tested seeds and seedlings. To expand the scope of this application of Ti_3_C_2_T_x_ and other MXenes, further mechanistic studies are required to investigate their biodegradability, clearance, and accumulation in the root/shoot area of plants, as well as the soil, beneficial organisms, and water media to ensure their safety and ecological compatibility for real‐world agro‐practices.^[^ ^96^ ^]^

Soon after, a similar application of MXene is reported elsewhere for improved and sustained delivery of *Avermectin* (AV). In a study by Song et al., a multifunctional Ti_3_C_2_T_x_‐based material has been designed to deliver AV pesticide/insecticide, which is hydrophobic in nature with limited stability and functional capability for slow release, which is required for its maximum efficiency as a pesticide chemical for plant sustained release and protection towards future applications in agriculture.^[^ ^97^ ^]^ In their research, 2D Ti_3_C_2_T_x_ MXene sheets were fabricated and physicochemically characterized by transmission electron‐ and atomic force microscopy before and after loading with AV. They explained that AV has been chosen as a typical pesticide in a delivery model due to its challenging controlled releases and durability properties, and environmental impact. Throughout the established in vitro experiments, they accordingly assessed the release kinetics over time, where the concentration of AV was monitored at specific time points. In a similar trend, they reported efficient and fast adsorption of AV on MXene's surfaces with a maximum loading capacity of over 80%, providing an improved water solubility relative to bare AV, which would be beneficial to improving the function and bioactive properties of the pesticide. They suggested that the as‐designed nanoformulation possesses a pH‐dependent slow‐release behavior with excellent photostability under applied ultraviolet (UV) light irradiation. Their results indicated a satisfactory biosafety property of this material without significant adverse effects or disruption on the germination and growth of *Zea mays* (maize). Their result has highlighted the promising implications of this method for reducing pesticide consumption while improving anti‐pest/pest control efficiency in a representative maize model. Together, these findings provide a high potential capacity of Ti_3_C_2_T_x_ for anti‐pest applications, broadening the MXene's capacity in agricultural sectors for plant protection and improved sustainability by nano‐designs (Figure 5).

In a following work by Wan et al., the surface/structure of Ti_3_C_2_T_x_ MXene was modified with tannic acid as a natural polyphenol, to create another new hybrid nanocarrier material for the novel delivery of *β‐cyfluthrin* for improved sustained release of this common insecticide product.^[^ ^98^ ^]^ Through a controlled and sustained release by the impact of MXene/tunic acid/*β‐cyfluthrin*, it was anticipated that the described approach has the potential to minimize the environmental concerns and safety risks associated with the large‐scale implementation of this anti‐insect agent and improve pest management more eco‐economically, once such nanomaterials have proven sufficiently safe for agricultural applications. In particular, the primary objective of that study was to engineer MXene with a biocompatible natural compound to provide an efficient *β‐cyfluthrin* nano‐delivery system with sustained release for improved leaf affinity and insecticidal activities against *Culex pipiens pallens*, ultimately reducing the health risks to farm workers and costs of multiple applications of this insecticide. Likewise, in the previous study in this arena, the surface modification was applied to increase the hydrophilic behavior of pristine Ti_3_C_2_T_x_ MXene and also provide additional surface terminations that potentially can facilitate the encapsulation capacity of *β‐cyfluthrin* in MXene (over 44% loading rate), likely through hydrogen bonding and/or surface interactions.^[^ ^98^ ^]^ They characterized the morphology and structure of the hybrid material to show its characteristics, confirming the addition of new surface functional groups and phases using scanning electron microscopy, Fourier‐transform infrared spectroscopy, and X‐ray diffraction data.

Further, the loading capacity of this insecticide onto the modified MXene has been determined at different doses, aiming to optimize its loading efficiency, followed by monitoring its release kinetics from the nanocarrier at various pH ranges and over specific time points to fairly assess the controlled release activity of *β‐cyfluthrin* when loaded onto the defined MXene systems.^[^ ^98^ ^]^ Their results suggested an enhanced sustained release profile of *β‐cyfluthrin*, where the applied surface modification has contributed to an effective and gradual release, potentially reducing its initial burst release ratios for more elongated insecticidal impacts. They accordingly proposed a mechanism based on these results that integration of the insecticide with this modified matrix can act as a smart delivery system to effectively minimize fluctuating activity, enabling a more consistent and efficient insect control with a minimum number of required treatments. They have evaluated its biocompatibility with maize and *Vigna radiata* (*Linn*.) *Wilczek* also commented on previous reports, opening a path for its ecotoxicological or nanosafety evaluation as future work.^[^ ^98^ ^]^

**Figure 6:**
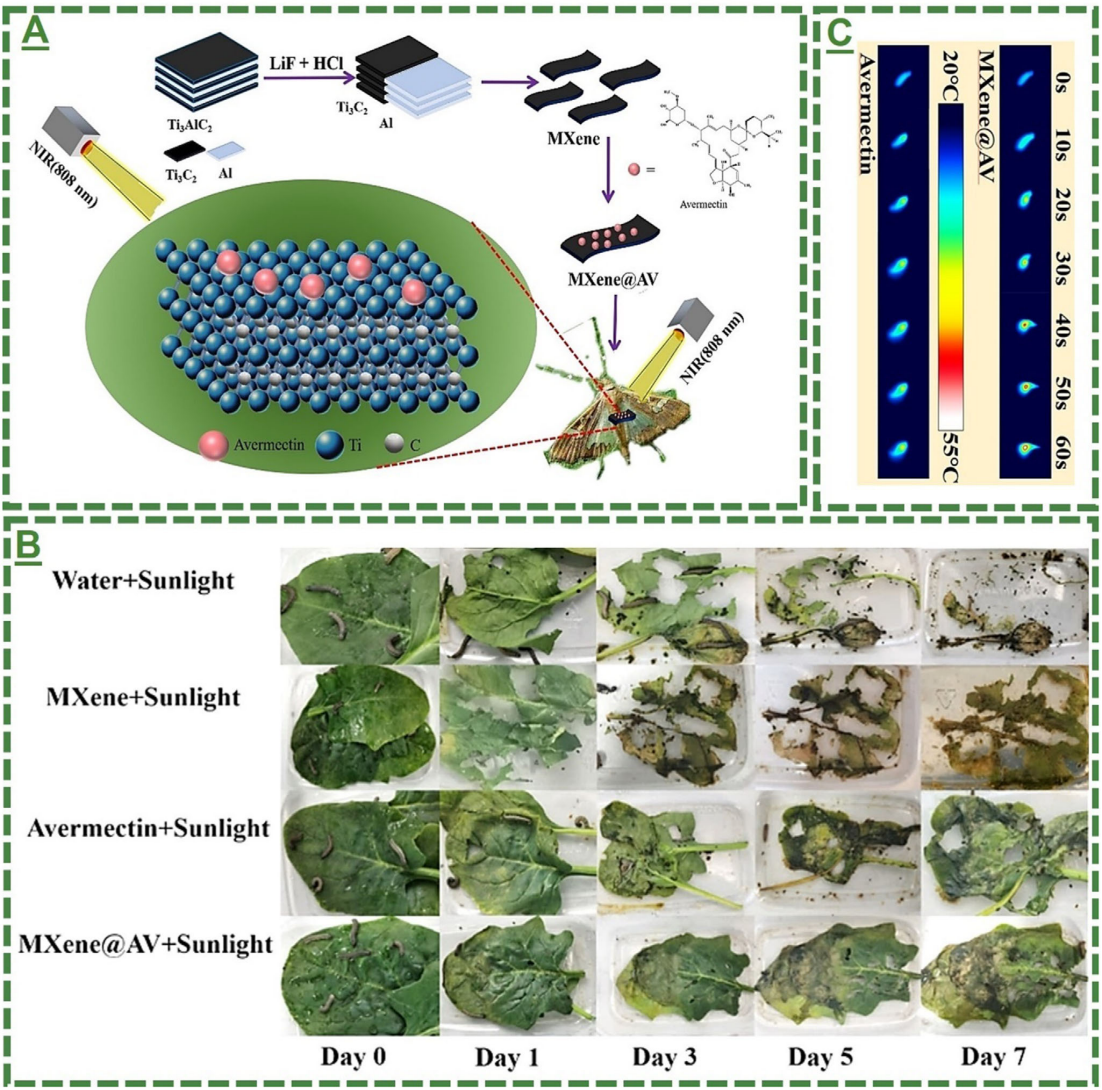
A) Schematic illustration of the study overview on the design, synthesis, and application of MXene‐*Avermectin* Nano‐pesticide formulation. B) Representative *Spodoptera frugiperda* nibbling images on the leaves sprayed with water, Ti_3_C_2_T_x_ MXene, and MXene‐AV under sunlight irradiation. C) Thermal image illustration of the treated *Spodoptera frugiperda* fed with different sprays under 808 nm and the temperature‐rise curve. A–C) Reproduced with permission.^[^ ^99^ ^]^ Copyright 2022, Wiley‐VCH GmbH.

With a similar concept, another study is reported elsewhere by Zhang et al., on the impact of Ti_3_C_2_T_x_ MXene on improving the sustained delivery and controlled release of AV as a common agricultural pest (i.e., *Spodoptera frugiperda*).^[^ ^100^ ^]^ Beyond the typical material preparation and characterization of this prototype material, their results indicate a remarkable reduction in these populations relative to their control groups, indicating the potential and effectiveness of the as‐designed formulation in supporting sustained pest control activities (see **Figure** **6**). Their results suggested the efficiency and stability of the material with high photothermal conversion efficiency (≈40%). They proposed that throughout this combined method, photothermal contact and chemo‐based poisoning to specific pests enable strong lethality and sustained anti‐insect release properties.

In summary, these reports highlight the tunability and versatility of MXene‐based nanocarriers for sustaining pesticide delivery, indicating their high loading capacity, controlled or stimulus‐responsive release properties, and high environmental compatibility. In these works, different MXene formulations, such as AV‐loaded 2D MXene with photothermal properties, tannic acid‐modified MXene for pH‐responsive β‐cyfluthrin delivery, Ti_3_C_2_T_x_ for enhanced anti‐UV property and sustained pH‐response AV release, and finally NIR‐responsive pesticide EB delivery based on modified PDA@Ti_3_C_2_T_x_, have been tailored with improved solubility, efficacy, stability, and enhanced adhesion to plant surfaces, while minimizing pesticide leaching out and stabilizing prolonged pest control. These MXene‐based nano‐systems exhibited minimal toxicity at controlled doses, suggesting a promising, eco‐friendly strategy for modern agricultural pest control. It also paves the way for improving the NIR‐responsive activity for enhancing the photothermal release property.

**Figure 7:**
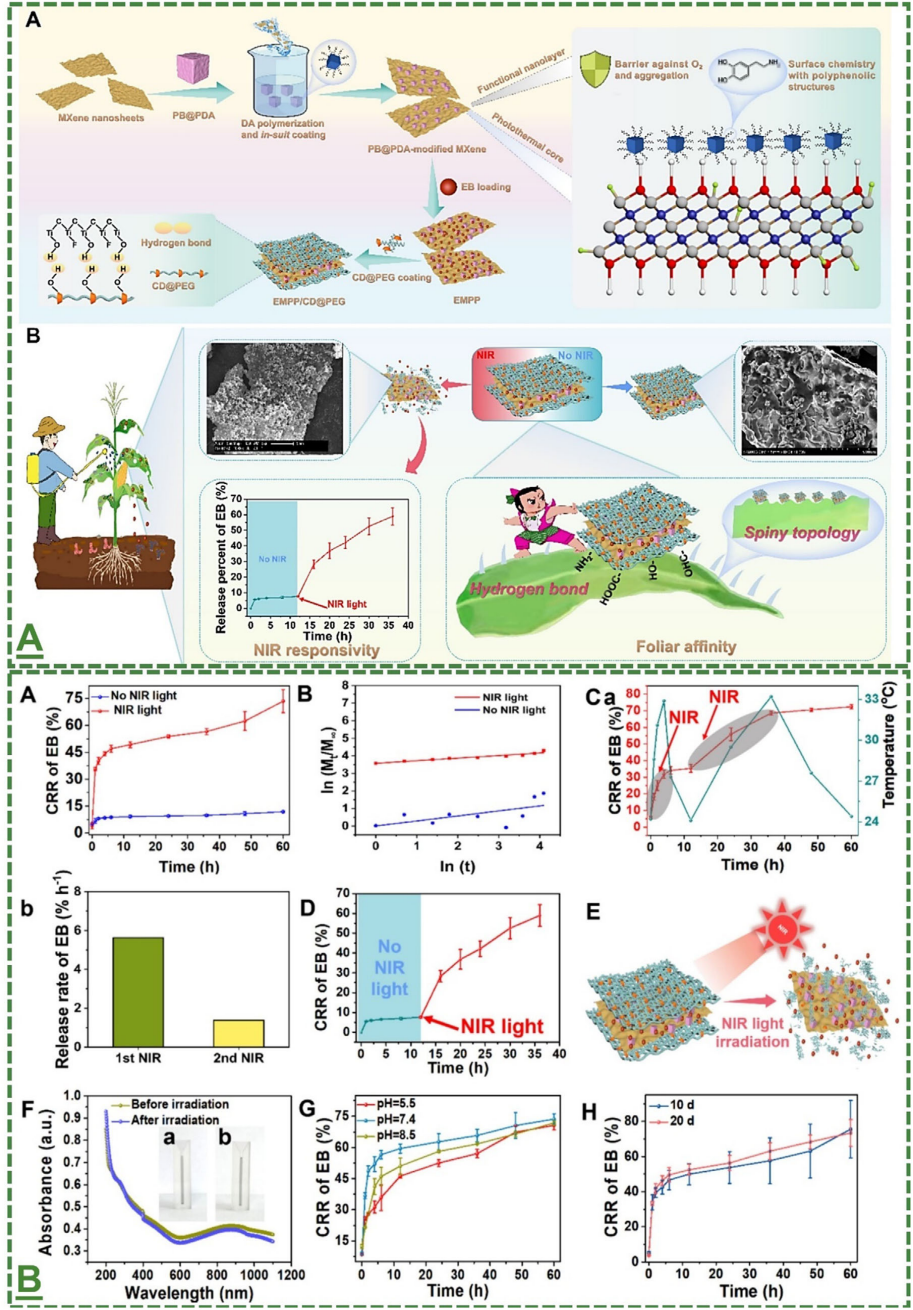
A) Schematic illustration of the study plan for designing and preparing an innovative Ti_3_C_2_T_x_ MXene composite hydrogel for slow‐release and controlled NIR photothermal application. EMPP/CD@PEG MXene nanopatches were prepared, and their properties were evaluated in maize fields. This figure depicts the represented release profiles data of EMPP/CD@PEG under NIR light conditions and dark conditions, as well as the corresponding Ritger–Peppas models. Temperature and EB‐release profiles of the composite within two on/off cycles of light irradiation (under two cycles of NIR irradiation) and its release profile upon the applied sunlight‐to‐NIR light shift. This figure also depicts the UV‐visible‐NIR spectra of this composite dispersion before/after NIR light irradiation (Inset is a camera image composite of the aqueous solution before and after irradiation (left and right). It displays the effects of different pH and time on the release behavior of material. A,B) Reproduced with permission.^[^ ^100^ ^]^ Copyright 2025, Elsevier B.V.

Moreover, building on these reports on NIR‐driven pesticide release applications of Ti_3_C_2_T_x_ MXene‐based nanosheets, a more recent study by Zhang et al has shown the enhanced efficiency. In particular, a flexible sandwich‐like MXene nanosheet‐derived nanopatch structure is fabricated to enhance NIR responsiveness. The composite was engineered by anchoring polydopamine‐functionalized Prussian blue onto Ti_3_C_2_T_x_ layers, followed by loading emamectin benzoate (EB), and subsequently modifying these layers with a temperature‐sensitive cyclodextrin@polyethylene glycol hydrogel.^[^ ^100^ ^]^ They labeled it as “EMPP/CD@PEG”. In particular, the as‐designed material is used as a precise nano‐carrier for pesticide delivery, enhancing its efficiency. Of note, the accordion‐like multilayered structure of these MXene nanosheets was delaminated to form single layers to increase transparency and surface light absorption efficiency. Briefly, the produced photothermal‐active composite (MXene@PB@PDA) was shown to enhance durability through the applied surface‐modification strategy and new hydrogen bond interactions. Loading the EB into this composite material has shown effective encapsulation of this particular pesticide with high loading efficiency and photothermal conversion capacity. In addition, its unique structure offered tight adhesion to leaf surfaces for sustained release and a more elongated anti‐pest effect (**Figure** **7**).

The uniqueness of their study relies on enhancing the photothermal performance of Ti_3_C_2_T_x_ MXenes, as this material has already been shown to possess excellent NIR light‐surface absorption capability. Their data showed enhanced thermal conductivity and also light‐absorption efficiency of pristine nanosheets for targeted photothermal applications.^[^ ^100^ ^]^ They claimed these improvements by enhancing both the roughness and wrinkling properties of the introduced MXene nanosheets‐derived composite hydrogel. Their findings have further pushed the scope of previous MXene reports and improved the efficiency of this material for specific pest‐control applications. In particular, their data showed that its photothermal ability to absorb/utilize incident light is remarkably enhanced, resulting in an overall photothermal conversion efficiency of ≈47% (see Figure 7 for the adapted data).^[^ ^100^ ^]^ Further, the cumulative release rate (CRR) of this agrochemical from the surface of the designed nanopatch was significantly increased to ≈35% within the first hour of exposure to NIR light, and eventually reached a desirable release level of over 70% after 60 hours. This data indicates that a rapid and sustained release profile of MXene‐based nano‐patch is obtained. In contrast, in the absence of NIR light, the CRR has been reported to remain significantly lower at only 11.8%, demonstrating the effective blocking effect of this hydrogel matrix and preventing leaky release.^[^ ^100^ ^]^

Moreover, the wetting ability and foliar retention of pesticide formulations are crucial factors for the adherence and biological uptake of pesticides on foliage. This MXene‐based nanopatch demonstrated superior leaf‐wetting capacity, with a low contact angle of ≈35 ° on maize leaves compared to deionized water (≈77 °) and conventional EB technical concentrate (EB TC, ≈74°) This enhanced wettability and reduced contact angel was linked to the lamellar structure of the nanocarrier and the presence of phenolic hydroxyl groups, which facilitated hydrogen bonding with the waxy layer of the leaf (please see the original citation of this data). Field‐applied nanopesticides unavoidably encounter varying rainfall intensities, which are a critical factor impacting their prolonged pest control performance. The SEM images of this nanopatch indicated that the material remained attached to the leaf surface even after repeated water rinsing, confirming excellent wash‐off resistance (Figure 7). Their chemical composition data validated the presence of titanium, carbon, nitrogen, and iron, suggesting the persistence of the materials post‐rinsing on the foliage.^[^ ^100^ ^]^

**Figure 8:**
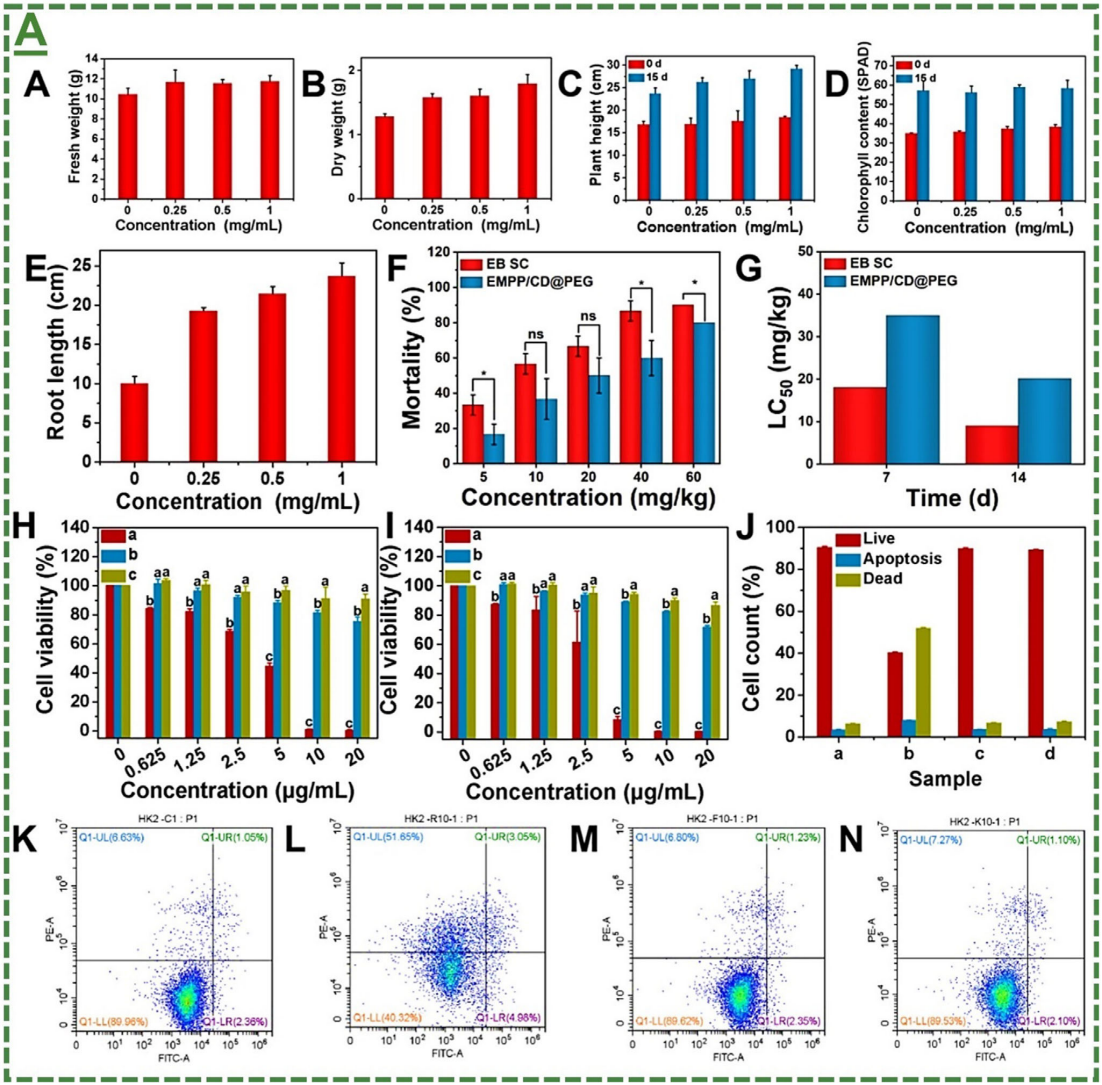
The data represented in this figure show, in order, the fresh and dry weights of maize seedlings treated with various doses of EMPP/CD@PEG after around two weeks (15 days). It also depicts the height, chlorophyll content, and root length of these seedlings at day 15. The Mortality and LC50 values of earthworms treated with these samples are also demonstrated. The viability of HK‐2 cells incubated with different doses of EB SC, unloaded‐EB EMPP/CD@PEG, and EMPP/CD@PEG at day 1 (post 24 and 48 h, respectively). It also depicts the reported apoptosis rate and corresponding flow cytometry analysis of the treated HK‐2 cells by blank control, EB SC unloaded‐EB EMPP/CD@PEG, and the EMPP/CD@PEG at a dose of 10 mg L^−1^ after 24 h (“Q1‐UL”: necrotic cells; “Q1‐UR” and “Q1‐LR”: apoptotic cells; “Q1‐LL”: live cells). A) Reproduced with permission.^[^ ^100^ ^]^ Copyright 2025, Elsevier B.V.

Moreover, the efficacy and insecticidal activity of this patch against *Spodoptera frugiperda*, *Corn borer*, and *Mythimna separata* were measured under NIR irradiation. In a dose‐dependent manner, the as‐designed structure exhibited higher mortality against three tested pests in comparison to conventional EB‐based formulations.^[^ ^100^ ^]^ As can be seen in this figure, the obtained superior insecticidal property has been reported to be because of controlled NIR‐responsive release performance, along with enhanced leaf retention and wettability. Notably, the EMPP/CD@PEG maintained high pest mortality (≈83%) even 12 days after application against *Spodoptera frugiperda* larvae, compared to its suspending concentrate (EB SC: ≈36%), confirming its long‐lasting effect for this particular application. Furthermore, the biocompatibility profile and initial safety assessment of this nanopatch were systematically evaluated in maize seedlings over a 15‐day interaction. Their results indicated that treating with this material did not cause any significant adverse effects on overall plant growth, thereby affirming the nanocarrier's favorable biocompatibility with the tested plants (see **Figure** **8** for the adapted data). To further investigate its ecological safety, particularly concerning non‐target soil organisms, acute toxicity assays were conducted on earthworms. The median lethal concentration values after one and two weeks of exposure indicate a substantial reduction in toxicity to the earthworm when EB is encapsulated within the EMPP/CD@PEG matrix compared to the conventional EB SC.^[^ ^100^ ^]^

Given the possibility of nanomaterials uptake and translocation within crop plants, and the potential subsequent migration into the human body via the food chain, the authors have also assessed the in vitro cytotoxicity of the material using HK‐2 cells. In contrast to EB SC, which induced a significant dose‐dependent decrease in cell viability, treatments with EMPP/CD@PEG and MXene@PB@PDA did not cause any significant adverse effects on the viability of tested cells for up to 48 hours of incubation (Figure 8). Taking all these accounts into consideration, they have suggested biocompatibility of the EMPP/CD@PEG with tested plants. Their results also suggested that treating with the material induced minimal toxicity to non‐target organisms in the soil, and negligible toxicity to the tested human cells. This study and the previous reports on the sustained release and pest control function of Ti_3_C_2_T_x_, highlight its potential in developing next‐generation precision agriculture platforms that combine targeted delivery and reduced ecological effects.

### 3.3 Reported Application(s) for Enhanced In Vitro/In Planta Reactive Oxygen Species Production, Plant Alertness/Strength, and Antimicrobial Bioactivities Against Phytopathogens

Oxidative stress is one of the main mechanisms triggered by biotic or abiotic stress factors, which arises from the excessive accumulation of reactive oxygen species (ROS). ROS function as signaling molecules that enhance the plants’ immunity and activate defense‐related pathways through activation of several defense‐related proteins. The oxidative burst initiates the expression of antioxidant enzymes and non‐enzymatic antioxidants, thereby modulating redox homeostasis. ROS signaling network initiates the activation of SAR and ISR, enhancing the plant's resilience to subsequent stress events. Moreover, priming is a sophisticated strategy that prepares plants to respond more quickly and robustly to biotic and abiotic stress through pre‐exposure to the stress factor stimuli, without additional costs. In this process, the priming active agents—such as nanomaterials—modulate transcriptional, epigenetic, and metabolic states, to establish a “memory” of exposure stress factor. In agriculture, priming represents a sustainable approach to improve crop resilience, minimize chemical inputs, and enhance the product yield under different conditions.

The field of MXene in this particular stream has been further expanded by our pilot works, where for the first time, we introduced in 2022 that surface‐modified Ti_3_C_2_T_x_ MXene nanosheets could effectively enhance systemic plant disease resistance to different phytopathogenic infections, including a triple antiviral/antibacterial/antifungal mode‐of‐action and priming inducing activity in plants to enhance their alertness and biotic stress resistance to phytopathogenic infections.^[^ ^101^ ^]^ In particular, we applied a facile and environmentally friendly protocol by applying ultra‐sonication and one‐pot hydrothermal autoclave treatment to form mixed‐dimensional Ti_3_C_2_T_x_ MXene nanosheet‐derived quantum dots and table phases of surface titanium oxide nanoparticles (e.g., TiO_2_) in an aqueous colloidal dispersion system. The physicochemical properties of this biomaterial have been thoroughly characterized and followed by biocompatibility assessments in *Nicotiana benthamiana* and *Arabidopsis thaliana* plants at different time points. In particular, we demonstrated through pre‐print data that applying relatively low doses of modified Ti_3_C_2_T_x_‐based aqueous colloidal dispersions (≤50 µg mL^−1^) on plant leaves could significantly promote phyto‐stimulation mechanisms in one‐time foliar‐sprayed plants as a novel elicitor and priming nanoagent.

This innovative treatment leads to the production of reactive oxygen species (ROS) as a positive plant defense response promptly upon foliar application with a small volume of these concentrations (≈1 mL) and primes them to systemically enhance their strength for more defense resistance in a more sustained manner and for longer durations. These findings suggest promising implications for using MXene as a future plant vaccine in agriculture. In vitro and in planta, we demonstrated that this biomaterial possesses the intrinsic capability of boosting the innate immunity in treated phyto‐cells/plants to induce enhanced resistance and protect *Nicotiana benthamiana* against tobacco mosaic virus (TMV) and *Arabidopsis thaliana* from infection by *Pseudomonas syringae* (Ps). This study also presented the first in vitro evidence of the impact of this material on decreasing the growth and proliferation of phyto‐fungi (*Fusarium graminearum*), highlighting its multi‐antimicrobial functions in different plant systems. We concluded on this initial assessment that surface‐modified Ti_3_C_2_T_x_ MXene can remarkably promote multi‐stage mechanisms, enhancing their resistance to pathogenic attacks and subsequent diseases/infections through the modulation of their immune responses, presenting a sustainable and efficient method for improving plant health to biotic stresses and protecting them against invasive pathogenic microorganisms. It has set paradigms in the application of modified Ti_3_C_2_T_x_ as a nanocarbon‐based plant booster to safeguard the plant against infections while reducing reliance on the application of agrochemicals, paving the way for newer plant‐disease management strategies (data in section 3.4).^[^ ^101^ ^]^

### 3.4 Reported Application(s) for Induced Plant‐Immunoengineering, Stomatal Closure, Eliciting, Priming, and Biostimulation Mechanisms

Building on these preliminary findings, we thoroughly expanded our experiments to include new plant bioassays and mechanistic studies, aiming to understand the mechanisms underlying the bioactive properties of these surface‐modified MXene nanosheets. In an extensive publication, we further elucidated the impact of this rationally modified Ti_3_C_2_T_x_‐based design on engineering the immune and growth responses of various model plants.^[^ ^65^, ^108^ ^]^ In particular, we further evaluate how this surface‐modified Ti_3_C_2_T_x_ MXene interacts with seeds, seedlings, and plant systems at different concentrations and treatment time points, showing their enhanced phyto‐compatibility and activity. In short, the primary goals were to increase the active surface area of this multifunctional material and minimize its probable nano‐toxicity effects on seeds or living plants, likely by reducing the sharpness of nanosheets’ edges and improving excessive oxidation and structural debonding, which are influential in long‐term interaction and bio‐applications efficiency of MXene dispersions. Thus, in a facile, straightforward, and universal protocol, magnetic stirring and bath‐sonication were used to disperse the accordion‐like nanosheets in aqueous dispersions and further expand the layers to form different flake sizes of Ti_3_C_2_T_x_ multi‐, few‐, and single‐layered particles.

**Figure 9:**
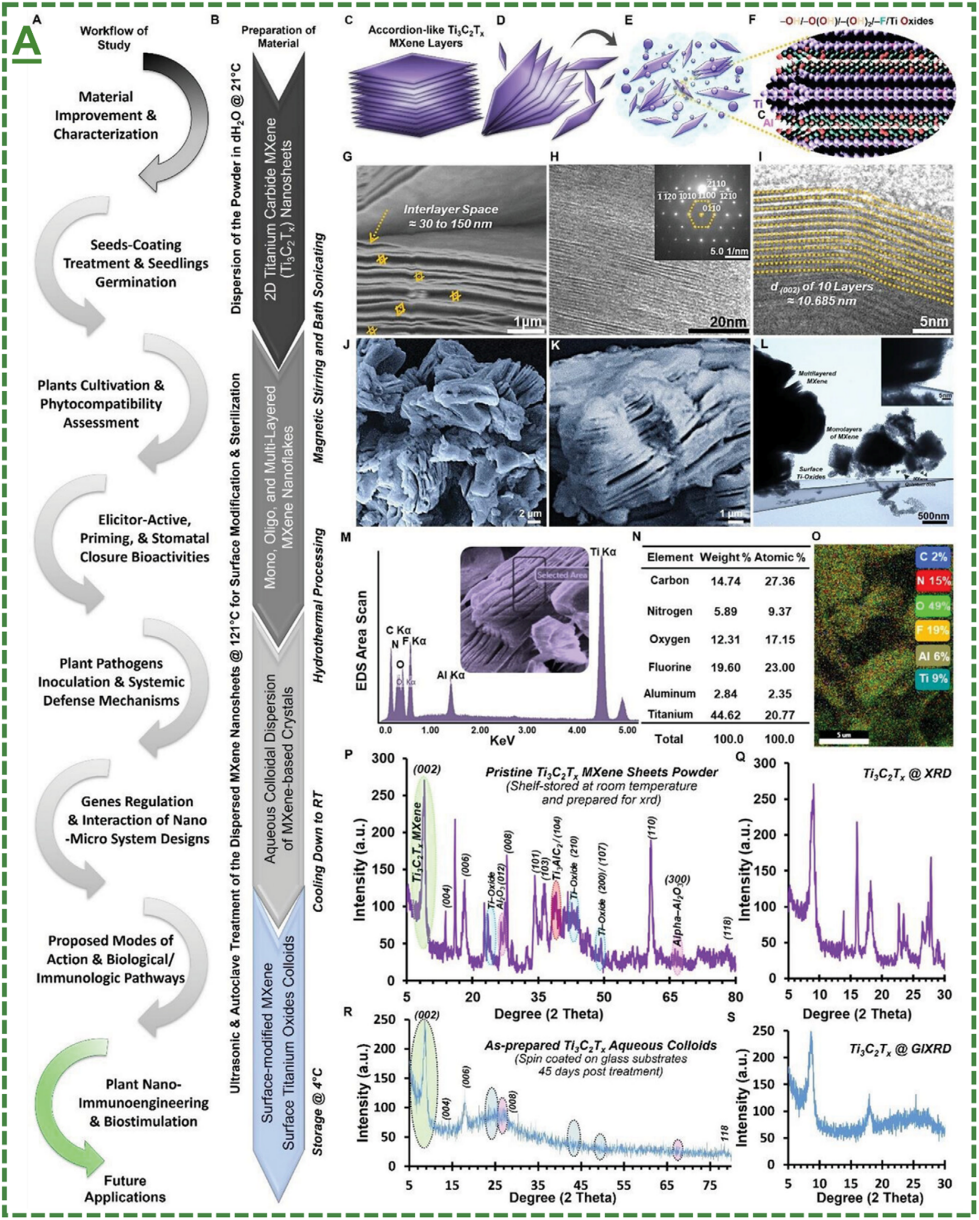
A–S) Representation of the schematic model depicting the study's workflow, preparation procedures, and physicochemical properties characterization of surface‐modified Ti_3_C_2_T_x_ MXene aqueous dispersions. It represented the electron microscopic (SEM/TEM/HRTEM) images of these nanosheets (interlayer distance of ≈30 to 150 nm), derived from the mixed‐low‐dimensional material, and its corresponding selected area electron diffraction (SAED) pattern of a hexagonal crystalline material. SEM images include the morphology of these partially oxidized MXene sheets (shelf‐stored powder at room conditions) and after the applied autoclaved and spin‐coated/dried on the holder. It also represented the TEM images of the nanosheets before and after the applied treatment, depicting the multi‐components morphology of this material (2D Ti_3_C_2_T_x_ sheets, self‐derived 0D MXene quantum dots, and stable surface titanium oxides particles). The EDS area‐scanning and elementals‐mapping and X‐ray diffraction (XRD) analysis of pristine MXene and GIXRD of as‐treated MXene colloidal dispersions, showing the characteristic peak (002) of MXene in their GI/XRD spectra of these samples, including the peaks attributed to the Ti_3_AlC_2_ MAX‐phase precursor, titanium carbide/oxide, and other related metal oxides (low intensity) in its structure, such as α‐Al_2_O_3_. A) Reproduced with permission.^[^ ^65^ ^]^ Copyright 2024, Wiley‐VCH GmbH.

To further improve the microstructure, degradation stability, and colloidal dispersibility of these nanosheets, a one‐pot environmentally friendly hydrothermal autoclave treatment at ≈121 °C for 30 min was performed to create and decorate stable surface titanium oxide nanoparticles on and between MXene layers, constructing a mixed‐dimensional Ti_3_C_2_T_x_‐based heterostructure with enhanced physicochemical/biological properties. The sterilized aqueous colloidal dispersions have been shown to contain 2D MXene sheets, 1D metal oxides, and 0D MXene quantum dots in a single material composition with high stability for months at four degrees. **Figure** **9** represents the study's workflow and physicochemical characterizations of this surface‐tailored MXene for the targeted plant immunoengineering and stimulation applications. To this end, the biocompatibility of the prepared material was assessed with different plant‐based models, followed by the evaluation of its bioactivity properties and underlying mechanisms.

The modified Ti_3_C_2_T_x_ MXene showed high biocompatibility with the seeds, seedlings, and plants of *Nicotiana benthamiana* and *Arabidopsis thaliana* at tested concentrations (0‐100 µg mL^−1^) at different time points for up to 8 days in seedling and around one month in planta in terms of seed‐to‐seedling transition, seed sprouting/germination, seedling maturation, and plant overall growth. In addition, treating the seeds as spontaneous seed‐coating capability of this material and foliar‐spraying plants (single treatment) could effectively enhance the germination and maturation of seeds and immunoengineered the plant innate defense responses by different prompt eliciting, longer‐term priming, and robust stomatal closure‐induced mechanisms to safeguard the average plants against pathogens by increasing their alertness/strength/resistance through modulating the expression of their defense/growth genes (e.g., “jasmonic acid oxidase‐4 gene”, “plant defensin 1.2 gene”, “nonexpressor of PR genes 1”, “enhanced disease susceptibility 1”, “receptor‐like protein 23 gene” and “pathogenesis‐related‐1 gene”) and hormone regulations (a significant upregulation of jasmonic acid (JA) alongside a temporary suppression of salicylic acid (SA)) in tested models. Indeed, this bioactivity aligns well with the principles of plant responses, where a significant upregulation of one of these pathways (JA and SA) can lead to the subsequent downregulation of the other (SA suppression in this particular case) in order to maintain balance in its biological processes.

**Figure 10:**
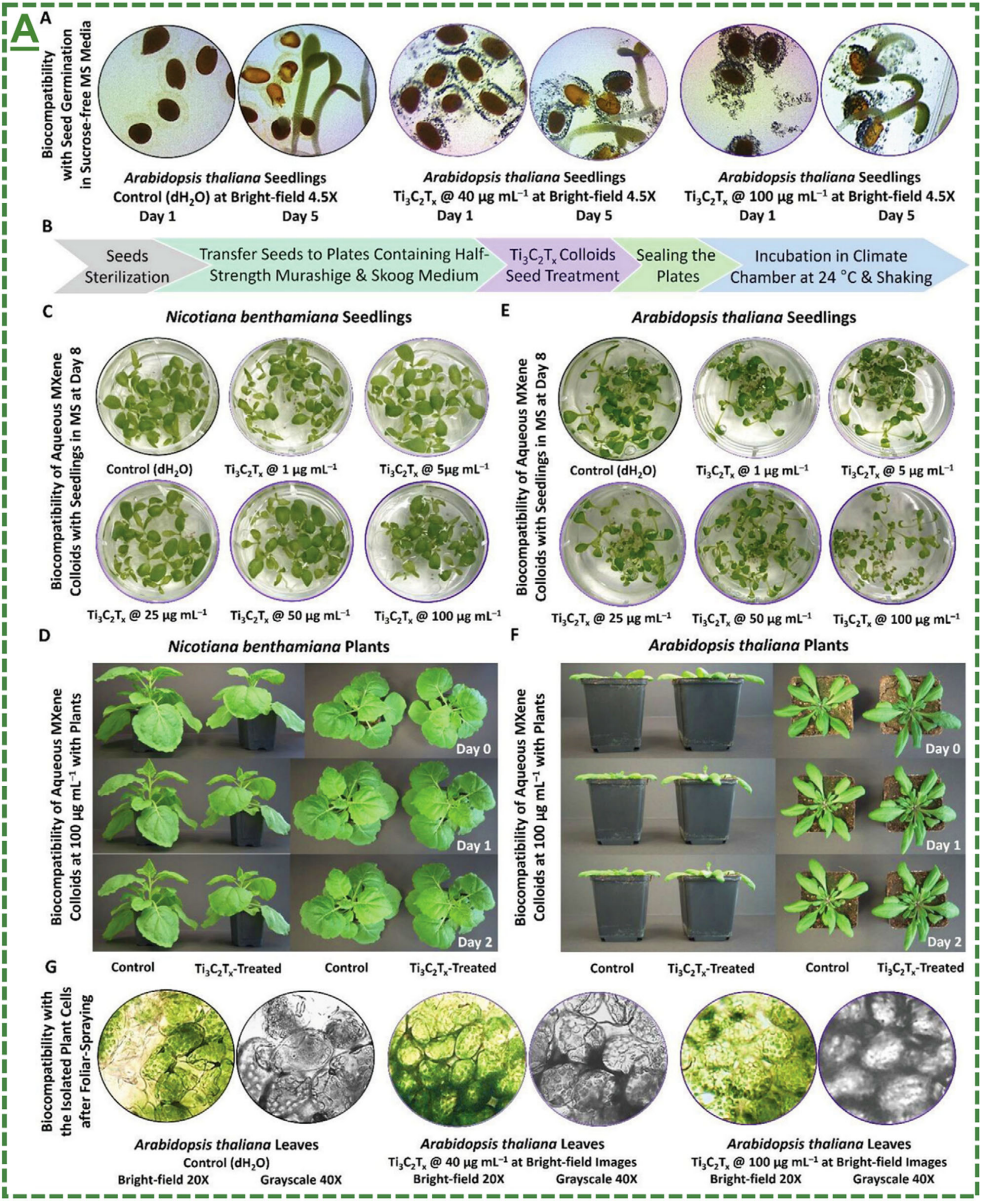
A) Assessment of phyto‐compatibility of Ti_3_C_2_T*_x_* MXene‐based aqueous colloids with seed‐germination, seedling maturation, and plants. Seed coating and seed‐to‐seedling transition with/without these colloids at different concentrations in sucrose‐free MS media (*n* = 10–20). The timeline and biocompatibility evaluation of material with *Nicotiana benthamiana* and *Arabidopsis thaliana* seedlings at doses, ranging from 1‐100 µg mL^−1^ in seedlings (*n* = 20–30) and 100 µg mL^−1^ in plants. Microscopic images of the cells isolated from foliar‐sprayed plants with/without MXene (100 µg mL^−1^, *n* = 5). A) Reproduced with permission.^[^ ^65^ ^]^ Copyright 2024, Wiley‐VCH GmbH.

**Figure 11:**
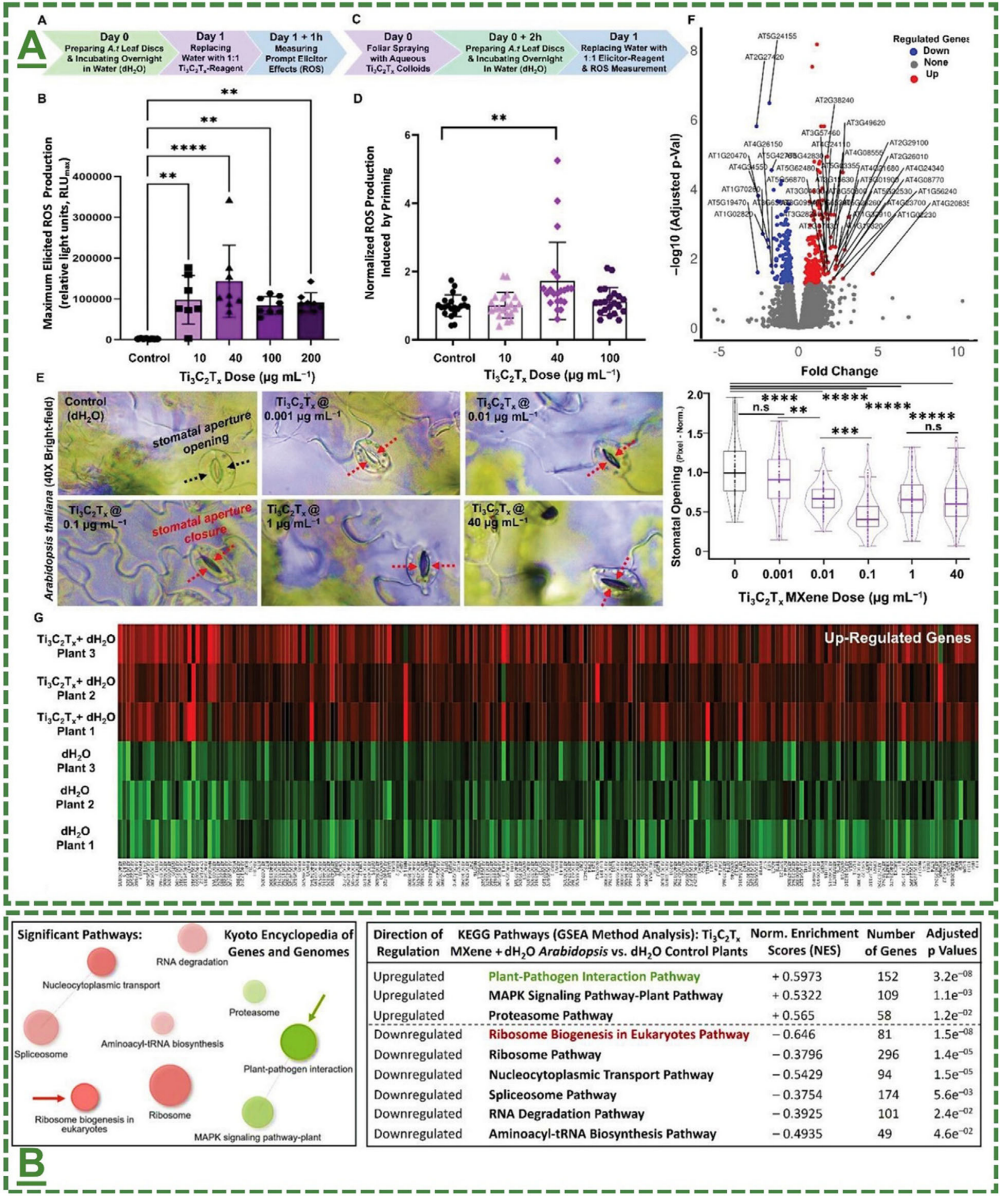
A,B) The data represented in situ ROS production, eliciting, priming, gene expression, RNA sequencing, and induced stomatal closure bioactivities of these MXene aqueous colloids. The observed up‐ and down‐regulated KEGG (Kyoto Encyclopedia of Genes and Genomes) pathways from the RNA sequencing of the treated plants with this material, suggesting a growth‐defense regulation and immunomodulation response of the plants induced by the impact of foliar‐sprayed Ti_3_C_2_Tx MXene. The tabulation pathways of this bioactivity of the material, which was generated based on the obtained RNA‐Seq data from the iDEP Data analyzer in reference to its citation (http://bioinformatics.sdstate.edu/idep/) for that specific upregulated KEGG pathway, which has been identified as a secondary impact of the material on immunoengineered plants at 24 hours post‐treatment. The KEGG over‐representation of the up‐regulated genes in treated *Arabidopsis thaliana* with MXene at 40 µg mL^‒1^ and distilled water in control groups (FDR < 0.05 and log_2_ ≥ 1). This is in coordination with the primary impact of the material on regulating the defense‐related genes and innate immunity observed at 2 h post‐treatment. A,B) Reproduced with permission.^[^ ^65^ ^]^ Copyright 2024, Wiley‐VCH GmbH.

**Figure 12:**
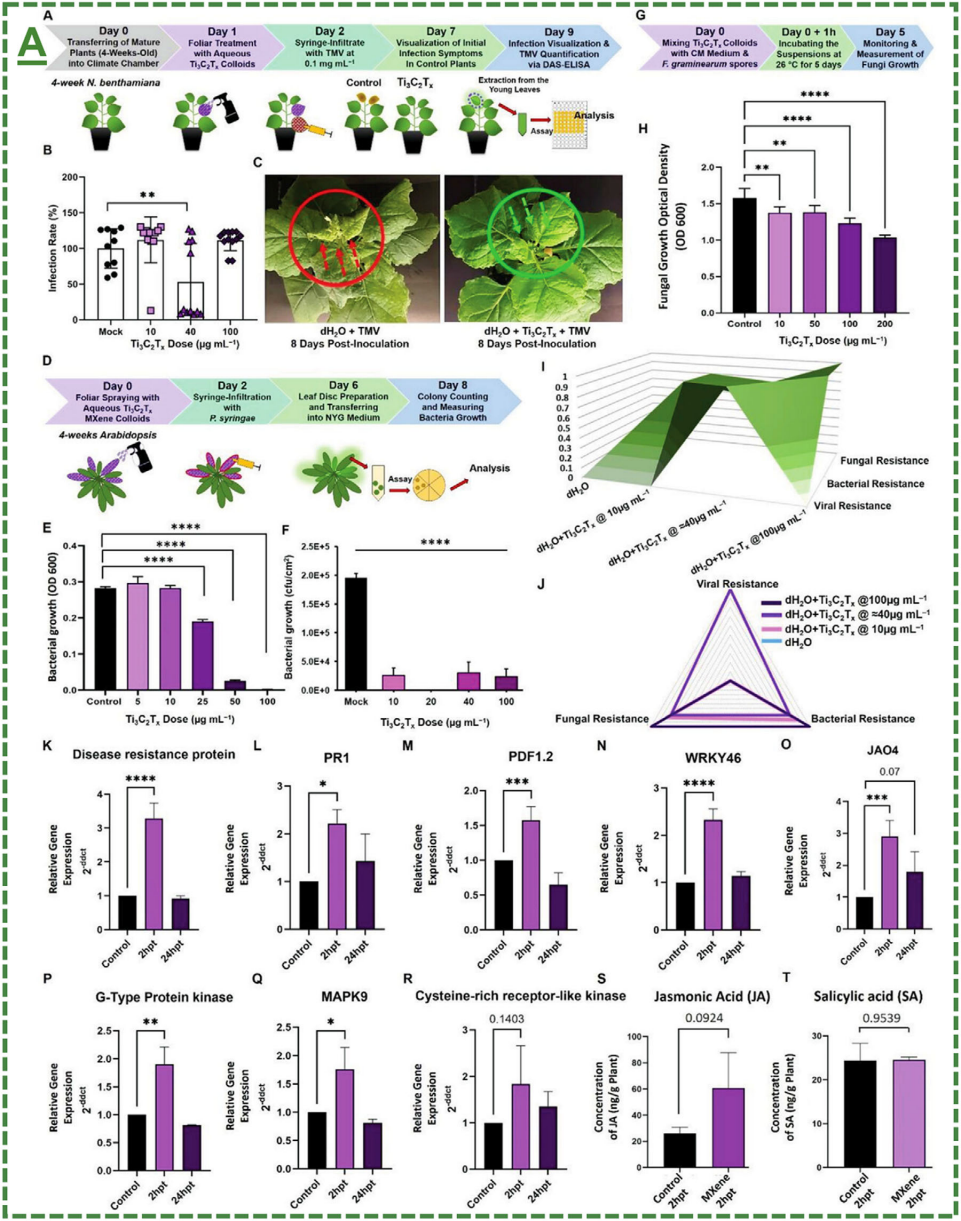
A) The represented experimental processes and multiple antimicrobial modes of action assessments of these Ti_3_C_2_T_x_‐based colloidal dispersions in vitro/*planta*, as well as representation of gene/hormone regulation analysis using RT‐qPCR and mass spectroscopy measurements in as‐treated *Arabidopsis thaliana* plants at 2 h post‐foliar‐treatment. Antimicrobial effects include viral resistance of *Nicotiana benthamiana* plants against TMV infection after treatment with this MXene at an optimal dose of 40 µg mL^−1^ and day‐8 post‐virus‐inoculation (*N* = 2, *n* = 10–12). Further, antibacterial assessments of these MXene dispersions at different doses up to 100 µg mL^−1^ against *Pseudomonas syringae* pathovar *tomato*‐DC3000 in *Arabidopsis thaliana*. It also represents a dose‐dependent antifungal activity against *Fusarium graminearum*. The represented RT‐qPCR related to defense genes was upregulated in these plants at different treatment times of 2 and 24 h post‐treatment compared to control plants in the control groups. Phytohormone measurements of JA and SA in the as‐treated plants with these MXene‐based aqueous dispersions compared to control plants at 2 h post‐spraying (*n* = 3, each from nine leaves cut from five individual plants). A) Reproduced with permission.^[^ ^65^ ^]^ Copyright 2024, Wiley‐VCH GmbH.

Our biocompatibility assessments showed that the direct interaction of this MXene with tested seeds, seedlings, and plants (shoot/root areas) did not cause any significant or visible adverse effects in their overall germination, growth, and development of models (**Figure** **10**). Rather, applying a relatively low dose of this material to phyto‐systems is shown to activate/promote multiple biological and positive immune‐related pathways towards enhancing their systemic defense‐related activity for taking advantage of a single treatment‐inducing plant protection and stimulation (see **Figures** **11** and **12**). In particular, we elucidated that foliar‐spring of this material at such relatively low concentrations could significantly enhance the production of transient ROS, which is a hallmark to possibly activate systemic acquired resistance (SAR) or other pivotal defense/growth‐related mechanisms like induced systemic resistance (ISR), contributing to simultaneously prime the plants and boost their signaling pathways likely without a significant reliance or in combination with agrochemical applications.

Based on these findings and our molecular bioassays, phytohormone measurements, and transcriptomic analyses of different plant models (reverse transcription‐quantitative polymerase chain reaction and RNA‐sequencing and associated Kyoto encyclopedia of genes and genomes, including plant‐pathogen interaction, proteasome, and mitogen‐activated protein kinase signaling, ribosome biogenesis in eukaryotes, and photosynthesis‐antenna proteins pathways, we concluded that this protection‐by‐nano‐design could efficiently prime the plants for enhanced quick response upon exposure to stress, which can be remaining in the memory of plant for future stresses. Treating plant systems with surface‐modified MXene likely promotes their overall growth processes at various developmental stages and improves their responsiveness and adaptation to future stresses.

MXene‐based nanomaterials are also known for their unique surface charge and electronic properties, making them negatively charged at different pH levels, ranging from around 3 to 10. In the case of MBenes, it is reported that they possess mixed‐charge properties as the boron induces partial positive charges. At the same time, the nitrogen atoms in the structure of MBenes have been identified as relatively negatively charged surfaces. Based on these principles and also our previous reports on Zeta poetical measurements of MXene‐based materials and derived heterostructures, we proposed possible mechanisms of how Ti_3_C_2_T_x_ likely interacts with phytopathogens through either surface electrostatic attachments or weak hydrogen bonding, leading to a significant distribution into their physiological/biological activities to ultimately reduce their proliferation and minimize infection symptoms both in vitro and in planta.^[^ ^65^ ^]^

**Figure 13:**
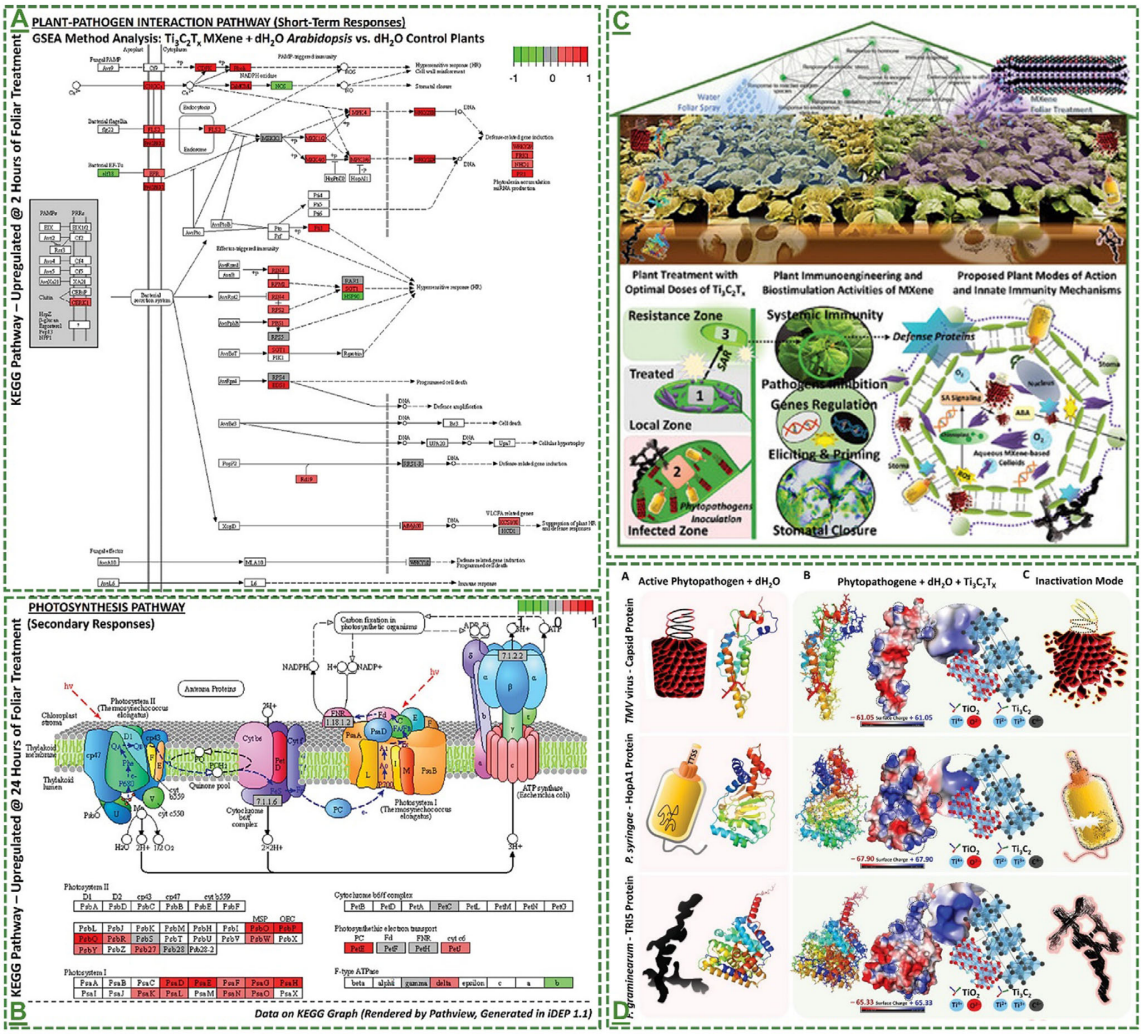
A–D) The pathway illustration and schematic of overall plant nano‐immunoengineering and biostimulant impacts of surface‐modified Ti_3_C_2_T_x_ on in planta short‐term and secondary RNA sequencing expressions, seed germination enhancement, in situ ROS production, stomatal closure‐inducing, and proposed antimicrobial modes of action mechanisms. It depicts the biocompatible behavior of these aqueous colloidal dispersions with plant systems, along with early phytopathogen interaction and longer‐term response of photosynthesis pathways. Representation of the proposed interaction of MXene with distinct plant pathogens using *PyMOL* software to generate standard protein structures of specific representative plant pathogens, including PDB IDs 6I5A, 4RSX, and UniProt ID: Q00909. The 3D crystal structures of the MXene (Ti_3_C_2_ Hexagonal *P6_3/mmc*, 194mp‐1094034) and surface titanium oxide (TiO_2_, Tetragonal *I4_1/amd*, 141mp‐390) were represented from “*Materials Project*” online open‐access database (https://materialsproject.org) with reference to the publication associated under the user agreement of accepting “Creative Commons Attribution license 4.0”. A–D) Reproduced with permission.^[^ ^65^, ^108^ ^]^ Copyright 2024, Wiley‐VCH GmbH.

In particular, applying foliar‐spraying of the negatively surface‐charged MXene dispersions likely induces physicochemical/molecular interactions between the nanomaterial and different plant pathogens, including viruses, bacteria, and fungi types. Due to the enrichment of MXene's surfaces with an abundance of negatively‐charged bioactive functional groups, these materials possess the intrinsic capability of attaching directly/indirectly with the positively charged subsites or surface proteins of the pathogens for creating temporary nano‐micro interactions, mitigating the cellular functions of host pathogenic organisms, and hindering their survival/growth (**Figure** **13**). Even though, the proposed MXene‐pathogen interactions are in their early stages and must be expanded by further biological experiments and detailed mechanistic bioinformatic studies; our novel findings have set paradigms for further research investigations in different plant systems and actual crops to be considered in future work to optimize and extend the current understanding of plant‐MXene interactions when MXene and potentially MBenes interact with phytopathogens, as well as detailed studies on the impact of these multifunctional materials on plants and their nano‐biocompatibility to the surrounding ecosystem.^[^ ^65^, ^108^ ^]^

In summary, our reports on plant immunoengineering and biostimulation impacts of Ti_3_C_3_T_x_ MXene‐based aqueous colloidal dispersions have demonstrated strong potential by triggering a sophisticated eliciting/priming mechanism to enhance resilience and systemic immunity under multiple stress conditions. At low doses, foliar application of colloidal MXene induced beneficial ROS production, leading to antiviral, antibacterial, and antifungal effects through direct pathogen‐interaction and systemically activation of plant defense pathways – particularly JA‐related ISR signaling. These multifunctional, low‐toxicity nano‐based biostimulants suggest a promising platform for next‐generation plant vaccines and biostimulants.

### 3.5 Reported Application(s) for Improved Cotton Plant Tolerance to *Verticillium dahliae* fungi

Nanomaterials and Nano‐based substances offer a promising alternative to conventional antifungal agents due to their tunable physicochemical properties, high surface area, and small size, which enables them to penetrate fungal cells and inhibit fungal growth and development through cell membrane disruption, inhibition of essential enzyme activities, and interference with DNA replication and transcription processes. In this regard, MXene and MBene can provide targeted delivery, controlled release, and reduced development of pathogen resistance, while minimizing environmental persistence and non‐target toxicity.

Building on the reported intrinsic in vitro anti‐phytofungal activity of MXene‐based materials, an innovative study by Qiu et al. reported the in planta impact of polyethyleneimine‐surface‐coated Ti_3_C_2_T_x_ MXene quantum dots composite on enhancing the tolerance of living cotton plants against *Verticillium dahliae*, which can cause significant wilt diseases in this crop.^[^ ^102^ ^]^ This is one of the major cotton plant diseases globally, and ongoing efforts have been made to improve their production and reduce the severity of this phytofungal infection. Following the design, fabrication, surface modification, and characterization of this composition, the authors studied its intrinsic capability to maintain ROS homeostasis and underlying mechanisms in this plant type. By focusing on ROS management, they conducted extensive experiments to understand the possible biological and biochemical mechanisms behind this bioactivity at different time points post‐treatment.

**Figure 14:**
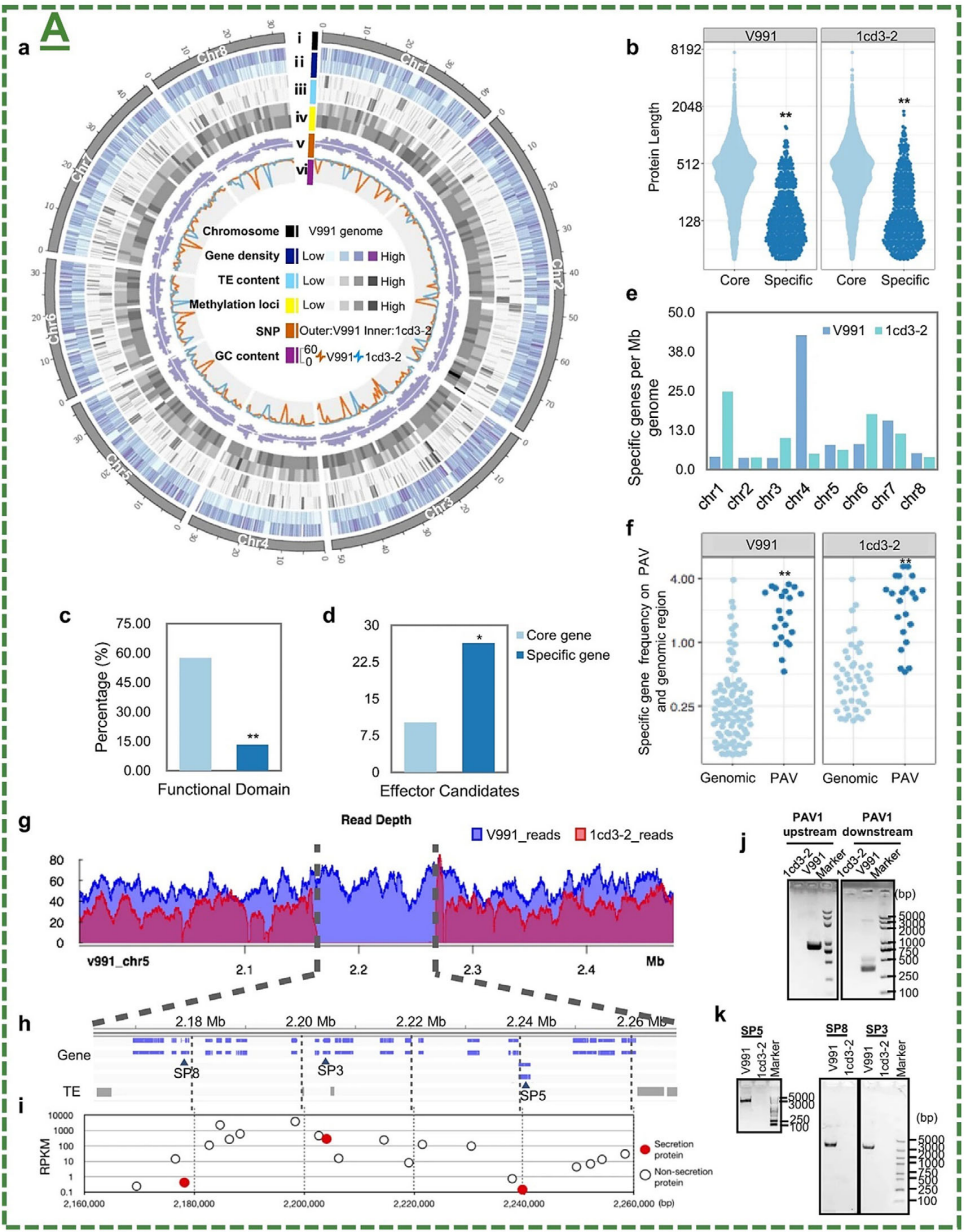
A) Graphical data represented on comparative genomic analysis, depicting the presence and absence of variations of two distinct fungal isolates. a) An overview of the chromosomal features of two stages in cotton (V991 and 1cd3‐2) transcriptomes referred to its original publication for genetic and epigenetic data. In particular, V991chromosomes and gene density (bin: was reported 10 kb) are depicted. The outer and inner encompass V991 and 1cd3‐2, respectively. The transposable element contents and methylation loci (including 6 mA and 4 mC) are reported with bins of 10 kb and 100 kb, respectively. The distribution of single‐nucleotide polymorphisms (bin is reported as 100 kb). The guanine‐cytosine (GC) content (bin is reported as 100 kb). The red and blue lines are related to V991 and 1cd3‐2, respectively. b) Specific genes encoded smaller‐size proteins with a *p*‐value lower than 2.2e‐16 (Wilcox test). c) Specific genes were reported with a lower possibility of encoding the proteins with functional domains, owning at least one defined Gene Ontology annotation. d) The statistical analysis performed was Student's *t*‐test (**p < 0.01). e,f) The distribution of specific genes in each chromosome (regions with a higher frequency of specific genes, other than the regions of random genomics (Wilcox test). g–i) The author has mentioned in this data that on chromosome 5, a large insertion incident affected several specific genes, including twenty genes (three encoded secreted SP3, SP5, and SP8 proteins). The maximum gene regulation levels of each gene, from the upper panels are also represented. j,k) The PCR confirmation of these regions and specific SP3/SP5/SP8 genes is reported. It is noted that this is a triplicate experiment with high robustness and accuracy of each result (the file source data is presented in the original paper. (A): Adapted with permission.^[^ ^102^ ^]^ Copyright 2023, Springer Nature.

**Figure 15:**
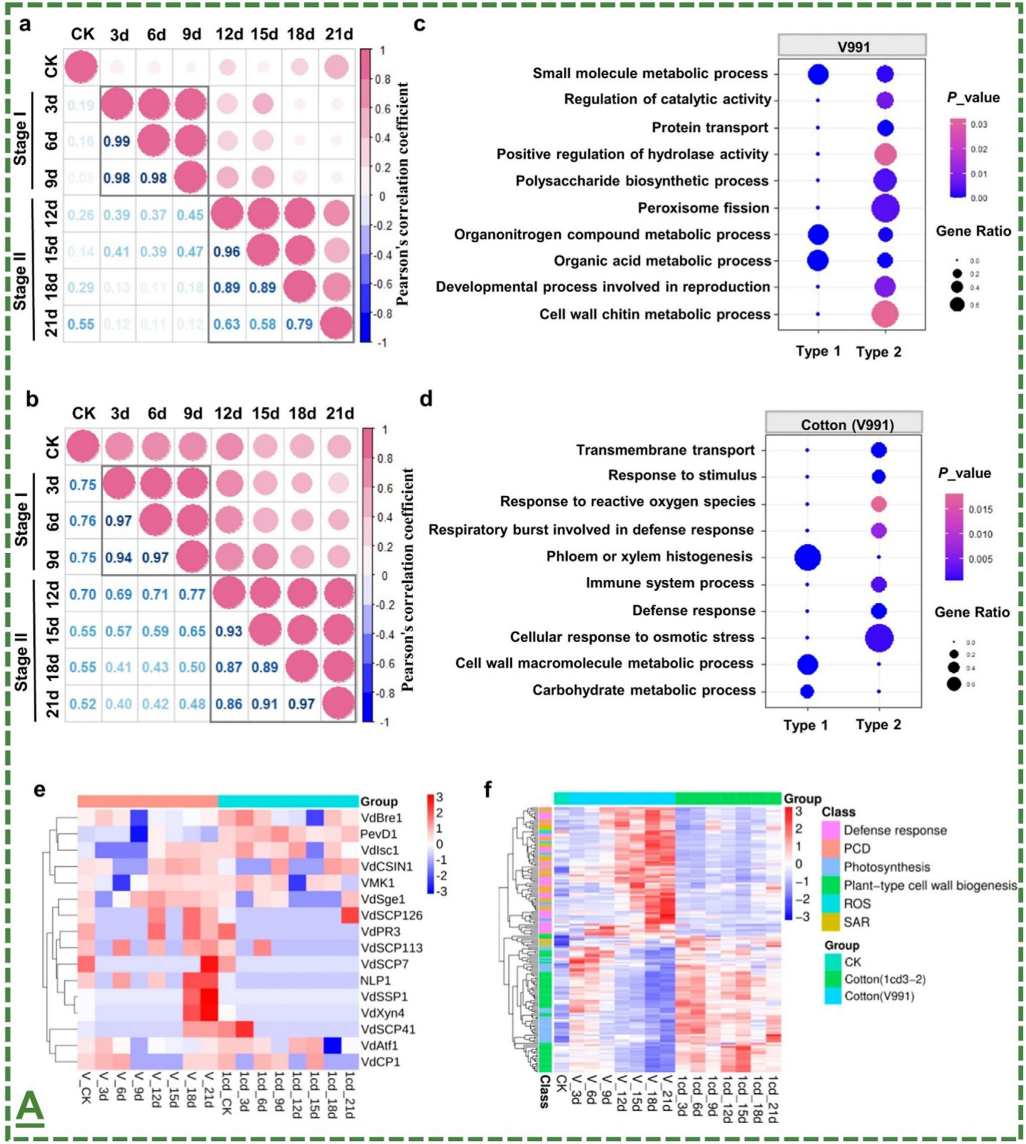
A) This represented figure revealed in planta transcriptome analysis of the interaction of cotton plants with *Verticillium dahliae*. a) The correlation analysis of the FPKM (Fragments Per Kilobase of exon per Million mapped reads) for differentially‐expressed genes in the V991 transcriptome at different time points ranged from 3 to 21 post‐infection, including the Pearson correlation of the FPKM to thoroughly define the similarity of obtained gene expression patterns at these time points. b) Their data of values and colors are indicated by Pearson's correlation coefficients, correlation analysis for differentially‐expressed genes in V991 transcriptome between different time points from day 3 to day 21 post‐infection. c) This figure also represented the observed GO enrichment analysis in response to differentially‐expressed genes in the V991 transcriptome (those that were upregulated at stage i are labeled as “type‐1”; and those that upregulated at stage ii are coded “type‐2”. d) In this figure, the authors have mentioned that the color key corresponds to *p* values for each enrichment score, determined by “Fisher's exact test”. e,f) Further, the clustering heatmap of the reported virulence‐related genes in V991 and 1cd3‐2 transcriptomes are represented with a reference to their Source data file. (A): Adapted with permission.^[^ ^102^ ^]^ Copyright 2023, Springer Nature.

**Figure 16:**
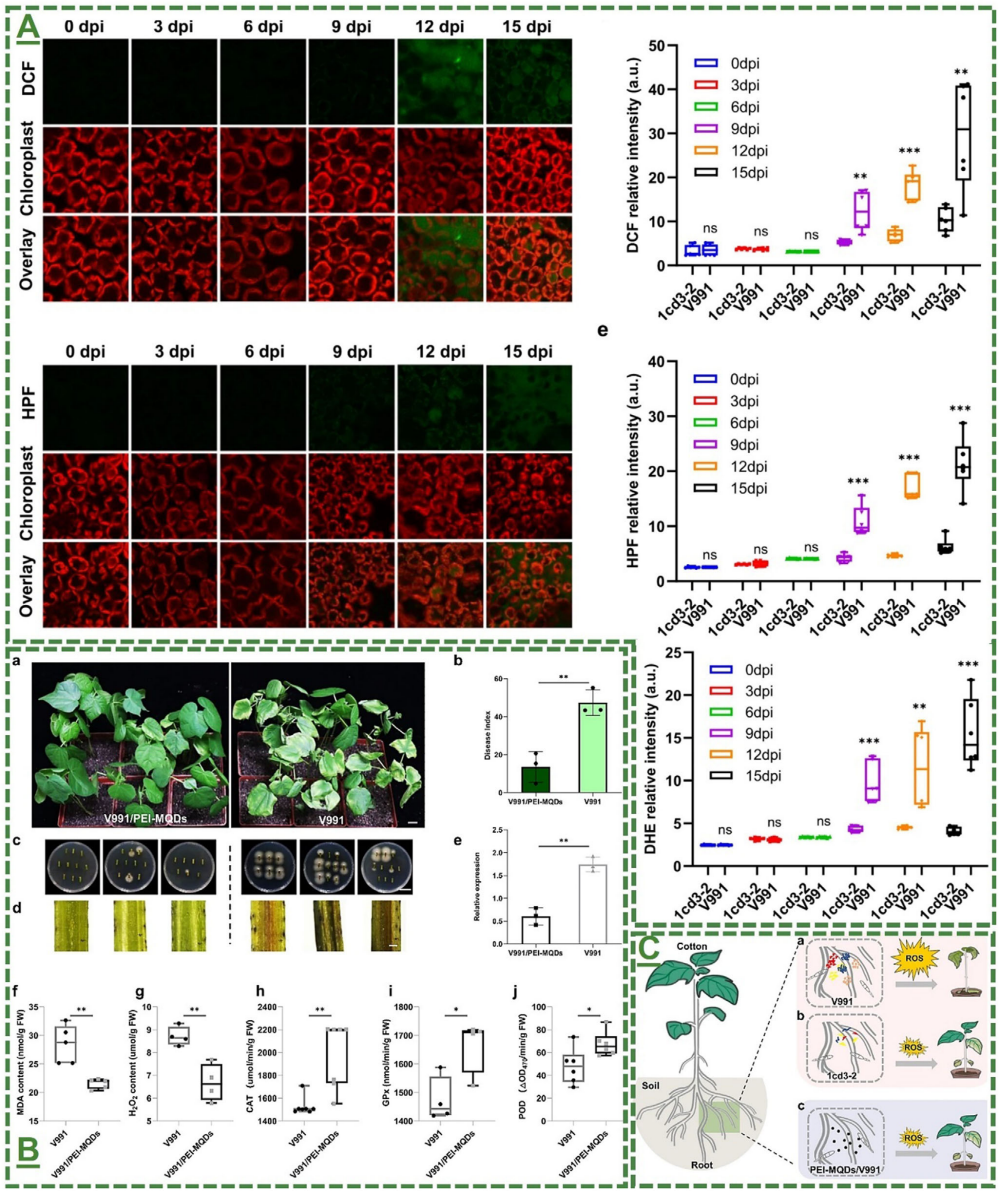
A) The dynamics of ROS production in cotton affected with *Verticillium dahliae* at different time points, including confocal microscopy images of hydrogen peroxide dichlorofluorescein and hydroxyl radicals hydroxyphenyl fluorescein in infested cotton leaves with V991 at different times (scale bar is reported as 50 µm). The quantitative analysis of the fluorescence intensity of leaf hydrogen peroxide, hydroxyl radicals, and superoxide anion dihydroethidium in cotton affected with V991 and 1cd3‐2 at different time points (by Student's *t*‐test: “ns”: not significant”; “**”: *p *< 0.01, and “***”*p *< 0.001,) with a reference to their Source data file. B) The impact of the s‐designed MXene‐based quantum dots on improving cotton tolerance to *Verticillium dahliae* by scavenging and miniating ROS homeostasis. The disease symptoms of these plants at day 18 post V991 inoculation with and without MXene treatment (scale bar: 2 cm). Further, the disease index of the infected plants throughout the performed fungal recovery bioassay, representing the sections from the cotyledonary node of plants at day 18 of infection (scale bar: 100 µm). qPCR analysis showed the amount of fungal DNA content analyses. Their data indicate that treating the cotton with these modified MXene quantum dots could effectively reduce malondialdehyde and hydrogen peroxide levels with additional catalytic activities compared to the control plants. C) The proposed interaction model between *Verticillium dahliae* and cotton plants in terms of regulating V991 expression of more virulence‐attributed genes during this interaction, which could cause excessive ROS accumulation in the cotton plants with severe impacts. In the case of 1cd3‐2, fewer virulence‐related genes were expressed during this host interaction. As of result, a lower accumulation of ROS was observed, and when treated with these MXene‐base quantum dots, an increased resistance to V991 was reported for the cotton plants through scavenging ROS homeostasis mechanisms. A–C) Reproduced with permission.^[^ ^102^ ^]^ Copyright 2023, Springer Nature.

Moreover, the authors accordingly examined key parameters of plant health, including fungal infection/disease severity, biomass accumulation, chlorophyll content, and the overall growth of the MXene‐treated plants in response to pathogen exposure and in a comparable mode with control groups. The impact of these MXene quantum dots has been further evaluated based on their capability to reduce the symptoms corresponding to pathogen‐induced wilting and to promote healthy growth as compared with MXene‐free control plants. In particular, the genomes of two *Verticillium dahliae* (the isolate with more virulence defoliating V991 and the nondefoliating one 1cd3‐2) were assembled and studied by detailed transcriptome and comparative genomics Analyses. As represented in **Figures** **14**, **15**, **16**, their data showed that the genes associated with these fungal pathogen virulences were mostly induced at the late stages of *Verticillium dahliae* infection (known as stage‐II), accompanied by a remarkable burst of ROS and upregulation of defense‐related genes in this plant. They have also reported the identification of V991‐specific virulence gene *SP3* with a high level of expression affected by these MXene quantum dots during this specific stage of the fungal infection, Stage‐II.^[^ ^102^ ^]^

Furthermore, their findings have suggested that the *Verticillium dahliae SP3* knock‐out strain appeared with a weakened virulence, triggering lower amounts of ROS production in tested plants. To overcome this challenge and control the *Verticillium dahliae*‐corresponded disease, the surface of Ti_3_C_2_T_x_ MXene quantum dots has been further activated by polyethyleneimine‐based coating layers, which have intrinsically possessed the higher capability for removing ROS in cotton plants. Additionally, their result suggested that treating the cotton seedlings with these modified quantum dots could effectively maintain ROS homeostasis with enhanced biocatalytic activities (peroxidase, catalase, or glutathione peroxidase), contributing to becoming resistant to *Verticillium dahliae* and related infections through possible mechanisms such as improved cell wall interaction, pattern recognition, and/or extracellular enzyme processes.^[^ ^102^ ^]^

Moreover, the authors described how the comparative genomics of presence and absence variations are correlated with these virulence genes. They revealed that on chromosome 5, which was the most induced gene, is “v991_EVM0005595 (Nmr2)”, the related NADPH‐dependent genetic part is possibly switched to regulate infection symptoms in cotton plants. They have further described that the NMR‐related transcriptional corepressor can contribute to activating/regulating the expression of a set of virulence‐associated genes in other plants, such as rice, during appressorium‐mediated initiation of rice blast fungal‐based diseases. Their data also identified the upregulation of four specific secretion proteins on chromosome 7, suggesting that comparative genomics of presence and absence variants are influenced by these specific, distinctive variation‐containing genes, which can subsequently lead to divergence between the strains of this fungus. In addition, they evaluated an interaction between *Verticillium dahliae* and the cotton plant, proposing two related stages during this molecular interaction using time‐course transcriptome analysis.^[^ ^102^ ^]^

They also reported that “VdCP121”, which can play a key role in suppressing the host defense‐related responses, was induced during stage I. Also, their data suggested that some genes, such as VdAtf122 and VdBre123, which are related to the metabolic activities, were induced during stage I, contributing to providing nutrition to the colonization. On the contrary, the identification of genes related to microsclerotia and mycelial growth, including VdPR324 and VdSge125, VMK126, and VdCSIN127, was induced during stage II. Therefore, they speculated that high expression levels of SP3 in stage II could potentially promote the accumulation of more ROS, accelerating host death. Additionally, they found that several effectors, including VdSCP728, VdXyn430, and VdNLP129, were expressed during this stage II, which contributed to accelerating plant death. They concluded based on these data that *Verticillium dahliae* could transition from a biotrophic to a necrotrophic phase during this host‐pathogen interaction. They also highlighted the ROS‐scavenging impact of these quantum dots on maintaining ROS homeostasis in infected cotton plants to protect their cotton from *Verticillium dahliae* and associated infections (Figures 14, 15, 16). The authors suggested to study that how these stages are transformed, including detailed multi‐omics analysis, as future work.^[^ ^102^ ^]^ In summary, In this study, PEI‐MQDs were applied to cotton seedlings, effectively scavenging excess ROS and accelerating antioxidant enzyme activities, enhancing plant tolerance to fungi *Verticillium dahliae*. This innovative study uncovers a new ROS‐mediated virulence mechanism and suggests PEI‐MQDs as a promising agent for disease management in cotton plants.

### 3.6 Reported Application(s) for Enhanced Scavenging ROS for Promotion of Seed Germination

Nanomaterials have demonstrated potential in promoting seed germination by enhancing water uptake, enhancing nutrient accessibility to seeds, stimulating key enzyme activities, and regulating phytohormone levels. Their nanoscale dimension enables them to facilitate penetration through seed coats, accelerating metabolic processes and ensuring uniform germination, which is an essential factor for early seedling vigor and optimal crop productivity under stress conditions. Salinity‐induced oxidative stress is a major challenge in crop productivity and sustainability. MXene materials have shown promise in enhancing plant tolerance or resilience to salt stress.

**Figure 17:**
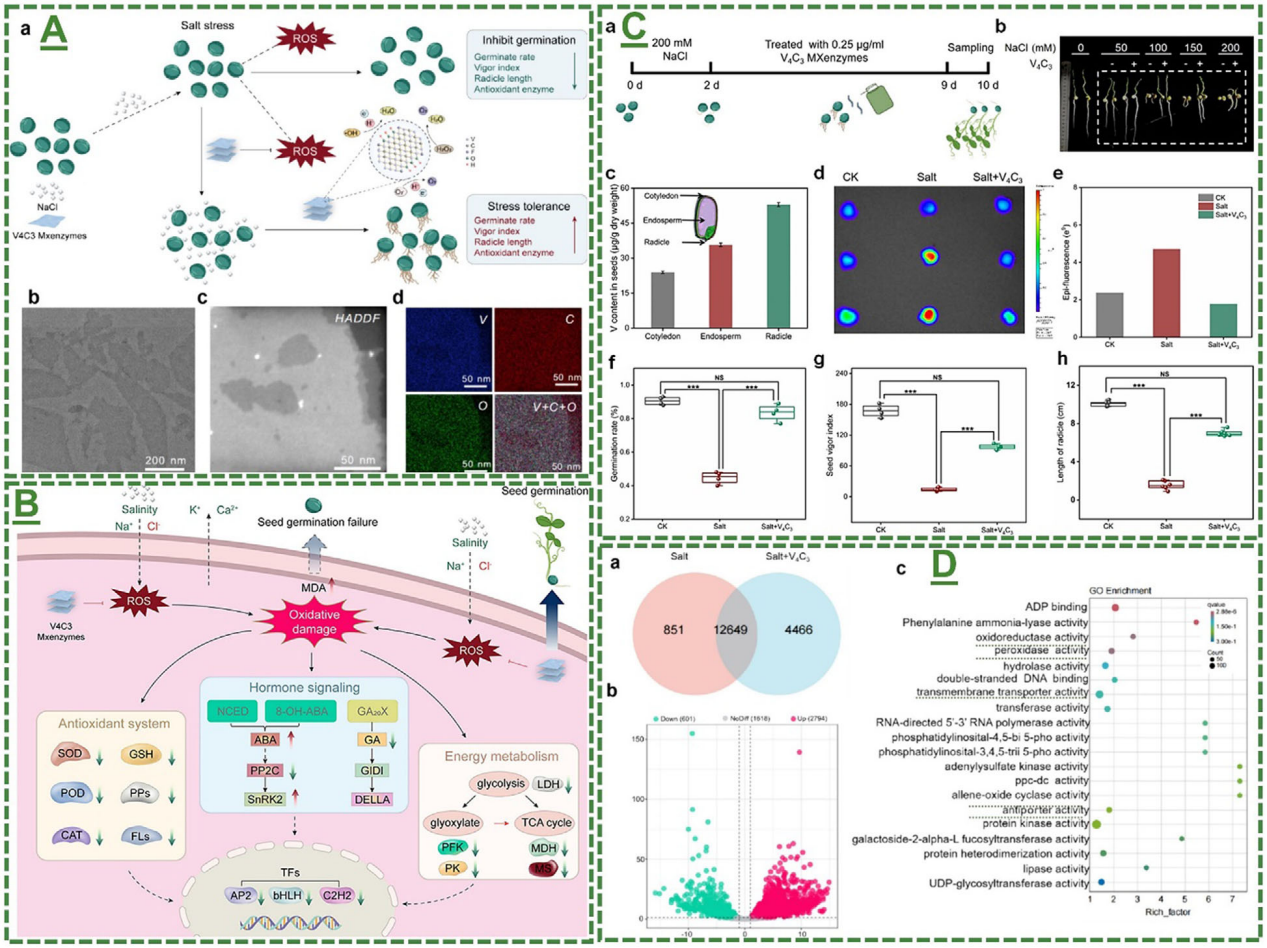
A–D) The schematic illustration of an overall seed germination enhancement capacity of vanadium carbide‐based MXene nanosheets (V_4_C_3_) and their physicochemical characterization in prepared aqueous dispersions, including their morphology and elemental chemical composition of the prepared sample by electron microscopy and EDS mapping. The proposed mechanisms of function and experimental validation in different bioassays on the impact of these nanosheets on the tested model. These particular data represent the role of this material in inducing/enhancing ROS productions and seed germination process under salt stresses for more effective and efficient maturation towards agricultural application and future crop production strategies that rely on nanotechnology. The represented figure also covers the reported multi‐omics analyses of this material on promoting salt‐stressed seed germination through scavenging ROS regulation bioactivity. A–D) Reproduced with permission.^[^ ^103^ ^]^ Copyright 2024, American Chemical Society.

In this regard, elsewhere, another study has reported the preparation and impact of vanadium carbide‐based MXene (V_4_C_3_) as a next‐generation nanozyme (MXenzyme) with biocompatibility and ROS scavenging bioactivity properties to ameliorate the salt stress‐induced inhibition of seed germination in *Pisum sativum* (pea).^[^ ^103^ ^]^ Their primary goal was to evaluate the application of this MXenzyme to relieve oxidative stress induced by salinity and restore antioxidant bioactivity in these seeds, thereby effectively improving their phenotypic traits during the seed germination process. Throughout antioxidant assays and molecular mechanisms, the authors optimized the bioactivity impact of the material. In particular, using physiological and biochemical analyses, they reported that the optimal treatment condition was observed when salt‐subjected seeds at 200 mM were treated with V_4_C_3_ MXene at a concentration of 0.25 µg mL^−1^. As represented in **Figure** **17**, their results indicate that the ROS levels in as‐stressed seeds were significantly increased by around 1.5 times compared to the samples in the control groups. However, treating them with this MXene‐based nanozyme could remarkably reduce the ROS levels within these seeds, contributing to bringing them back to approximately normal levels. Besides, their assessment of phenotypic parameters in these seeds showed that the germination rate, radicle length, and vigor could be improved by around 2, 3.5, and 5.5 times, respectively, after treating with as‐prepared MXene. They concluded by stating the protective impact of V_4_C_3_‐based MXene in mitigating the harmful effects of salt stresses on the germination process of pea seeds.^[^ ^103^ ^]^

Furthermore, the authors commented on the positive impact of this material on restoring the vigor of pea seeds for improved overall biomass. In particular, their transcriptomic data suggested that this nanozyme treatment might start the activation of specific gene regulations in these plants compared to control groups, which may increase the ability of plants to better cope with salinity stress, while the identified gene expressions of V_4_C_3_ MXene‐treated seeds was found to be closer to those of the control plants, indicating the potential effect of the material to relieve the induced damages to plant's growth by salt stress. Moreover, their data showed a significant down‐regulation (∼90%) in the expression of key genes related to lipoxygenase activity in pea seeds, while a remarkable upregulation was identified in the level of MaTE, TpTF, PT‐STKS genes, and EATT family compared to the control groups, suggesting high activities of protein kinases, transmembrane transporter, and antiporter to promote seed tolerance to salinity‐induced damages.

Moreover, they discussed that the associated upregulated genes in V_4_C_3_ groups might also contribute to restoring the related cycles between glutamine and oxidized glutathione, as well as to regulate the glutathione‐related metabolism pathways, modulating the redox status in the seeds. They also reported a notable down‐regulation (∼78%) in the expression of the pivotal enzymes related to abscisic acid‐biosynthesis9‐cis‐epoxycarotenoid dioxygenase (ABA‐NCED) in salt‐treated seeds. This significant downregulation is described as the induced salinity stress may remarkably promote the accumulation of ABA‐based synthesis responses. However, V_4_C_3_‐treated seeds showed a reversed trend with upregulated NCED expression by ∼91% (see Figure 17). Their study has opened up new avenues of study for multi‐omics‐based investigations on the bioactive impacts of V_4_C_3_‐based nanozymes on different seed and seedling systems and underlying mechanisms in a comparable manner with other MXenes and probably bioactive MBenes.^[^ ^103^ ^]^

In summary, this study reported the bioactivity of V_4_C_3_ MXene‐based nanozyme (MXenzyme) for enhancing pea seeds germination rate under salinity stress. Transcriptomic and metabolomic analyses demonstrated that this MXenzyme treatment modulated antioxidant‐related genes, phytohormone signaling, and energy metabolism, thereby stabilizing redox homeostasis. The induced‐V_4_C_3_ ROS‐scavenging activity plays a key role in enhancing plant growth under salinity.

### 3.7 Reported Application(s) for Enhanced Tolerance of *Torreya grandis* to Lead (Pb) Stress in Soil and Antifungal Impact on Plant Root Rot Disease

**Figure 18:**
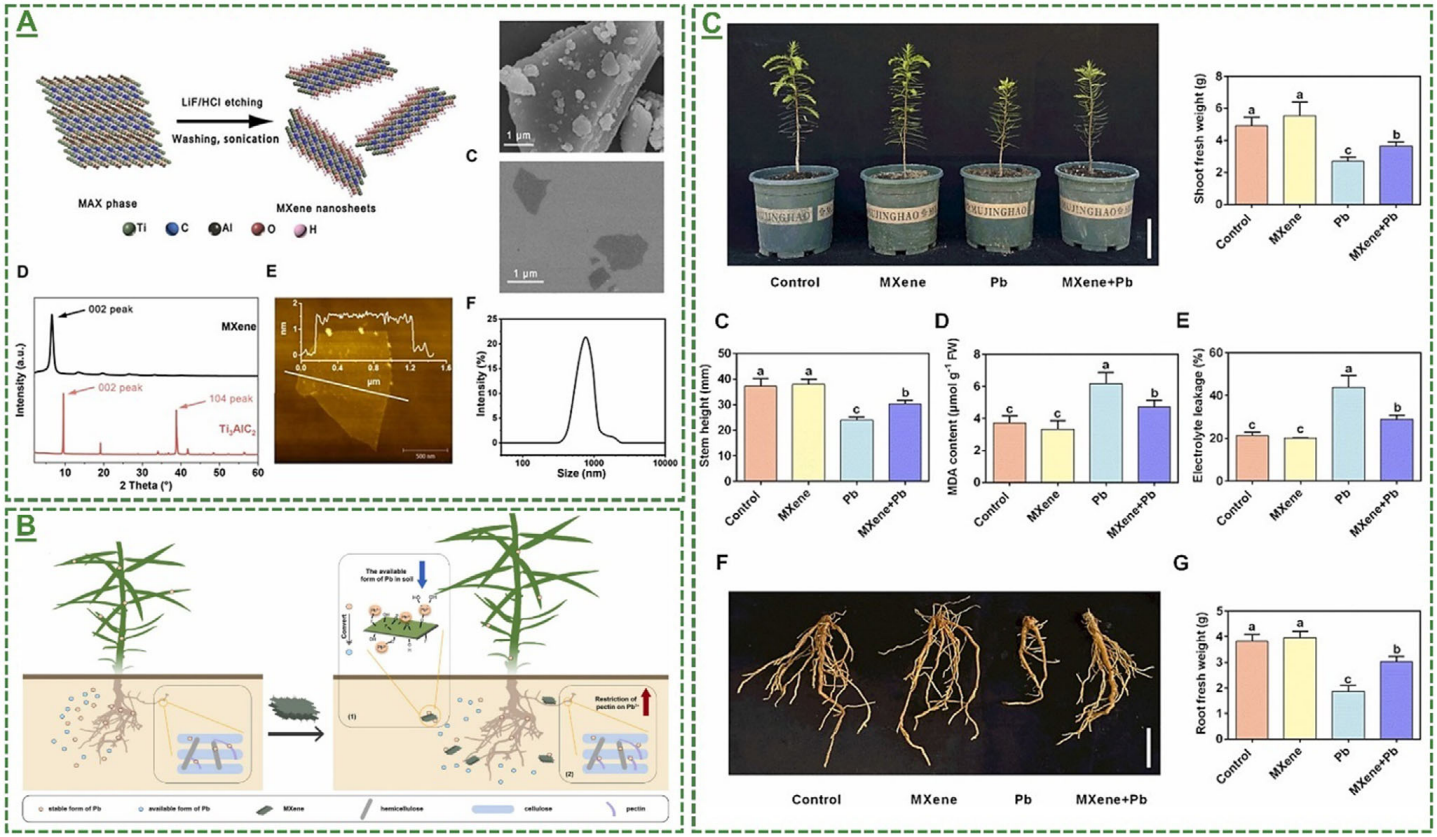
A–C) The schematic illustration of a typical synthesis of MXene nanosheets from MAX phase and the microstructure characterization of the obtained Ti_3_C_2_T_x_ MXene nanosheets for enhancing the tolerance of *Torreya grandis* to Pb stress in soil. The effect of MXene on enhancing the phenotype, shoot fresh weight, and root fresh wright of the *Torreya grandis* after Pb/MXene treatment. According to their bioassay, one‐year‐aged *Torreya grandis* seedlings were mixed with or without Lead nitrate (Pb(NO_3_)_2_, 2.8 g) or Ti_3_C_2_T_x_ MXene (200 mg) in each pot for 45 days of treatment. (the scale bars are 10 cm and 5 cm, by order, analyzed by one‐way analysis of variance (ANOVA), *p* < 0.05). A–D) Reproduced with permission.^[^ ^104^ ^]^ Copyright 2023, Elsevier B.V.

Nanomaterials have been utilized to improve plant resilience to heavy metals in contaminated soils. Their mechanism is through binding and immobilizing heavy metals, ion exchange, and/or precipitation, thereby minimizing the metal bioavailability and eventually uptake by roots. Additionally, they can stimulate antioxidant activity, modulate metal transporters, and sustain nutrient homeostasis, reducing metal‐induced ROS damage and enhancing normal plant growth under stress conditions. In this regard, elsewhere, in a recent study, a novel impact of Ti_3_C_2_T_x_ MXene on significantly enhancing the tolerance of *Torreya grandis* to Lead (Pb) stress by reducing its accumulation in soil was studied for future application in agriculture and forestry.^[^ ^104^ ^]^ In particular, the authors have assessed the phenotypic and physiological analyses, and proposed two independent mechanisms of Ti_3_C_2_T_x_ nanosheets by converting the naturally‐available form of Pb into its more stable forms via their strong adsorption and surface interactions, resulting in a decreased availability of Pb in specific forms in the soil system. The second mechanism has been proposed through the impact of MXene on increasing the cell wall pectin content to restrict more Pb in cell walls by regulating the expression of pectin synthesis/metabolism‐related genes, including TgPLL2, TgPG5, TgPG30, TgPLL11, TgGAUT12, and TgGAUT3 in the roots of *Torreya grandis* plants (see **Figure** **18**A–D). These novel findings further highlight the multi‐functionality of Ti_3_C_2_T_x_ for future agricultural applications.

**Figure 19:**
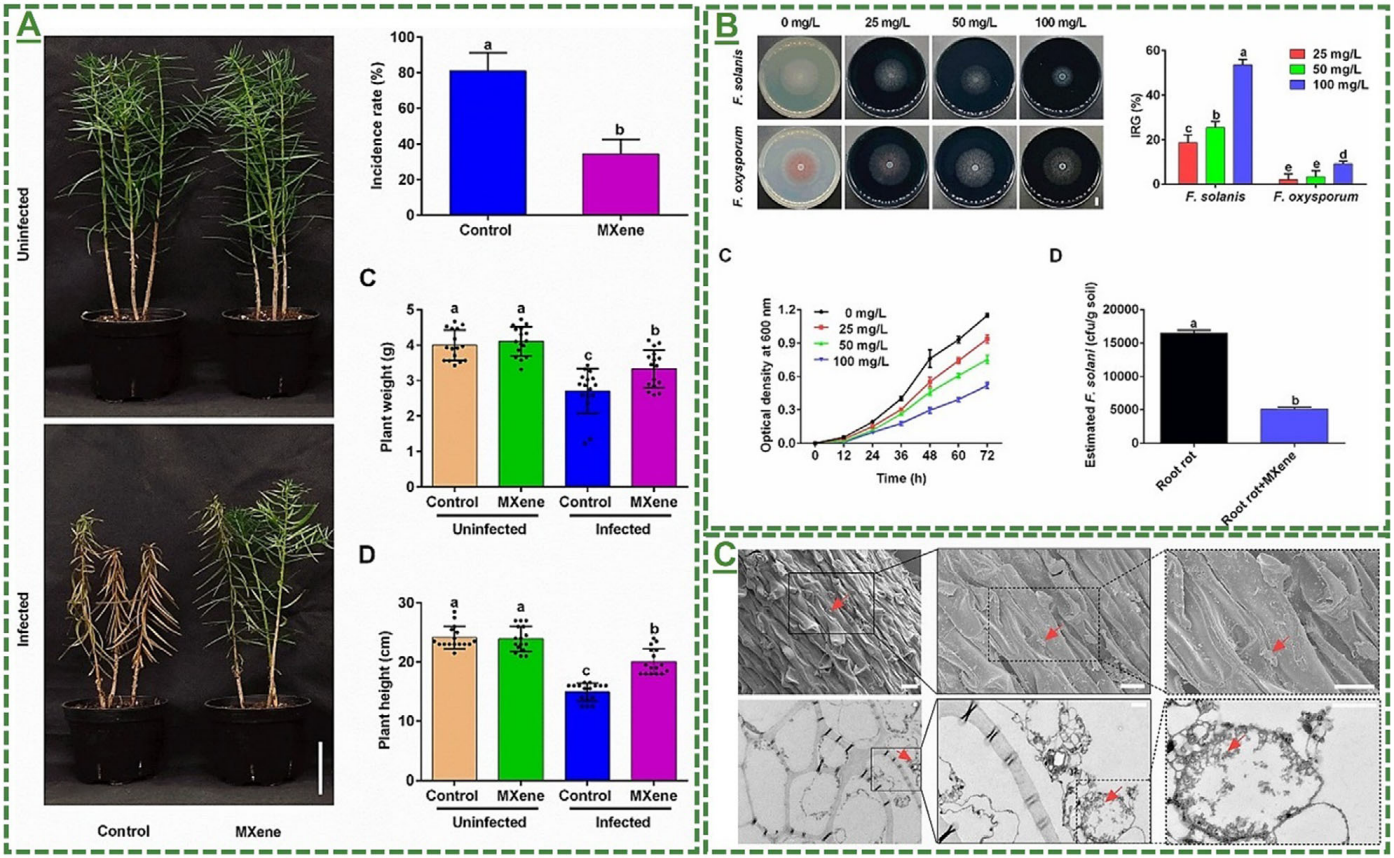
A–C) The preparation route and morphology assessment of these nanosheets towards application in *T. grandis* protection against root rot disease. These data represent electron/optical microscopy images and depict the impact of MXene on reducing plant infection and improving plant tissue illustration in the tested model. A–C) Reproduced with permission.^[^ ^105^ ^]^ Copyright 2024, Elsevier B.V.

Moreover, in another recent work by the same research group, an innovative impact of Ti_3_C_2_T_x_ on protecting *Torreya grandis*, a tree species susceptible to root rot caused by pathogenic fungi infection, is reported.^[^ ^105^ ^]^ The authors reported that treating this specific plant with this MXene could effectively enhance application in soil against *Fusarium* genus activity, as stated a significant reduction of over 68% through possible cell membrane damaging mechanisms, causing the disintegration and cell death of *Fusarium solani* (see **Figure** **19**). In that study, high‐throughput sequencing was used to compare microbial communities of root‐rot‐infected soil in the presence of MXene. The principal coordinates analysis and analysis of similarities (PCoA/ANOSIM) analyses indicated that treating with this MXene could enhance the abundance of fungi like *Aspergillus*, *Trichoderma*, and *Botrytis*, and decrease the abundance of *Actinomucor*, *Mucor*, and *Rhodosporidiobolus*. These functional predictions showed that plant pathogenic fungi were significantly suppressed in MXene‐treated soil, suggesting that MXene reduces soil‐borne pathogens by altering the diversity and function of the microbial community. Their results also indicate that treating the plants with 100 mg MXene could effectively enhance the shoot and root fresh weight in the pathogen‐inoculated plants compared to the control group (see Figure 19).

To confirm the direct effect of this MXene on the mycelial radial growth of *Fusarium*, an in vitro plate assay was performed with different MXene concentrations (0, 25, 50, 100 mg mL^−1^). The results of this study revealed that MXene solution inhibited the mycelial radial growth of *F. solani* in a dose‐dependent manner, while *F. oxysporum* revealed slight inhibition at higher MXene concentrations. In this analysis, the lower inhibitory effect of TiO_2_ (the oxidation form of MXene) indicates the intrinsic antifungal activity of MXene, which is not due to its oxidation product. The observed color changes in the mycelium of *F. oxysporum* in the presence of MXene could be due to pigment production under stress conditions. Interestingly, this effect was not significantly visible on *F. solani* mycelium, suggesting different inhibition modes of action between the tested fungi.

The morphology analysis of *F. solani* using SEM and propidium iodide staining indicated that MXene damaged the cell membranes of *this* fungi, causing abnormal and distorted mycelium, leading to increased permeability and cell death. This tracking observation was further supported by enhanced extracellular conductivity and protein content, showing leakage of cytoplasmic contents and cell structure disruption (see Figure 19). Further, these MXene flakes have been shown to be transported into roots through *Torreya grandis* root air spaces, resulting in lignin accumulation in the roots of these plants through enhancing the expression and activities of lignin biosynthesis‐related genes for improved plant antifungal applications.^[^ ^105^ ^]^

In summary, these two related studies reported the potential of Ti_3_C_2_T_x_ MXene as a versatile tool for sustainable forestry through enhancing *Torreya grandis* resistance to abiotic and biotic stresses. Under abiotic stress, MXene application significantly reduced Pb accumulation via dual mechanisms of adsorbing/stabilizing Pb in soil, and its immobilization by enhancing cell wall pectin content through regulation of pectin‐related gene expression. They suggested that MXene could inhibit *Fussarium solani* mycelial growth and enhance lignin accumulation in roots by regulating lignin biosynthesis‐related genes, promoting the defense mechanism against root rot disease.

### 3.8 Reported Application(s) for Improved Induce Plant Resistance to *Ralstonia solanacearum* via the Ethylene (ET)/Jasmonic Acid (JA) Signaling Pathway

**Figure 20:**
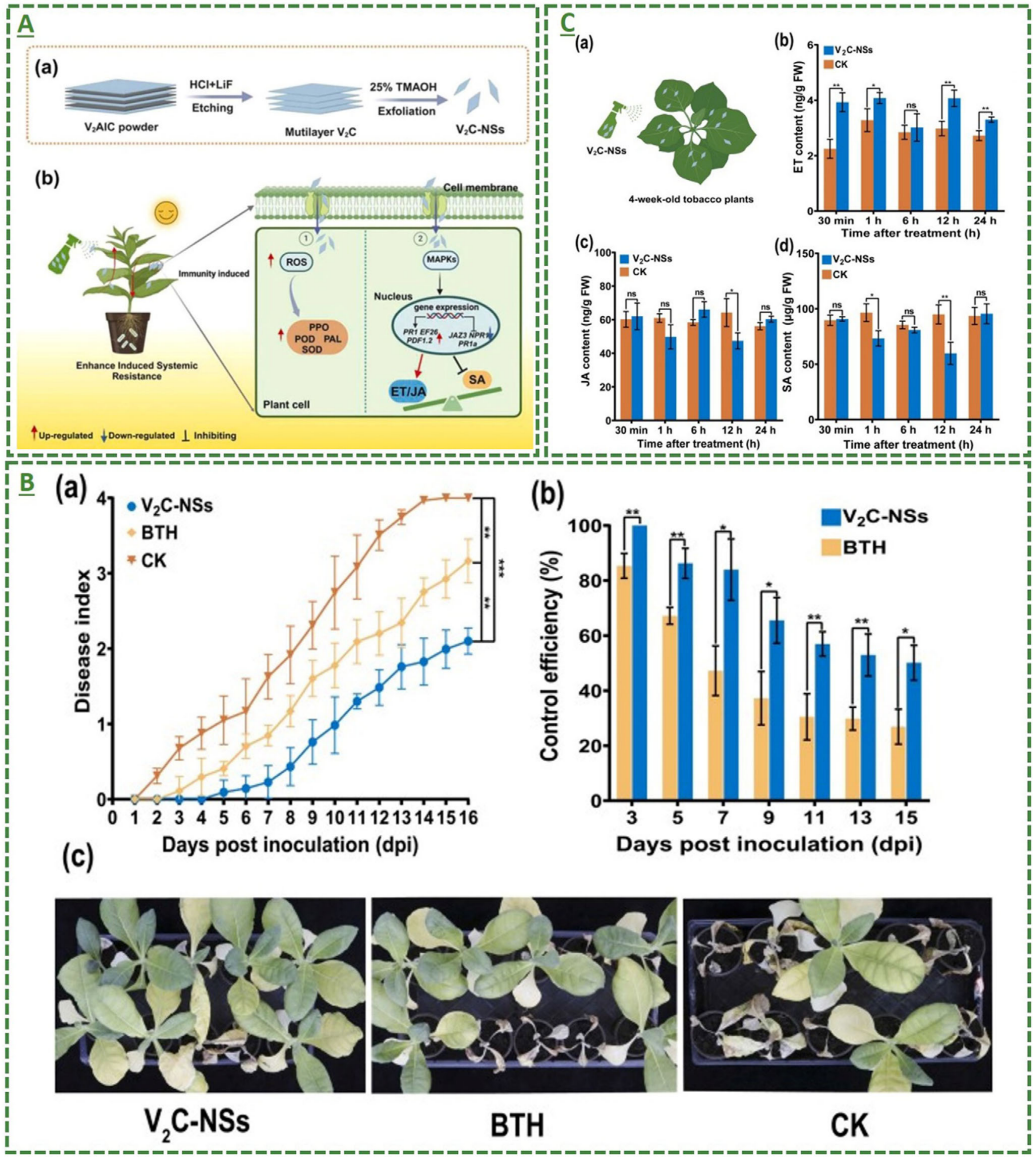
A–C) The schematic illustration of a typical synthesis and exfoliation process of converting vanadium aluminum carbide MAX phase to MXene nanosheets (V_2_CT_x_) using hydrochloric acid and lithium fluoride as the etchant and tetramethylammonium hydroxide for delamination and the targeted phyto‐application for inducing resistance to *Ralstonia solanacearum* via the ethylene/JA signaling pathway.The experimental validation and bioassays performed on the impact of these nanosheets on the tested model. In particular, this data encompasses the disease index and controlled efficiency bioassay and representative camera images of the plants before and after treatment, showing infection symptom in control. The ET/JA content measurements. A–C) Reproduced with permission.^[^ ^106^ ^]^ Copyright 2024, Elsevier B.V.

Bioactive nanomaterials have been shown to stimulate phytohormone signaling, particularly the ethylene/jasmonic acid (ET/JA) pathways, which are essential for plant defense responses. By regulating ET/JA signaling, these nano‐based substances promote the accumulation of protective secondary metabolites, improve structural barriers, and enhance resistance to necrotrophic pathogens. This fine‐tuned hormonal regulation enables effective plant defense priming while minimizing unnecessary metabolic burden. With rational aims of improving the environmental impacts of agrochemicals and conventional nanomaterials, a recent study has reported the preparation and potential application of vanadium carbide‐based MXene nanosheets (V_2_C) for direct effects on crop protection and inducing plant resistance to specific diseases.^[^ ^106^ ^]^ In particular, they reported the ability of three MXene nanosheets to promote growth in tested plant models and their underlying molecular mechanism for enhanced resistance to *Ralstonia solanacearum* (see **Figure** **20**). Their data suggested that these V_2_C MXene nanosheets could increase both forms of fresh and dry plant weight, promote ROS accumulation, activate specific defense enzymes, and upregulate ethylene and JA synthesis and expression of the responsive genes.

Through the MAPK signaling pathway, they showed that the bio‐synthesis of flavonoid and phenylpropanoid was also activated, while no remarkable changes occurred in the expression of the salicylic acid (SA) biosynthetic pathway. They concluded that these MXenes could effectively enhance plant resistance/resilience against *Ralstonia solanacearum* via enhancing ET/JA pathway‐related mechanisms. Further, their data suggested that foliar application of these MXene nanosheets could remarkably delay the occurrence of tobacco bacterial wilt, while the control reached over 65%, 55%, and 52% at day 9, 11, and 13 post‐inoculation, respectively. They also commented on the biocompatibility of these MXene nanosheets with tobacco seeds, bacteria, and earthworms, highlighting the plant and environmental compatibility of V_2_C‐based nanosheets.^[^ ^106^ ^]^

In summary, in this study, synthesized V_2_C‐based MXene nanosheets effectively induce *Nicotiana tabacum L* resistance against *Ralstonia solanacearum* via the ET/JA pathway. These nanosheets could effectively enhance ROS accumulation and activate defense‐related enzymes. Pre‐biocompatibility evaluations of this material suggest its potential application as an eco‐friendly plant resistance inducer for controlling tobacco bacterial wilt disease.

### 3.9 Reported Application(s) for Improved Interaction with Rhizosphere Bacteria in Tomato Plants

Tunable nanomaterials can modulate plant‐microbe interactions in the rhizosphere by influencing microbial community composition, promoting biofilm formation, and nutrient cycling, which contributes to nitrogen fixation regulation and essential phytohormone synthesis. Enhancing symbiosis interaction not only boosts nutrient uptake but also indirectly modulates plant defense, as many plant growth‐promoting rhizobacteria (PGPR) synthesize antimicrobial compounds and trigger systemic resistance against phytopathogens.

**Figure 21:**
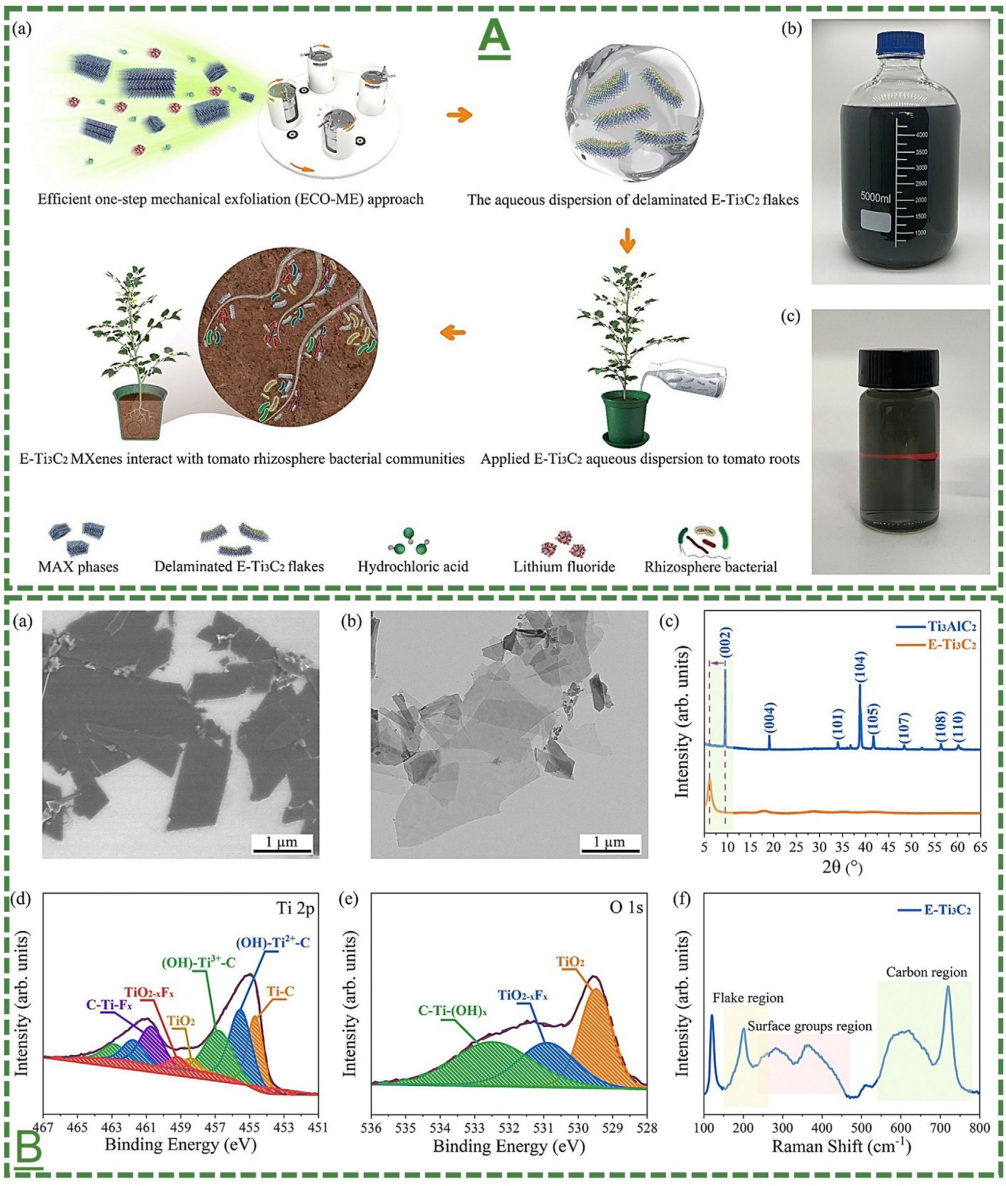
A) The schematic illustration of an efficient one‐step mechanical exfoliation process of converting titanium aluminum carbide MAX phase to Ti_3_C_2_T_x_ MXene nanosheets and their stock representation with surface optical transparency for inducing bioactivity to interact with bacterial communities in the tomato rhizosphere. B) The physicochemical characterizations of the prepared flake aqueous dispersions, including morphology, phase, surface chemistry, and functional groups validations of the prepared sample using SEM, TEM, XRD, XPS, and Raman spectroscopy. A,B) Reproduced with permission.^[^ ^107^ ^]^ Copyright 2024, Royal Society of Chemistry.

In this regard, elsewhere, a recent study by Liu et al. presented an innovative one‐pot strategy for mechanical exfoliation of Ti_3_C_2_T_x_‐based MXene sheets with relative antibacterial activity against rhizosphere bacteria colonizing in *Solanum lycopersicum* (tomato) plants.^[^ ^107^ ^]^ Rhizosphere bacteria colonization is crucial in farming this specific crop and for maintaining the ecological variations in the soil environments. Therefore, the authors studied the biocompatibility of MXene in tomato plants and the surrounding soil environments. In particular, the probable risk of nano‐interactions with these systems has been evaluated. Following the synthesis and characterization of these MXene nanosheets (**Figure** **21**), the authors assessed the influence of their material on rhizosphere bacterial populations/communities in tomato plants by high‐throughput sequencing. Interestingly, they observed no significant variations or adverse effects in the tested rhizosphere bacterial communities, even by directly applying around 10 mL of these Ti_3_C_2_T_x_‐based MXene dispersions at a concentration of 1200 µg mL^−1^ throughout a root irrigation method.^[^ ^107^ ^]^

Moreover, their results suggested that the slightly triggered fluctuation in the community of these bacteria that reverted to the initial level after around six days suggested a moderate biocompatibility of this material to this particular soil microorganism, with a negligible effect of MXene nano‐toxicity to the soil environment. This study suggested further investigations on MXenes' ecological effects and their biocompatibility improvements towards further development and optimization of MXenes and their soil interactions, aiming to increase the potential of MXenes and their improved versions for practical agro‐applications. This finding is relatively in line with the other reports in the literature suggesting the biocompatibility of MXenes and their modified formulations with different plants and other bio‐systems at controlled doses.^[^ ^107^ ^]^ In summary, this work reported an efficient one‐step mechanical exfoliation method for the rapid, economical production of Ti_3_C_2_T_x_ MXene nanosheets with enhanced hydrophilicity, extensive lateral dimensions, and rich surface functional groups. In tomato rhizosphere soils, it induced a low and temporary shift in the soil‐borne bacterial composition, suggesting the biocompatibility of this material for future agricultural applications, where negligible adverse effects on the functionality of soil microbial communities was showcased.

### 3.10 Reported Application(s) of Novel Chiral‐Engineered MXenes as Next‐Generation Plant Nano‐Biostimulant to Enhance Their Growth/Tolerance under Different Abiotic Stresses

Nanomaterials such as MXene and MBene have been shown to effectively alleviate abiotic stresses, including drought, salinity, and heavy metal toxicity, through enhancing photosynthetic efficiency, increasing water‐use efficiency, and mitigating oxidative stress by activating antioxidant mechanisms. Additionally, they can maintain ion homeostasis and stabilize cellular membranes, enabling plants to sustain physiological functions under extreme environmental conditions and safeguarding crop yields in a challenging climate change. Hence, their stability and bioactivity are crucial for long‐term applications in complex bio‐systems. With this in mind, we have significantly improved these properties in our recent work.^[^ ^66^ ^]^ In particular, building on our previous findings on novel MXene applications for plant nano‐immunoengineering, antimicrobial defense enhancement, and biostimulation, we designed and developed a newer generation of these layered materials, “chiral‐engineered MXenes”.

**Figure 22:**
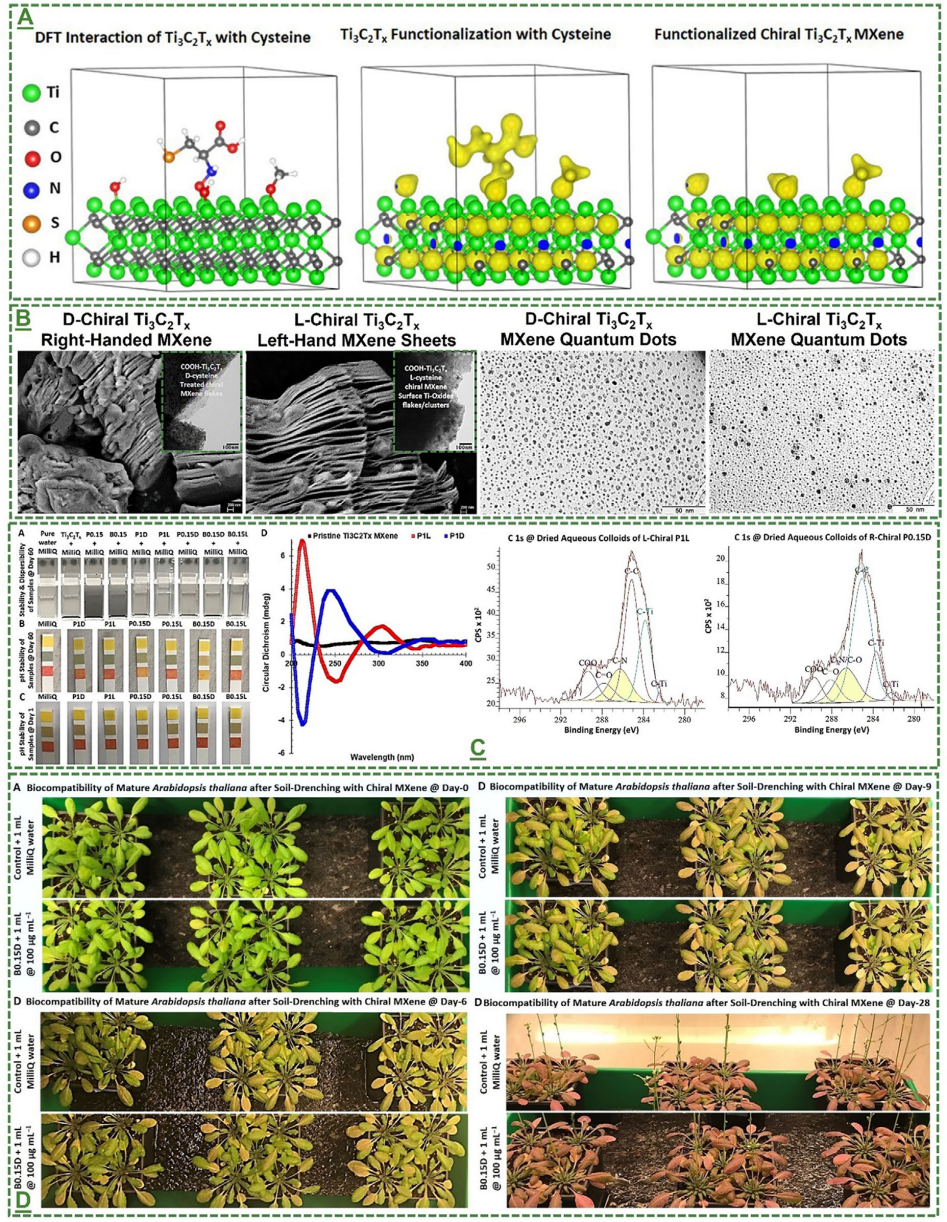
A) Representation of the reported DFT calculations for surface binding of an amino acid to Ti_3_C_2_T_x_. B) The represented TEM images of as‐prepared left or right‐handed chiral MXenes. C) represented colloidal dispersibility, pH measurements, and left‐ or right‐handed asymmetric chiral‐functionalized Ti_3_C_2_T_x_ MXene heterostructures compared to pristine symmetric and their long‐term colloidal properties. The CD spectra analyses of these chiral‐engineered MXenes were also represented to highlight their stability for months at 4 degrees. D) The assessments of short‐, mid‐, and long‐term phyto‐compatibility of these chiral MXenes aqueous colloidal suspensions with *Arabidopsis thaliana* seeds (Col‐0), supporting their seed coating, seed sprouting/germination, and seedling maturation. A–D) Reproduced with permission.^[^ ^66^ ^]^ Copyright 2025, Wiley‐VCH GmbH.

In particular, we introduced new advances in the engineering design and multi‐biostimulant applications of right‐ or left‐handed chiral Ti_3_C_2_T_x_ MXene nanosheet‐derived quantum dots heterostructures. Using a facile, versatile, and universal strategy, it is showcased that inducing this rational chirality, as a pivotal natural specification of living systems and most bio‐macromolecules, into the structure of Ti_3_C_2_T_x_ nanosheets (as the most common MXene prototype) can contribute to constructing stable chiral‐active mixed‐dimensional biomaterials with enhanced colloidal dispersibility, durability, and bioactivity in plants. It was elucidated that treating the seedlings and plants with these chiral‐modified MXenes could enhance their resilience/resistance against various abiotic stressors, protecting them against severe drought, salinity, or light stress conditions.^[^ ^66^ ^]^ Through, amino acid ligands chirality‐transformation strategy, we created several asymmetric MXene‐based biomaterials with the optically/structurally left‐ or right‐handed properties beyond MXene nanosheets’ typical specifications. As can be seen in our circular dichroism (CD) and TEM microscopy data of the selective chiral MXenes, compared to pristine Ti_3_C_2_T_x_, confirms an efficient chirality‐induction into these nanosheets, with stable colloidal dispersibility and pH‐compatibility properties (almost similar pH to ultrapure water) for months (see **Figure** **22**). As shown, our biocompatibility assessments of these chiral‐engineered materials suggest excellent short to long‐term properties at the tested concentration (100 µg mL^−1^).

Throughout density functional theory (DFT) calculations and experimentations, the synthesis, surface‐dependent electrochemical impedance properties, and multiple phyto‐activity impacts of optimal chiral MXenes were reported within different climate chambers and greenhouse plant‐based bioassays.

Beyond offering a high level of biocompatibility with the seeds/seedlings/plants, we proposed and defined mechanisms behind enhancing their tolerance to abiotic stresses alongside improving their growth under a multifunctional biostimulant activity. We showed that the ROS production and eliciting‐inducing responses could readily and effectively promote the resistance of *Arabidopsis thaliana* plants, including their viability, growth, and development, such as natural aging, maturation, and flowering stages. Indeed, the rationale and significance behind introducing the stable chirality into the structure of MXene revolved around this traction that we wanted to develop a highly biocompatible carbon‐based material as next‐generation plant nano‐stimulators and abiotic‐resistance boosters towards future applications in agriculture, either alone or in multimodal treatments, when their safety has been proven sufficiently for agricultural inputs.

**Figure 23:**
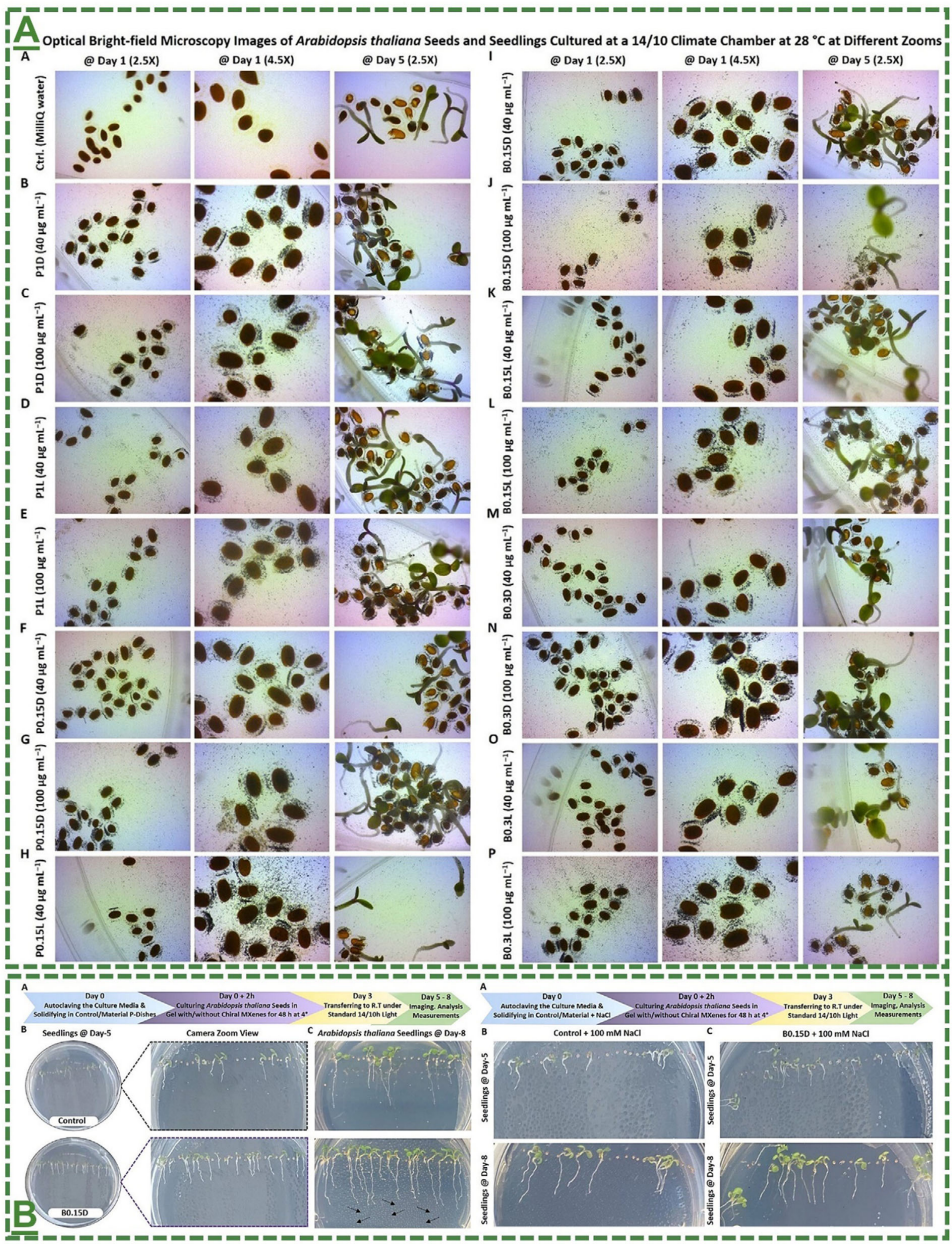
A) The represented microscope/camera images qualitatively depict the enhanced seed‐to‐seedling transition impact of the material at different developmental stages. B) The experimental timeline and biostimulant impact of chiral‐modified MXenes on significantly enhancing the growth and maturation of *Arabidopsis thaliana* seedlings under both normal growth and salt stress at different time points at a dose of 88 µg mL^−1^ for MXene and 200 mM NaCl in salt‐stress assays. A,B) Reproduced with permission.^[^ ^66^ ^]^ Copyright 2025, Wiley‐VCH GmbH.

**Figure 24:**
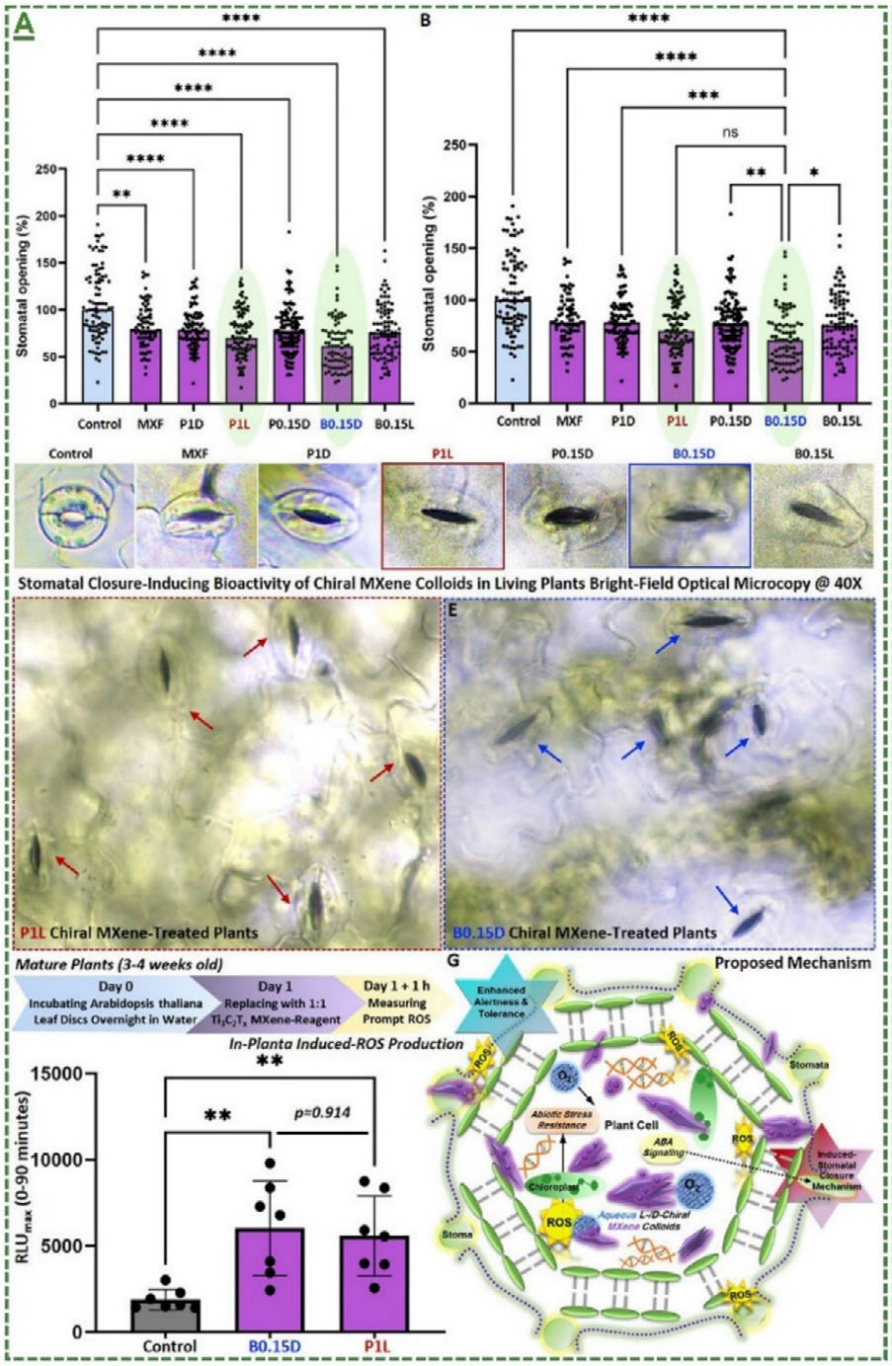
A) Stomatal‐closure‐inducing mechanism of these right‐/left‐handed chiral MXenes compared to the control groups. Microscopic images show induced stomatal closure reposed in foliar‐sprayed plants. Experimental timeline and eliciting bioactivity of these chiral‐engineered MXenes in *Arabidopsis thaliana*. It depicts the proposed defense/growth‐enhanced mechanisms. (A): Adapted with permission.^[^ ^66^ ^]^ Copyright 2025, Wiley‐VCH GmbH.

**Figure 25:**
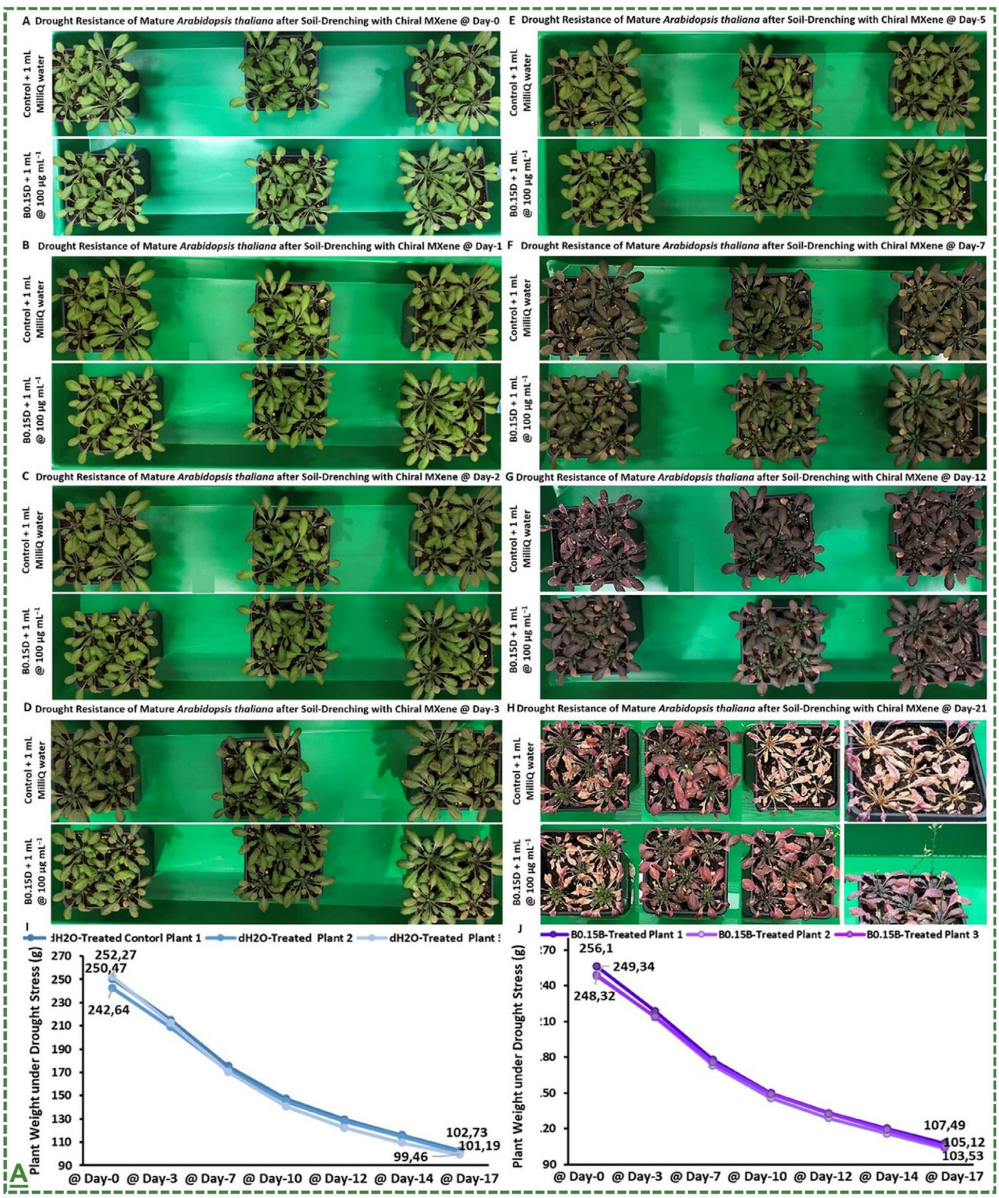
A) In planta short/mid/long‐term drought stress‐resistance bioactivity of chiral MXene Arabidopsis *thaliana* plants compared with control groups in the greenhouse at different time points up to 21 days post‐applied soil‐drenching. These data highlight the positive impact of chiral MXenes on enhancing plant tolerance to severe drought conditions.  Treating the plants with one‐pot foliar spraying with this chiral MXene (≈1 mL) could enhance their growth and flowering stages compared to water‐sprayed plants at the tested dose (100 µg mL^−1^). A) Adapted with permission.^[^ ^66^ ^]^ Copyright 2025, Wiley‐VCH GmbH.

**Figure 26:**
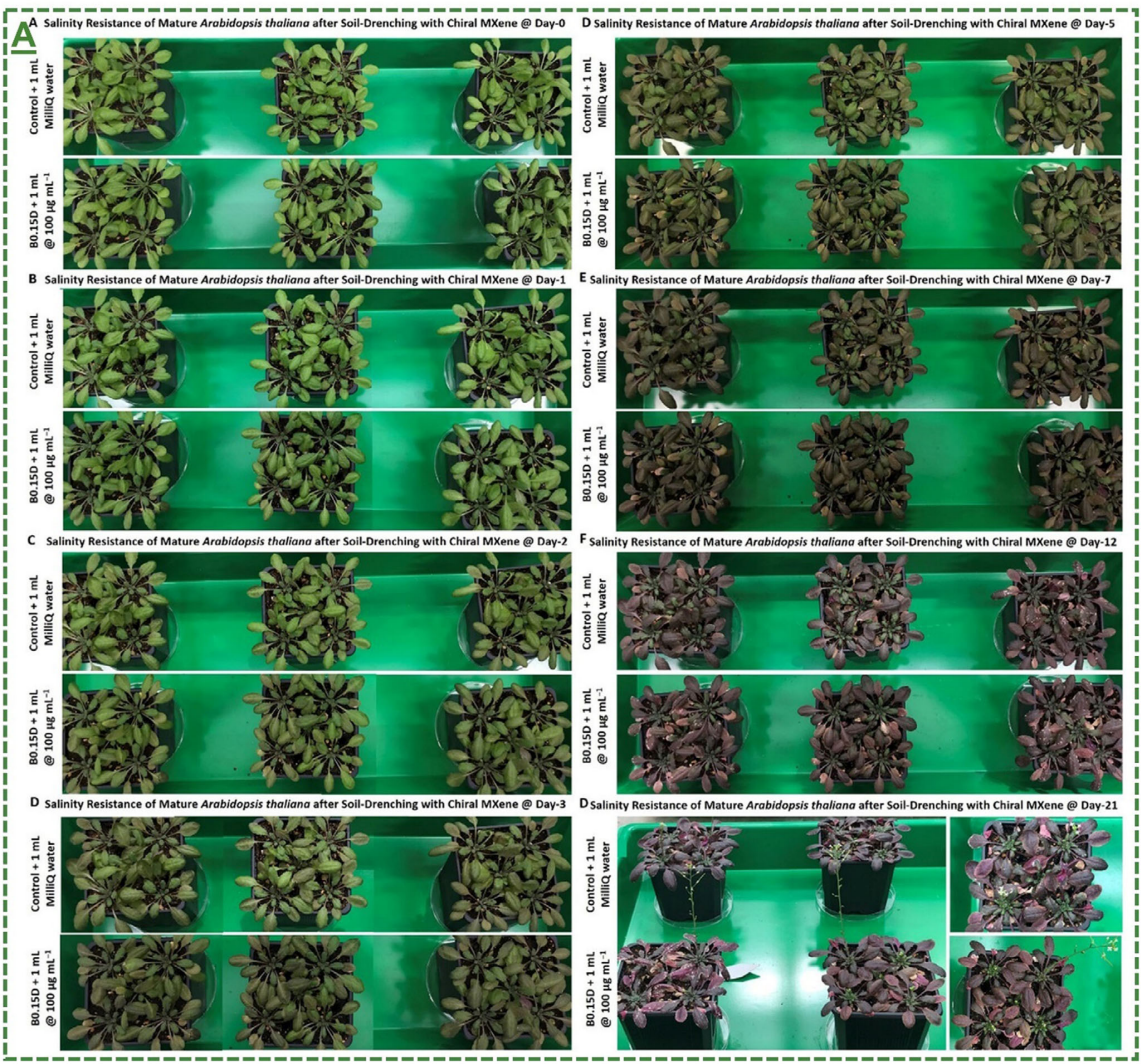
A) In planta short/mid/long‐term light salt‐resistance bioactivity and flowering measurement analyses of chiral MXene treated‐*Arabidopsis thaliana* plants compared with control groups. Continuous exposure to light stress in chiral MXene‐treated plants at different time points up to 21 days was elucidated with enhanced resistance in plants post‐soil‐drenching. These data highlight the positive impact of chiral MXenes on boosting the plant tolerance to excessive light stress without significant visible adverse effects on overall plant growth. Rather, treating the plants with one‐pot foliar‐spraying with this chiral MXene (≈1 mL) could enhance their growth and flowering stages compared to water‐sprayed plants at both tested concentrations (100 µg mL^−1^). A) Adapted with permission.^[^ ^66^ ^]^ Copyright 2025, Wiley‐VCH GmbH.

**Figure 27:**
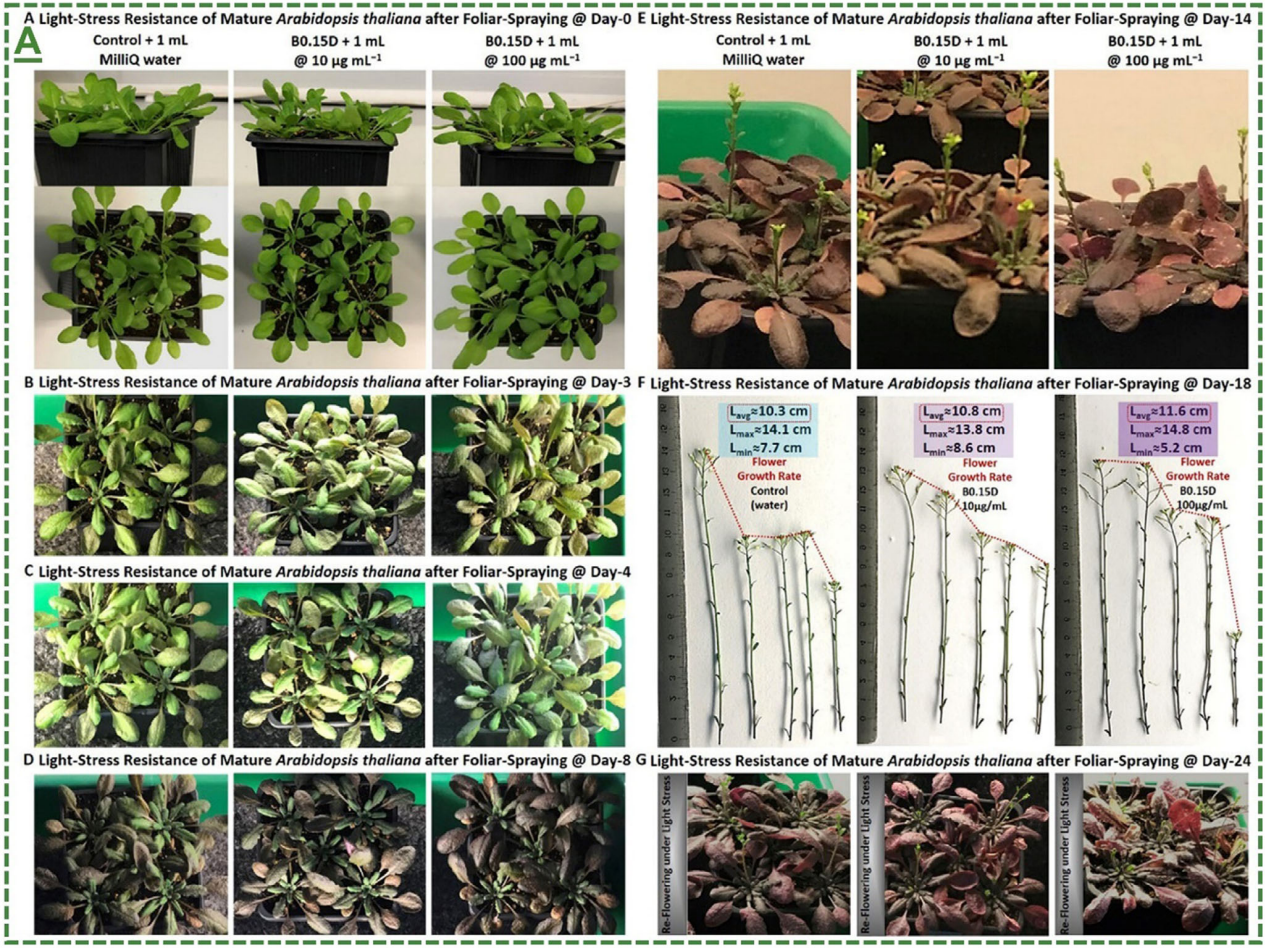
A) In planta short/mid/long‐term light stress‐resistance bioactivity and flowering measurement analyses of chiral MXene treated‐*Arabidopsis thaliana* plants compared with control groups under greenhouse in chiral MXene‐treated plants at different time points up to 17 days post‐applied soil‐drenching. These represented data highlight the positive impact of chiral MXenes on enhancing the plant tolerance to excessive light‐stress incubations without significant visible adverse effects on overall plant growth. Rather, treating the plants with one‐pot foliar‐spraying with chiral MXene (≈1 mL) could enhance their growth and flowering stages compared to water‐sprayed plants at both tested doses (10 and 100 µg mL^−1^). A) Reproduced with permission.^[^ ^66^ ^]^ Copyright 2025, Wiley‐VCH GmbH.

Given that the chirality is a crucial factor in the design and engineering development of many agrochemicals, plant biostimulants, and amino acid‐containing agro‐fertilizers, and based on our data, it is anticipated that chiral MXenes (and potentially chiral MBenes) may support the required bioactivity functions for enhanced interactions with plant cells/enzymes, boosting their defense mechanism and stress tolerance, as well as nutrient uptake processes for growth enhancement. This pilot study showed that the tested materials did not cause any significant cytotoxicity or growth‐inhibition effects compared to control groups at tested concentrations (up to 88 µg mL^−1^ in co‐culture settings and ≤100 µg mL^−1^ in foliar application to plants (around 1mL, single treatments). As represented in **Figures** **23**, **24**, **25**, **26**, **27**, our data also suggested a remarkable biostimulant activity of optimal right‐handed chiral MXenes to significantly increase the germination/sprouting of the model col‐0 seeds and their maturation and development under both normal growth and salt stresses in the presence of sodium chloride solutions at doses up to 200 mM. In particular, our data showed a significant increase in the median root size of these seeds from around 98 to 131 mm in regular culture and from ≈75 to 86 mm under the applied salt stress in chiral MXene‐treated groups compared to controls. Additionally, the seed germination of these seeds is observed with a remarkable increase from ≈40% to over 70% (approximately twofold improvement) in chiral Ti_3_C_2_T_x_‐treated seeds under salt effect. Moreover, our results suggested the impact of chiral‐modified MXene on relatively enhanced drought tolerance for weeks and survival under continuous light stress of zero‐darkness incubations (only day‐cycles), tolerance/resilience of mature Col‐0 model plants (3‐4‐week age).^[^ ^66^ ^]^ The obtained multisystem of chiral MXene nanosheets and quantum dots, and in situ self‐assembly with stable surface metal oxides takes advantage over conventional carbon‐based materials for bio‐applications, where the higher stability of aqueous nanomaterial suspensions against excessive oxidation, degradation, and biological compatibility are key factors.

In summary, to the best of our knowledge, this work presented the first application of chiral‐engineered MXenes for protection and stress tolerance enhancement of plants against severe abiotic conditions. The plants treated with these MXenes have behaved strongly against simulated harsh environmental conditions, including salinity, drought, and light stress. This can pave the way toward further investigations and product development to enhance plant defense, increase their yield and production capacity, while reducing the consumption of agrochemicals.

### 3.11 Reported Application(s) of MBene for Improved Plant Tolerance and Growth

**Figure 28:**
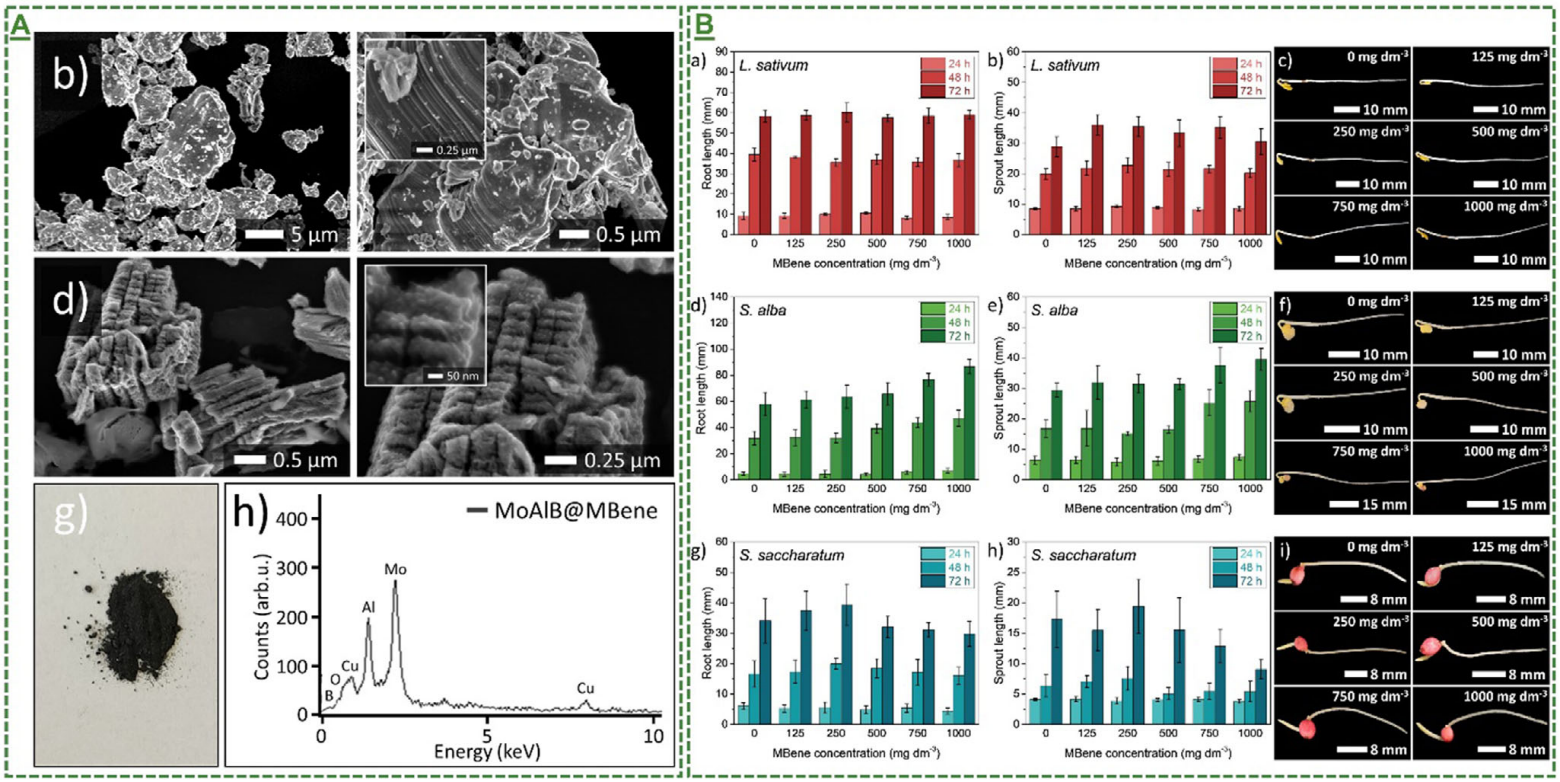
A) Morphology and microstructure characterization of this specific MAB phase before and after conversion to MoAlB MBene nanosheets in powder form with resulting EDS elemental composition spectra. B) The impact of the MoAlB‐derived MBene at various doses on the growth of root and sprout of *L. sativum*, *S. alba*, and S. saccharatum. These represented images displaying plants after 48 h incubation with the materials at different doses ranging from 0 to 1000 mg dm^−3^. For presentation consistency with the other figures, the black dashed lines of these figures in the original publications have been removed with permission from the work's Corresponding Author. A,B) Reproduced with permission.^[^ ^73^ ^]^ Copyright 2025, Elsevier B.V.

More recently, in an innovative study by Jakubczak and Jaztrzebska et al., a growth‐promoting impact of an MBene formulation was reported in higher plants.^[^ ^73^ ^]^ They have shown microscopic observations of detailed MBene‐stimulated lateral root growth (see **Figure** **28** for the adapted data). Throughout mass analyses, they demonstrated the impact of these MBene nanosheets on vitality preservation and the carbon cycle. As shown in Figure S22 (Supporting Information), they also studied chlorophyll and fluorescence studies, revealing that these MBene sheets exhibited positive/neutral impacts on tested plants. In particular, they reported observation of significant variations in chlorophyll extracts obtained from the plant's shoot parts, which were grown in soil enriched with MoAlB@MBene nanosheets at relatively low doses of 125 and 250 mg dm^−3^, suggesting the uptake/penetration of the flakes into the soil system. They reported that these MoAlB@MBene did not impose any significant adverse impact on chlorophyll contents within the studied plants. They provided the morphology and microstructure characterization of this specific MBene nanosheets in powder form with resulting EDS elemental composition spectra. They also evaluated the effect of MoAlB‐derived MBene at various doses on the growth of the root and sprout of *L. sativum*, *S. alba*, and *S. saccharatum*. These represented images displaying plants after 48 hours of incubation with the materials at different doses ranging from 0‐1000 mg dm^−3^. Their results also showed that soil treatment with MoAlB@MBene at tested concentrations didn't remarkably alter the fluorescence ability of *S. alba* and *S. saccharatum* plants compared to the reference groups. They conclude the composition and size‐dependent properties of these materials in tested plant‐soil models, paving the way for further mechanistic studies on MXene/MBene‐based biomaterials.^[^ ^73^ ^]^

In summary, to the best of our knowledge, this work presents the first application of MBene nanosheets for plant biostimulation. The plants treated with this MBene have grown better without causing any significant adverse effect on these plants at tested doses and treatment durations. Their results presented a fundamental understanding of how MoAlB@MBene impacts the plants’ root/shoot and their surrounding soil environment. This study has reasonably concluded that by aligning material properties with environmental biocompatibility and safety requirements, quality 2D nanomaterials can be developed to improve sustainability in agriculture. It opens up a new avenue for further mechanistic studies and optimization. In particular, the long‐term interaction of MBenes with different plant types and their bioactivity impacts on actual agricultural settings is at the forefront of the field and is highly recommended to be further investigated in future work.

### 3.12 Key Discussions and Summary on Bio‐Property/Application of MXene/MBene in Agriculture

After reviewing the reported results, we discussed how these materials can be compared functionally. Table 2 summarizes the main properties of MXenes and MBenes in agricultural protection and plant biostimulation. For agricultural use, it is important to select the appropriate chemical composition and surface/structure modification of MXenes or MBenes based on the particular criteria of each application. This choice and confirmation of their superior properties require more experiments, even though we have provided some scientific speculation here.

The main comparison between MXenes and MBenes in agricultural settings and plant biostimulation centers on their chemical composition and synthesis quality, which directly influence their bioactivity properties. In some applications, bioactive formulations of MXene are expected to slightly outperform due to their established production/modification routes, larger chemical diversity, excellent processability, and nano‐carbon/nitrite crystalline properties. MXenes have been shown to interact smartly with plants, exhibiting multiple antimicrobial modes of action and regulatory bioactivity, thereby enhancing their immunity and defense/growth mechanisms. MXenes also have a relatively higher tendency to react with oxygen molecules in air and aqueous media. This point has been reported to be significantly improved by surface modification and structural functionalization. However, gradual oxidation and metal oxide formation can be advantageous for certain applications that are not reliant on the material's durability or long‐term stability. Indeed, bio‐degradation of MXenes may benefit specific agro‐environmental applications by boosting the bio‐clearance process of surface‐active nanomaterials.

On the other hand, specific MBenes can potentially outperform or be highly competitive with MXenes, due to their higher mechanical properties (M─B versus M─C). Especially for such applications, longer‐term stability and durability of bioactive nanomaterials are essential. Controlled delivery and sustained release of agrochemicals is a right example of this physicochemical preference in nano‐designs. That's why the field has remained dynamic in both sectors with the aim of further enhancing their properties to the maximum possible levels, and the emergence of novel applications. As of now, the field's literature suggests that bioactive MXenes and MBenes compositions exhibit competitive phyto‐environmental compatibility and applications. However, their bio‐profiles have been shown to have slight differences. These differences are due to their chemical specificity in oxidation and hydrolysis in aqueous media. In particular, it is understood that the possible release of metal and metal oxide ions from MXenes/MBenes occurs over time in a relative manner. Rational surface modification and post‐functionalization strategies could significantly improve these properties.

From a chemistry viewpoint, the non‐toxic boride‐based compositions often impart more inertness and resistance to oxidation or hydrolysis in air and water. This inherent tendency can minimize the release of transition metal/metal oxide ions from their surface, decreasing sustained oxidative reactivity and excessive oxidative stress in plant cells/tissues. Likewise, the MBenes’ surface can also be functionalized for improved biocompatibility and bioactivity. Hence, their properties have made them suitable for certain plant biostimulation applications (Figure S23, Supporting Information). This uniqueness extends to boron atoms, which are an essential micronutrient for plant growth. It has a pivotal role in plant membranes, cell wall structure, and metabolic regulation. In theory, the boron atoms in the structure of MBenes may serve as a sustained and slow‐release source after treating the plants or direct soil irrigation. This property is beyond the inherent ability of layered materials to act as a large‐area active agent to absorb/deliver soil nutrients to the plant's root/shoot parts. Indeed, the presence of boron may potentially boost plant biostimulation activities. Additionally, degradation of MBenes is expected to be biocompatible with the soil environment. In particular, by overtime degradation, MBenes in plant/soil media, they decompose boron and metal ions, which can be absorbed by plants as a beneficial supplement. While by oxidative decomposition of MXenes, they are typically degraded into carbonates or amorphous carbon‐based compounds, and transition‐metal/metal oxides don't offer micronutrient benefits. This rationale needs clarification by detailed mechanistic experiments.

Moreover, due to the inherently higher antimicrobial activity of boron (compared to carbon and nitrogen), MBenes may offer enhanced antimicrobial modes of action, particularly against phytopathogens, which is highly beneficial in agriculture. Lastly, specific compositions of MBenes have higher electronic properties, such as higher conductivity and distinct band structures, which may support plant biostimulation and signaling. Different surface chemistry of boride compared to carbide/nitride may also impact modulating plant defense mechanisms and associated pathways under stress conditions. In addition, bioactive MBenes may also offer antimicrobial activities or synergistic effects due to their unique electronic properties, which may contribute to enhancing their interactions with pathogens. The high mechanical properties of MBenes may also support their sensing applications, where higher durability and temperature resistance of the materials in the environmental conditions are key for agricultural applications. In summary, both MXenes and MBenes have unique specifications, which make them potential candidates for nano‐agricultural applications. MXenes have shown higher scale‐up production capacity, and their cost consideration might outperform MBenes.

### 3.13 Proposed Mechanisms and Synergistic Effects of MXenes/MBenes with Agrochemicals

Fundamentally, the surfaces of MXene/MBene materials are enriched with various bioactive functional groups, including −OH, −F, −O, etc. These surface terminations, along with their high bioactive surface area, enable spontaneous binding with different molecules or compounds. This inherent property is highly beneficial in drug attachments and the controlled delivery or sustained release of distinct agrochemicals. Their surface properties and chemical composition dependently support hydrogen binding, π–π stacking, van der Waals, or electrostatic interactions with different agrochemical types, such as plant antimicrobials, pesticides, and soil fertilizers. Beyond physical absorption, their surface tends to chemically adsorb and interact with pesticide reactive functional groups, possibly through ionic bond formation. Besides, their unique morphology provides a large loading area through encapsulation or intercalation between the layers. This nano‐micro or nano‐bulk compound integration can efficiently enhance their carrier and sustained slow‐release profiles for reducing the required doses and avoiding multiple treatments.

Moreover, loading agrochemicals between MXenes/MBenes layers acts as a physical barrier. This effectively slows their release into the soil and surrounding environments. The controlled release capability may enhance the efficiency of agrochemicals. It enables sustained or stimulus‐responsive release, reducing the need for frequent treatments. This nanocarrier strategy can ultimately reduce dosages, minimizing adverse health and environmental impacts of large‐scale agrochemical consumption. More importantly, innate antimicrobial and biostimulatory properties of MXenes/MBenes can synergistically promote plant growth and protection. Such multi‐benefits support applications both alone and in combination therapies. In particular, applying a bioactive MXene/MBene in plants without pesticides or fertilizers relies on direct antimicrobial activities and ROS generation. When embedded with agrochemicals, their mechanisms act synergistically for both physical disruption and chemical toxicity. Another possible scenario turns on the fact that encapsulation of sensitive agrochemicals within MXene/MBene layers can also effectively protect them from UV degradation, volatilization, hydrolysis, or harsh environmental conditions, increasing their lifespan and reducing the frequency of treatment applications.

### 3.14 Proposed Sex‐Specific Dependent Effects of MXenes/MBenes on Plants

In this section, we have strived to propose this innovative statement on whether and how MXenes or MBenes potentially affect the male, female, or male/female plants. The comparison may help further understand how the chemical composition, size, shape, and morphology of bioactive materials influence their plant‐immunoengineering or biostimulation properties. In this regard, an important question is the impact of each MXene or MBene on modulating defense or growth‐related gene expression and phytohormone regulation in plants in a sex‐dependent manner. This rational and novel topic has the full capacity to pave the way for opening up new avenues of study to uncover these sex‐specific effects in plant treatment by MXene/MBene nanomaterials. It can influence their future applications in crop production, where sex‐specific characteristics of agricultural plants perform differently by MXene/MBene‐based treatments.

This aspect is highlighted by the phenomenon that many plants are dioecious, having distinct female and male individuals. They naturally respond to environmental factors and interaction with chemicals/compounds in a sex‐dependent fashion. To date, there is no particular study in the literature that specifically focuses on the sex‐dependent effects of any MXene or MBnene on plants. Indeed, most of the performed research has been conducted on common model plant/crop species, which are often monoecious or hermaphroditic. Therefore, in this review, we comment on the idea of exploring broader plant physiology and nano‐interactional impacts of MXenes/MBenes for sex‐dependent effects.

This hypothesis‐based concept can be influential in several ways, including differential uptake and translocation, gene expression, hormonal regulation, and basic oxidative stress or antioxidant responses. In particular, the root architecture and expression/structure of transport proteins may differ in male and female plants. If plants root‐absorb the MXene/MBnene nanosheets or particles, these differences may impact the accumulation rates. Besides, as plant sex is regulated by specific phytohormones, whether treating the plants with MXenes or MBenes could eventually affect their hormone signaling or biosynthesis (as our study and other reports suggested some hormone regulation), this effect could differ in male or female plants, which needs to be understood and considered for real‐world applications. Moreover, it is reported that male and female plants have different antioxidant enzyme‐based activities. If MXenes/MBenes are able to generate ROS and induce eliciting or priming activities in plants, the antioxidant capacity and resilience/response to RSO may differ in female and male plants, affecting their alertness and defense against different stresses. By understanding these questions and unleashing the associated mechanisms and sex‐specific responses, precision agriculture strategies can be developed to treat specific plants with a certain composition or form of MXenes/MBenes.

### 3.15 Cost Consideration and Proposed Outperformance of MXenes/MBenes Over Conventional Nanomaterials

Notably, cost consideration, economic aspects of large‐scale production of MXenes/MBenes, and comparison of the superiority of their bio‐properties over other nanomaterials are not the focus of this work. However, due to the significance of these factors/parameters and their determinative roles in advancing the nano‐biotechnology of MXene/MBene field in agriculture, we attempted to briefly discuss some of these points in this sub‐section. In particular, multiple criteria are important and need to be considered for practical applications. The first note relies on the newer generation of these low‐dimensional materials, as compared to previous generations. The emerging field of MXenes and MBenes has rapidly progressed and is expected to accomplish further synthesis optimizations and price reductions in the near future, probably through more advanced methods and the use of recycled precursors. This includes both optimizing the production routes by using more facile, faster, and straightforward protocols, as well as using processor compounds obtained from the industrial/biological wastes or recyclable materials.

In this regard, several recent studies focused on evaluating the production costs of MXene and reducing them through these considerations.^[^ ^109^, ^110^ ^]^ In particular, each step cost has been explored and evaluated by a detailed evaluation of their synthesis processes. They elaborated on the expenses of each stage/parameter involved in the production of MAX phases, as well as the costs required for converting them into MXene‐based nanosheets. Additionally, these publications have highlighted the costliest steps towards further optimization and price reduction. It is specifically noted that the majority of these costs are allocated to precursors (34%), electricity in the thermal process (30%), and etching treatment (26%). It has also been proposed that the actual costs of 7% for step‐characterizations and 3% for human work expenses. Other steps, including ball milling, mixing, rinsing, drying, grinding, and filtration, are reported to be not considered as notable costs (see the original articles for detailed notes). According to their analysis, the total cost of producing around one gram of Ti_3_C_2_T_x_ MXene nanosheets is determined to be approximately 20 US$. As recommended, the final price could be reduced by further research and development, which not only satisfies economic and commercial aspects but also enhances the long‐term biocompatibility of these materials.

The second point that further highlights the potential of MXenes/MBenes relies on their unique properties, including innate hydrophilicity, surface termination variation, higher loading capacity, flexibility for functionalization, and availability of diverse compositions. These specifications have been shown to be even further tunable, enabling them to be dispersed in water without the necessity of adding surfactant, which can not only contribute to reducing their final products’ cost, but is also highly beneficial in reducing their chemical complexity, increasing biological properties, and improving climate change and the environment.

### 3.16 Proposed Plant Nano‐Uptake Mechanisms of MXenes/MBenes

Moreover, selective nanomaterials can be taken up by plants through distinct mechanisms. Plants can absorb them through the root/shoot parts. This likely happens through surface membrane interactions and other plant cellular channels, or through stomatal pathways. Therefore, it is crucial to control the interaction of nanomaterials with plants and their accumulation in the environment. This should be done in terms of both concentration and biocompatibility properties. Nanomaterials can also frequently be found in water or soil, where they may interact with plants and agricultural crops. Notably, plant defense systems and natural mechanisms often efficiently absorb essential nutrients and soil molecules. Their interaction with beneficial microorganisms and even invading phytopathogens can also be relatively successful under certain conditions, especially at early stages. Nanoscale particles or engineered nanomaterials can interact strongly with phytocells and may eventually move to different plant parts. This can induce beneficial responses or impose adverse impacts on plant and surrounding systems (Figures S24–S27, Supporting Information), where the adapted data tables represent some of these influences of other nanomaterials.

Compared to bulky compounds, the nanostructured materials have enhanced physicochemical properties. This promotes their reactivity to the plant's root or shoot. Plants commonly recognize nanomaterials based on three main characteristics: small size, shape, and unique surface chemistry properties such as charge, hydrophobicity/hydrophilicity, mobility, and dissolution within diverse biological systems. Direct diffusion of nanomaterials through the phospholipid bilayer is likely among the most common mechanisms. Penetration via endocytosis and plasma‐membrane inward movement around the nanomaterials is another possible mechanism. Another scenario involves ion channels and aquaporin, though this is less reported for interactions with plant systems. For newer generations of nanomaterials, such as MXenes and MBenes, there are fewer publications about their effects on plant systems compared to conventional nanomaterials. More studies are still needed to better understand these under realistic settings for a fair comparison of their biological and immunological profiles with other nanomaterials. These superiority validations, along with the cost considerations and environmental safety, are the key requirements for their future translation into agricultural settings.

### 3.17 Proposed Design of Experiments and Mathematical/Machine Learning/AI‐Based Methods in Predicting/Optimizing MXenes/MBenes’ Properties

Optimizing the synthesis parameters of MXene/MBene synthesis, including etching time, etchant concentration, etching stirring rate, temperature, and hydrothermal settings, as well as post‐functionalization processes, is essential. It can be performed through mathematical models or design of experiments (DOE). Beyond that, the application of machine learning and artificial intelligence (AI)‐based methods can be considered to predict, optimize, or maximize the outputs. In particular, applying standard DOE and Plackett‐Burman Design or Taguchi optimization can contribute to reduce the trials based on the suggested algorithm to minimum possible experiments when three or more parameters are effective (e.g., L3*3: Taguchi's L9 orthogonal array (9 selections over 27 possible experiments), L4*4: L16 trials over 64 probability, CMF1‐8: five individual parameters of 2 variable for each,). This pre‐filtering of a large experimental design can provide directions towards setting initial experiments and reasonably reducing the variable possibilities (e.g., 9 versus 27 or 16 versus 64). The obtained outputs are formulated to calculate the signal‐to‐noise ratio and maximum outputs at that level using validation.

Furthermore, machine learning and AI‐based methods, such as adaptive neuro‐fuzzy inference systems, artificial neural networks (ANN), multi‐objective particle swarm optimization (MOPSO), support vector machines (SVMs), and gene expression programming (GEP), can be applied to further optimize the synthesis parameters or maximize the material's outputs, including biocompatibility and bioactivity in plants. Moreover, integrating these approaches with materials calculation and computation methods, such as density functional theory (DFT), can provide a robust combination for optimization/prediction of MXenes/MBenes. By accurate testing and training modeling systems within the performed model, different properties of these materials can be formulated and optimized. This is highly important due to the nature of plant experiments, which can be time‐consuming and, in some cases, costly. Hence, reducing the experimental procedures and improving the functional properties are assets for agriculture. By testing a training model, the prediction of each of the aforementioned properties of MXenes/MBenes may be possible. For instance, eliciting, priming, germination rate, and gene regulation can be formulated for each MXene or MBene composition, predicting the performance of each particular output, for example, based on the working dose or treatment time.

## 4 Challenges and Outlooks in MXene/MBene‐Based Interventions into Agricultural Settings

MXenes and MBenes, two rapidly expanding families of low‐dimensional functional materials, have already shown profound potential for applications in a wide range of fields, more recently in nano‐agriculture. Despite the advances obtained for addressing long‐standing challenges in today's agriculture, their practical applications for real‐world uses have still faced significant challenges. This includes the necessity of further validations on their long‐term biocompatibility and health‐related safety. The large‐scale production in a robust manner, considering batch‐to‐batch variation and production costs, is another current challenge of this nano‐biotechnology. These considerations are essential for optimizing the quality and cost of end products, further transitioning them from laboratory research‐scale experiments to quantity and substantiated product development. Over recent years, there has been a universal effort to reduce these limitations and produce MXenes (and MBenes) in larger quantities and with improved quality using greener synthesis approaches. Enhancing their biocompatibility and bioactivity to higher levels has been another focus of the field.

From a plant biology and agricultural perspective, parts of these efforts have been carried out to reduce concerns regarding the potential nanotoxicological effects of MXenes/MBenes on plants or soil media at non‐controlled doses. Despite the majority of publications in the literature, a limited frequency of evidence has been reported on the adverse impact of these nanomaterials. In particular, it was reported that even though pristine Ti_3_C_2_T_x_ MXene nanosheets (non‐modified MXenes) possess excellent biocompatibility with diverse biological systems, including plants and soil media at specific dose thresholds, their long‐term exposure at higher doses can cause slight toxicity effects on specific plants. In this regard, Feng et al. reported that despite the high biocompatibility of Ti_3_C_2_T_x_, especially at concentrations lower than 50 mg kg^−1^, water hyacinth likely recognizes these nanosheets as a foreign substance or pollutant.^[^ ^111^ ^]^ Their results suggested that these flakes could be root‐absorbed by this plant type, but did not appear to uptake/translocated to its upper shoot parts. As a result of this nano‐interaction with this particular plant's root, it is reported that long‐term exposure (above 10 days) at higher concentrations of the nanosheets caused slight structural damage to the plant's root hairs in a size‐dependent manner, resulting in a coarser surface topography. The study was then concluded based on these preliminary data that while Ti_3_C_2_T_x_ MXene nanosheets demonstrate a degree of biocompatibility to this plant, it is likely possible to exert a mild adverse effect on its overall growth and physiological conditions.

Meanwhile, a recent study by Zhou et al. reported the effects of different soil colloids on the aggregation or degradation of non‐modified Ti_3_C_2_T_x_ MXene sheets, which is a critical issue for their environmental applications.^[^ ^112^ ^]^ In particular, the authors studied how these nano‐interactions with soil compounds/molecules affect the stability and aggregation/degradation of MXene nanosheets, simulating their actual interactions. They elucidated the role of different soil colloids, such as clay, silt, or organic materials, on the aggregation of aqueous MXene particle dispersions within the soil matrix. This innovative study evaluated the colloidal dispersion and degradation kinetics of Ti_3_C_2_T_x_ in groundwater by considering the impact of soil colloids derived from sodium humate, montmorillonite, and natural soil as a more reliable control under variable experimental conditions. Their results showed that the affinity of these soil colloids treated with Ti_3_C_2_T_x_ nanosheets was higher in sodium humate, montmorillonite, and natural soil. The increasing dose of sodium humate has been reported to contribute to further disaggregation of Ti_3_C_2_T_x_ MXene, likely through enhancing the electrical or steric repulsive forces in these nanosheets. They also stated that montmorillonite and natural soil contributed to the hetero‐aggregation of Ti_3_C_2_T_x_ by elevating their collision frequency at different levels. They have also suggested that these effects could be improved through surface coating or interaction with the surface functional groups available in MXene nanosheets.

In a nutshell, they speculated that colloidal soil molecules/compounds can further promote the degradation of carbon‐based nanomaterials, including Ti_3_C_2_T_x_ MXene, in a pH‐dependent fashion. They reported that the affinity of this material with natural soil has been found to have a lower impact, but with some concerns. These technical points cannot be ignored and must be improved in the case of these specific plants and other similar types. This limitation of material specifications sheds light on the importance and key role of surface modification and post‐functionalization of MXenes or other bioactive nanomaterials, such as MBenes, to improve their biocompatibility properties. It is noteworthy that the majority of reported works in the literature suggested high biocompatibility behavior of MXenes/MBenes with tested plant models, especially at controlled doses (mostly ≤100 µg mL^−1^ for Ti_3_C_2_T_x_ and ≤250 mg dm^−3^ for MoAlB@MBene). Therefore, it is reasonable to hold the promise for bioactive MXenes/MBenes with a focus on further improving their long‐term biocompatibility (e.g., for up to several months of treatment or probably within a few years). This opens extensive research possibilities for further development and property improvement aspects. Besides, new investigations are recommended to assess the potential impact of MXenes/MBenes for enhancing plant defense/tolerance/growth‐related mechanisms under multiple stress conditions. This can be a combination of the same or different types of stress. We also recommend further study of their bioactive properties in actual crops, such as tomato or potato, to evaluate their safety and functional efficiency in real‐world scenarios.

Beyond that, there have been ongoing research investigations on novel applications of MXenes and MBenes, particularly in the related sectors of agriculture and the environment. In addition to improving their long‐term biocompatibility and safety efficacy through both experimental and computer‐assisted modeling and bioinformatics have paralleled nanotechnology to effectively develop the best possible strategies, control/improve interactions, and maximize the bioactive properties of novel materials for specific bio‐related applications. To our best current knowledge, no report has reported the AI‐based applications for optimizing MXenes/MBenes in the direction of plant studies. Therefore, considering the progress of AI‐based applications in different aspects of today's technology, applying these studies is promising to further advance the field and reduce its current limitations. Moreover, focusing on improving the environmental aspects of MXenes/MBenes synthesis and waste disposal is another asset that could progress the current limitations by developing/applying more eco‐friendly synthesis protocols and by using sustainable precursor components, where applicable. This ethical and cost‐effective idea has been set with ongoing universal attempts to use potential waste and bio‐extracts as sustainable alternative carbon sources in the chemical production of different MAX‐phases and MXene compositions. The review has strived to present a roadmap ahead, designing new interdisciplinary investigations by merging different sciences to expand this technology towards maximizing their biocompatibility for human health and ecosystems, and the field's scope for enhanced ethical considerations and product developments, impacting the future of modern agriculture with rational nanotechnology. In the following section, we also discussed some of the important agricultural regulatory aspects of current practices and key considerations for future implementation of potential nanomaterials.

### 4.1 Current Nanotoxicological Risks Associated with Their Nano‐Safety in Real‐World Agricultural Adoption

Beyond the discussed data on biocompatibility assessments of MXenes/MBenes, studying the environmental fate of these nanomaterials and in‐depth analysis of their mobility, accumulation, and interaction with rhizosphere microorganisms in soil systems is essential. This would support the feasibility of MXenes/MBenes’ treatments for real‐world agricultural applications. Indeed, nano‐toxicological risks associated with direct or indirect interaction with MXenes/MBenes can better address the central issue of “nano‐safety” in agricultural adoption. This aspect has so far received little attention, including a recent study by Liu et al.^[^ ^107^ ^]^ and Zhou et al.,^[^ ^112^ ^]^ and due to its importance, further studies are required.

The work by Liu et al. suggested that Ti_3_C_2_T_x_ MXenes exhibit relatively low toxicity toward beneficial soil microbes when applied via root irrigation. High‐throughput sequencing analysis demonstrated that while the application of Ti_3_C_2_T_x_ dispersions may induce transient fluctuations in rhizosphere microbial diversity and community composition, these effects were not statistically significant and reverted to baseline within a few days, suggesting that moderate doses of this MXene have negligible long‐term impacts on rhizosphere microbial dynamics. The work by Zhou et al., has shown that the mobility and accumulation of Ti_3_C_2_T_x_ MXene in soil environments are strongly governed by its surface charge characteristics and oxidation degree. Specifically, interactions with natural organic matter and soil colloidal particles play a key role. In this regard, they reported that humic acid coatings could effectively enhance MXene dispersion stability through electrostatic repulsion, while oxidative surface transformations accelerate degradation and decrease aggregation.

The authors have revealed that Ti_3_C_2_T_x_ surfaces undergo bond formation with hydroxyl and carboxyl groups (e.g., Ti−O−C and Si−O−Ti bonds), increasing surface reactivity and promoting more rapid degradation.^[^ ^112^ ^]^ In contrast, native soil systems, with comparatively low concentrations of these functional groups, exhibited minimal reactivity with Ti_3_C_2_T_x_, limiting degradation. This suggests that MXenes are unlikely to persist in groundwater over extended periods, given their aqueous colloidal dispersion properties and in situ degradation reactivity with the soil environment. Therefore, as also mentioned in the previous section, this property of MXenes, and probably MBenes, must be further improved by surface modification or our recently‐reported approach of chiral‐engineering.^[^ ^66^ ^]^ It is, thereby, speculated that modifying MXenes/MBenes with chiral‐active sources and inducing stable chirality into their structure can remarkably improve their biocompatibility with soil and surrounding ecosystems. The rationale behind chiral engineering of nanomaterials has been described and observed in our previous experiment, where direct soil treatment of plants with chiral MXene did not cause any significant effect on plant overall health and growth. This hypothesis needs to be tested to understand the effects of modified materials on soil colloids and overall soil health.

Moreover, given the increasing importance of water management in agriculture, it would be worthwhile to explore whether MXenes/MBenes can act as sorption‐enhancing agents to help retain water in the rhizosphere. Indeed, due to their unique surface chemistry and lamellar structures, it is likely that these nanosheets can absorb water and slowly release, delivering water molecules to the roots to enhance plant resilience to heat/drought stress and avoid prompt dehydration. Indeed, water retention, sorption, and pH modulation are exciting topics to be further explored for different MXenes/MBenes. This property is anticipated to be highly beneficial in preventing or alleviating root rot diseases by modulating humidity at the root interface. This could open an applicable avenue of studies with the aim of adjusting the soil local pH for optimal nutrient uptake.

The proposed scenario can be further justified with this phenomenon that compared to the polymeric‐based hydrogels (which are potential candidate for agriculture), MXene/MBene‐based hydrogels may offer enhanced water retention due to their strong hydrogen bonds with water molecules.^[^ ^113^ ^]^ High water flux and ion sieving capabilities of MXene/MBene may further promote these interactions to control moisture transport.^[^ ^113^, ^114^ ^]^ Hence, treating pant/soil with MXene/MBene nanosheet‐infused thin layer hydrogels could effectively serve as permeable water buffers and induce the moisture retention during irrigation and gradually release it during the dry seasons. Additionally, the emerging MXene aerogels with dual absorption‐adsorption network structures may efficiently capture and store atmospheric moisture, in comparison to conventional water harvester systems, which usually suffer from a weak water capture ability and poor recyclability. These findings hint at MXenes’ (and probably MBenes) potential to manage moisture and create a localized water‐retaining microenvironment for future agricultural applications.^[^ ^113^, ^114^, ^115^ ^]^

Together, these mechanisms and MXenes’/MBenes’ tunable surface properties and lamellar nanostructures suggest their feasibility as next‐generation sorption‐enhancing agents in agricultural and environmental applications. They can readily and effectively act as water reservoirs in soil matrices, gradually release water to the roots, regulate humidity around the root and at the root‐soil interface to minimize the risk of root rot, enhance the nutrient uptake of the rhizosphere through localize pH buffering. Their abundant surface functional groups, such as –OH, –O, and –F, are expected to enhance the interactions with the protons or hydroxide ions, providing surface protonation‐deprotonation buffering and regulate local pH imbalance due to the fertilizer overuse, microbial activity of irrigation water alkalinity. These pH adjustments near root surfaces could keep nutrients like phosphorus, iron, and manganese in soluble forms, prevent their precipitation, and therefore improve the uptake efficiency by the rhizosphere.^[^ ^114^, ^115^, ^116^ ^]^

### 4.2 Current Regulatory Aspects of Nanomaterials in Agri/Feed/Food in Europe

In Europe, horizontal and sector‐specific legislations provide a framework for the agricultural and food manufacturers and importers, as well as the users, to ensure the quality and safety of market products and substances. It also covers the regulations and policies for nanomaterials and nano‐enabled agricultural/food productions.  As also pointed out in the abstract and introduction, the need of adapting these regulatory provisions to nano‐enabled technologies has been considered with careful regulations and amendments in certain cases or region‐dependent conditions. Figures S28–S30 (Supporting Information) represent some of these aspects, where plot and tabulated regulations are adapted from a recent related report in the literature.^[^ ^117^ ^]^ It gives an overview of some of the current European legislation and regulatory aspects. To some extent, partial regulations regarding the nanomaterials have been explicitly included, where this detailed information is publicly accessible (http://EUR‐LEX.europa.eu/).^[^ ^117^ ^]^

Nanomaterials are explicitly or implicitly covered by different legislations and associated sub‐regulations. Currently, these legislations addressing nanomaterials include the “Regulation on the Provision of Food Information to Consumers (1169/20119)”, “Regulation on Plastic Food Contact Materials and Articles (10/2011)”, “Regulation on Active and Intelligent Materials and Articles (450/2009)”, “Biocidal Products Regulation (528/2012)”, and “Cosmetic Products Regulation (1223/2009)”. There have also been other pieces of focused regulations, including the “Novel Food Regulation (258/97)”: European Commission 2013), and “Annexes to the REACH (Registration, Evaluation, Authorisation and Restriction of Chemicals) Regulation (1907/2006)”: The European Commission 2012).^[^ ^117^, ^118^ ^]^

### 4.3 Legal Act Considerations for Chemical Substances and Biocides Risk Assessments

There have been specific considerations in Europe for biocidal products regulation, such as the “BPR (EU) No 528/2012”, “V. Amenta et al.: Regulatory Toxicology and Pharmacology 73 (2015) 463e476 467”, and “European Parliament and Council 2012”. These regulations specify the provisions for the use of non‐agricultural pesticides/insecticides by both professional product users and consumers. In particular, several different biocidal product types are included (fully described in Annex V of the BPR).^[^ ^117^, ^118^ ^]^ For instance, insect repellents, disinfectants, or chemicals, such as anti‐fouling paints for the ship industries, and preservative materials. One of the most common biocidal nanomaterials is silver‐based nano‐compounds, which are largely utilized for antimicrobial applications by release of Ag ions in a dose‐ and particle size‐dependent manner. Comparable to current pesticides, biocidal‐active substances or derived materials must undergo certain authorization procedures, followed by the “European Parliament and Council, 2009a” or “MS‐Level” for specific provisions. It is notable that approval for active substances for biocidal uses is not necessarily applicable to nanomaterials, except where it has been explicitly mentioned. Thus, extensive protocols and mandatory regulations shall be followed for approval of that specific nanomaterial or nano‐active substance authorization. The “EC Regulation: No 1907/2006”: Registration, Evaluation, Authorisation and Restriction of Chemicals (REACH) (European Parliament and Council 2006) has aimed to define the protection and improve human health and the environment from the associated risks by uncontrolled or non‐suitable use of harmful chemicals or synthetic substances.^[^ ^117^, ^118^ ^]^

In the case of food industries, multiple streams of regulation have been registered under the “REACH”. Importantly, the related substances or materials for application as plant protectors, growth boosters, or biocides are also regarded as defined. In particular, nanomaterials that are in direct or indirect contact with food materials (such as titanium dioxide, nano‐silver, etc.) or in different industrial sectors, such as paint products, are also not exempt from the defined registration under the “REACH” regulations. It is important to note that the “REACH” does not currently and explicitly consider nanomaterials in the legal text regulations; however, it addresses a variety of chemicals. Specifically, in whatever physical shape, form, or size, and as such, its provisions in certain conditions apply to determined nanomaterials. These Annexes Regulations have been reported to be currently under update or revision to more specifically address certain nanomaterials, according to the “European Commission 2012”. Hence, nanomaterials, like other substances, must be determined and classified for specific hazards and toxicity according to current regulations on “Classification, Labelling, and Packaging (CLP)” (European Parliament and Council 2008a). Additionally, the products containing any hazardous substances or nanomaterials, depending on their dose limit, must be labelled based on existing protocols. “European Chemicals Agency (ECHA)” is the responsible organization, managing all the “REACH” considerations and regulations associated with particular restriction and/or authorization processes.^[^ ^117^, ^118^ ^]^

### 4.4 Available Current Guidelines for Nanomaterials Risk Assessments

Existing risk assessment guides and test methods are considered for nanomaterials.  However, as of current, some specific aspects, including the process of sample preparation, characterization, safety efficacy, endpoint, long‐term effects, and exposure data in models, require specific consideration for the development of standardized and validated methods (SCENIHR 2007 and OECD 2013a). The European Scientific Committee has defined and applied regulations on “Emerging and Newly Identified Health Risks (SCENIHR)” in a specific case‐by‐case approach, considering the risk assessment of nanomaterials. This guidance includes the application of nanotechnology and nanoscience in the food and feed chain (“EFSA Scientific Committee” 2011). It also covers specific practical approaches for evaluating and considering the potential risks corresponding to these technological aspects in different categories, including food additives, flavorings, enzymes, novel foods, food contact materials, feed additives, and agrochemical pesticides/insecticides/antimicrobials. This guidance has been reported to evaluate based on physicochemical characterization and specifications through testing approaches based on different scenarios, including interactions, degradation, food contact, and transformation of the nanomaterial before and after ingestion of food/feed.  For more details, please see the original guidance or associated references.^[^ ^117^, ^118^ ^]^

Based on this recent study, the “ECHA” has established a scientific expert “Nano‐Materials Working Group” to discuss technical questions and to provide advice in relation to the development and application of nanomaterials under the “REACH” and “CLP”. The information on requirements for biocides is currently pending, along with ongoing review by the “OECD”, for all possible methodologies to identify the necessary actions and implement the changes and regulation consideration for applications of biocompatible nanomaterials. This highlights the importance of further novel research on nanomaterials and improving their physicochemical and biocompatibility properties to the maximum possible levels.

### 4.5 Regulatory Aspects and Considerations in Other Countries/Regions for Nanomaterials Regulations in Agri/Food/Feed

Likewise, several countries around the globe have been actively involved in examining these regulatory frameworks and their appropriateness for managing nanotechnology. These countries consider a variety of regulations and precautionary approaches to ensure the safety of food products and emerging nanotechnologies in the agri/food/feed industries. More details can be found in the referenced publication, where this adapted information has been described and referred to the responsible organizations and regulatory management sectors.^[^ ^117^, ^118^ ^]^ The key legislation and regulations relevant to these online sources are represented, where the adapted tabulation provides this information. It is also concluded that organized collaborations between countries for information exchange are required to further develop universal regulations and improve the safety of agricultural and food products, which have a direct impact on human health and ecosystems.

In summary, while emerging regulatory frameworks have been introduced for future consideration, once necessary safeguards are achieved. Their innovation may also pose precautionary and safety challenges. Nanomaterials with insufficient safety documents or toxicological data may delay their approval. Thus, public concerns regarding environmental and food safety for human can affect their regulations. However, the opportunity to improve the field by providing comprehensive datasets and sustainability improvement of their nano‐applications is urgently needed. High‐efficiency delivery platforms and robust optimization and reproducibility are key criteria in bringing these new technologies closer to practical scenarios. Environmental considerations for reducing chemical inputs and developing greener synthesis methods are another key aspect that can accelerate their progress, which is aligned with the core goals of European and other regions’ regulatory management considerations. Transparent safety assessments, open data, and alignment with Health principles can boost their translation as next‐generation solutions in addressing today's agro‐challenges.^[^ ^117^, ^118^, ^119^, ^120^, ^121^ ^]^

### 4.6 Environmental and Biological Limitations of Currently Existing Agrochemicals

The widespread implementation of synthetic agrochemicals, such as pesticides, herbicides, and chemical‐based fertilizers, has been conducted in agriculture for decades and continues in today's farming. Beyond the cost and concern to human health of agrochemicals, their adverse environmental impacts are a growing challenge. Environmental degradation, biological imbalance, ecosystem, and unsustainability in the long term are among these limits, threatening climate change. Regarding soil degradation and microbial imbalance, several studies have shown that application of soil fertilizers and agrochemical residues can alter the soil's pH, physicochemical properties, and significantly disrupt the environments of soil‐borne microbial communities. The interaction between agrochemicals, soil, and beneficial soil microorganisms also affects key microbial enzyme activities and their biochemical reactions, as well as symbiotic interactions, nutrient absorption, and essential cycling.^[^ ^122^, ^123^ ^]^ These microbial imbalances can weaken the plant's innate immunity and accelerate conditions favorable to harmful and pathogenic organisms. Indeed, pathogenic organisms utilize pesticides as energy and nutrition sources to enhance their growth and population.^[^ ^124^, ^125^ ^]^

With respect to bioaccumulation, leaching, and runoff into the aquatic environment, it should be mentioned that by consumption of agrochemicals on a large scale, high quantities of the applied substances can be uncontrollably moved downward into the ground or streaming water, affecting the non‐target species, including beneficial worms/nematodes or fish and other aquatic organisms. This critical environmental impact of agrochemical overuse has also been reported to affect the ecosystem equilibrium, biochemical, and histological parameters of marine organisms.^[^ ^126^, ^127^ ^]^ In addition, fertilizer runoff causes eutrophication, triggering algal blooms and phytoplankton overgrowth, a subsequent drop in oxygen levels, and endangering aquatic ecosystems. It is important to mention here that the application of agrochemicals is essential. However, only a low percentage of applied pesticides can efficiently reach their intended biological target; the rest may be lost to volatilization, photodegradation, and particularly leaching and runoff. Runoff from agricultural fields carries significant loads of fertilizers and pesticides into nearby water bodies, leading to eutrophication and toxicity to aquatic organisms, and environmental and ecosystem concerns.^[^ ^122^, ^123^, ^124^, ^125^, ^126^ ^]^ This highlights the application of large‐surface‐area biomaterials for dual‐mode or combinational synergistic applications and controlled/sustained release of agrochemicals, reducing their usage amounts.

Moreover, the evolution of resistance in pests and pathogens is another challenge for using high amounts of agrochemicals. Indeed, pests and pathogens can continuously adapt and evolve to chemical fertilizers, particularly when the same modes of action are used repeatedly. The resistance development in pests is through multiple mechanisms, including enhanced pest metabolic activity, which can lead to faster detoxification of the agrochemical, adaptation, and physiological modification, or behavioral alteration, such as avoiding areas where the chemicals have been applied. This adaptation and evolution subsequently cause serious ecological and economic concerns.^[^ ^122^, ^123^, ^124^, ^125^, ^126^, ^127^, ^128^, ^129^, ^130^ ^]^ Resistance trends are escalating due to insufficient application strategies and pest management (IPM) adaptation, a lack of innovation in innovative, eco‐friendly, and sustainable active compounds. The health implications of conventional agrochemicals are classified from acute to chronic conditions depending on the severity of the impact, as reported in the original study.^[^ ^122^ ^]^ This information has been classified and expanded in **Table** **3**. More details can be found in Figures S31 and S32 (Supporting Information), where the adapted tables are represented.

**Table 3:** Key comparison of environmental/biological limitations of conventional agrochemicals versus today's nanomaterials, particularly MXenes and MBenes.

Parameter/factor	Conventional agrochemicals	Reference	Proposed MXenes/MBenes‐based approaches
Efficiency	Often require repeated application, low targeted delivery	[117, 118]	Their high surface area improves efficiency, enables controlled release, and allows for precision dosing. As a result, these nanosheets are effective at relatively low working doses, even providing effects with a single treatment in one‐pot applications.
Soil health and ecosystem impact	Can alter soil structure and composition; disrupt microbial activity communities	[117, 118]	Potential for minimal disruption; ongoing research needed to check the impact of MXenes/MBenes on soil quality and beneficial organisms.
Water contamination	effect on non‐target aquatic species and high risk to the aquatic ecosystem; eutrophication	[118, 123]	Engineered for reduced leaching, meaning less movement of substances into the environment; lower environmental mobility. These nanosheets are inherently hydrophilic, so they attract and hold water molecules. They can efficiently absorb water molecules between their layers. This has dual benefits: filtering out water contamination, including microbes, and preventing water loss from plants or soil during drought or heat stress, consistently. Their hydrogel application—a form that creates a water‐retaining—is an asset.
Pest resistance	Prolonged use leads to evolution and resistant pest/pathogen strains	[127, 128, 129, 130]	Potential to avoid resistance via multimodal mechanisms. MXenes and MBenes are able to behave smartly against different pathogens. This ability may differ with antimicrobial drugs, which often are effective against specific phytopathogens.
Sustainability	Derived from finite resources; large carbon footprint	–	Potential for sustainable synthesis; recyclable carriers. Apart, MXenes are mostly composed of carbon, which is one of the most abundant elements.
Human and animal health	Direct risk of toxicity or indirect through residues in food and the environment	[117, 118]	Early studies have shown lower toxicity; further toxicology studies are required. Extensive data are available in human cells and small mammalian experiments.
Environmental impact	Contributes to greenhouse gases and biodiversity loss	[129, 131]	Potential for lower environmental footprint with proper strategies, surface modification, and dose reduction. It might also benefit from absorbing heavy metals. Meanwhile, environmental accumulation or degradation of agrochemicals in synergetic functions may occur, which needs to be improved by surface modification, forming a soft and highly bioactive layer on MXene/MBenes to reduce toxicity in the long term.
Knowledge Gaps	Few long‐term, holistic risk studies	–	Needs a comprehensive toxicology and ecology evaluation. It is a field that is rapidly evolving and expected to advance rapidly.

## 5 Conclusion and Outlooks

To conclude, the emerging research on the biocompatibility and bioactivity properties of low‐dimensional MXene/MBene biomaterials is a rapidly evolving development in various sectors of nano‐agriculture. The physicochemical and biological characterizations of different MXene and MBene compositions are found to be key factors in determining their multifunctionality across diverse living systems. Recent years have witnessed significant progress in designing and applying MXene/MBene biomaterials for plant protection, targeted delivery of current agrochemicals, immunoengineering, and defense/growth enhancement approaches. Herein, we presented the first focused review on the applications of MXene/MBene‐based sheets, quantum dots, and derived hybrid//hetero‐structures. In particular, we discussed every available study, including our related pilot works and other significant reports in the literature.

Combining knowledge of interdisciplinary fields in nanotechnology, materials science, plant biology, and applied agriculture, this innovative and pilot review comprehensively discusses the known and proposed bioactivity and functional properties of MXenes/MBenes in plant‐based systems and represents their underlying mechanisms in a one‐stop read. It provided rationale directions for future works, aiming to extend the scope and boundaries of the field toward implementation in further studies and real‐world applications, once they have been proven safe for agricultural uses. In particular, it is recommended that the field further advance by exploring the described outlooks. Beyond that, the topics, such as “benchmarking MXene/MBene elicitation against commercial biostimulants”,“field‐trial designs under realistic stress regimes”, “development of degradable MXene/MBene composites”, etc., are existing research as future works. We also highlight the potential of AI‐based applications for optimizing the synthesis parameters and outputs of MXene/MBene biomaterials. In particular, the applications of DFT calculations, precise machine learning, and AI‐based models are considered future work for predicting or optimizing the production inputs and application outputs of MXene and MBene‐based materials.

## Conflict of Interest

The authors declare no conflict of interest.

## Author Contributions

This work was conceptualized, designed, drafted, and reviewed by A.R, A.A, M.B, and S.K. All authors have approved its submission for journal publication.

## Supporting information



Supporting Information
