# Supplementary material for: The First Review on Nano‐Agricultural Applications of MXene and MBene‐Based Materials for Plant‐Immunoengineering, Controlled Protection, and Inducing Biostimulation Mechanisms
Source: Adv Mater. 2025 Oct 16;38(5):e10350. doi: 10.1002/adma.202510350 (PMC12822536; doi:10.1002/adma.202510350)
Supplement: Supplementary file 1 — Supporting Information [file ADMA-38-e10350-s001.docx]

**Electronic Supplementary Information**

**The First Review on Nano-Agricultural Applications of MXene and MBene-Based Materials for Plant-Immunoengineering, Controlled Protection, and Inducing Biostimulation Mechanisms**

Alireza Rafieerad^a,b,c,*^, Ahmad Amiri^d,e^, Maik Böhmer^a^, Soofia Khanahmadi^a,b^

*^a^ Institute for Molecular Biosciences, Johann Wolfgang Goethe Universität, 60438 Frankfurt am Main, Germany*

*^b^ Institute for Biology and Biotechnology of Plants, University of Münster, Schlossplatz 8, 48143 Münster, Germany*

*^c^ Regenerative Medicine Program, Institute of Cardiovascular Sciences, St. Boniface Hospital Research Centre, Department of Physiology and Pathophysiology, Rady Faculty of Health Sciences, University of Manitoba, Winnipeg, Canada*

*^d^ Russell School of Chemical Engineering, University of Tulsa, Tulsa, OK 74104, United States*

*^e^ Department of Mechanical Engineering, The University of Tulsa, OK 74104, United States*

***Content:***

**Supplementary Figures S1 to S32 (**total pages of SI: 32)

***Correspondence:**

Alireza Rafieerad, MSc, PhD

^1^ Institute of Molecular Biosciences, Faculty of Biological Sciences, Goethe University, 60438, Frankfurt am Main, Germany

^2^ Institute for Biology and Biotechnology of Plants, Münster University, Schlossplatz 8, Germany

^3^ Advanced Biomaterials, Nano-Immune Engineering, and Regenerative Nano-Medicine Program

Canada-Italy Tissue Engineering Laboratory (CITEL), ICS, Saint Boniface Albrechtsen Research, Rady Faculty of Health Sciences, University of Manitoba, Winnipeg, R2H 2A6, Manitoba, Canada

Contact E-mail: alireza.rafieerad.formal@gmail.com

**Supplementary Figures S1:**

Representation of the schematic surface modification and structural functionalization of MXenes.

**Supplementary Figures S2**:

Representative illustration of the most recent advances regarding the experimental development and application of 2D boride phases/MBenes through different methods.

**Supplementary Figures S3 to S21:**

Representation of the predicted biocompatibility/toxicity of MXenes/MBenes and related chemical structures with diverse biological and environmental systems.

**Supplementary Figures S22:**

Representation of the proposed features and expected biological impacts of 2D MBenes on living organisms and their envisioned future biotechnological applications.

**Supplementary Figures S23:**

Representation of the data depicting the chlorophyll content measurement from the extracts of *L. sativum*, *S. alba*, and *S. saccharatum* plant treated with MoAlB@MBene.

**Supplementary Figures S24 to 27:**

Representation of the schematic and reported data depicting the interactions and the potential effects of nanomaterials on plants, including positive impacts or adverse effects.

**Supplementary Figures S28 to 30:**

Representation of the schematic and reported data depicting the potential applications of nanotechnology and nanomaterials in agriculture, feed, and food sectors. Overview of European and non-European legal frameworks governing authorization procedures and the proposed nanomaterial provisions for agri/feed/food applications.

**Supplementary Figures S31 and 32:**

Representation of the schematic and reported data depicting a summary of the globally selected insecticides/herbicides: their mode-of-action and proposed adverse health implications.


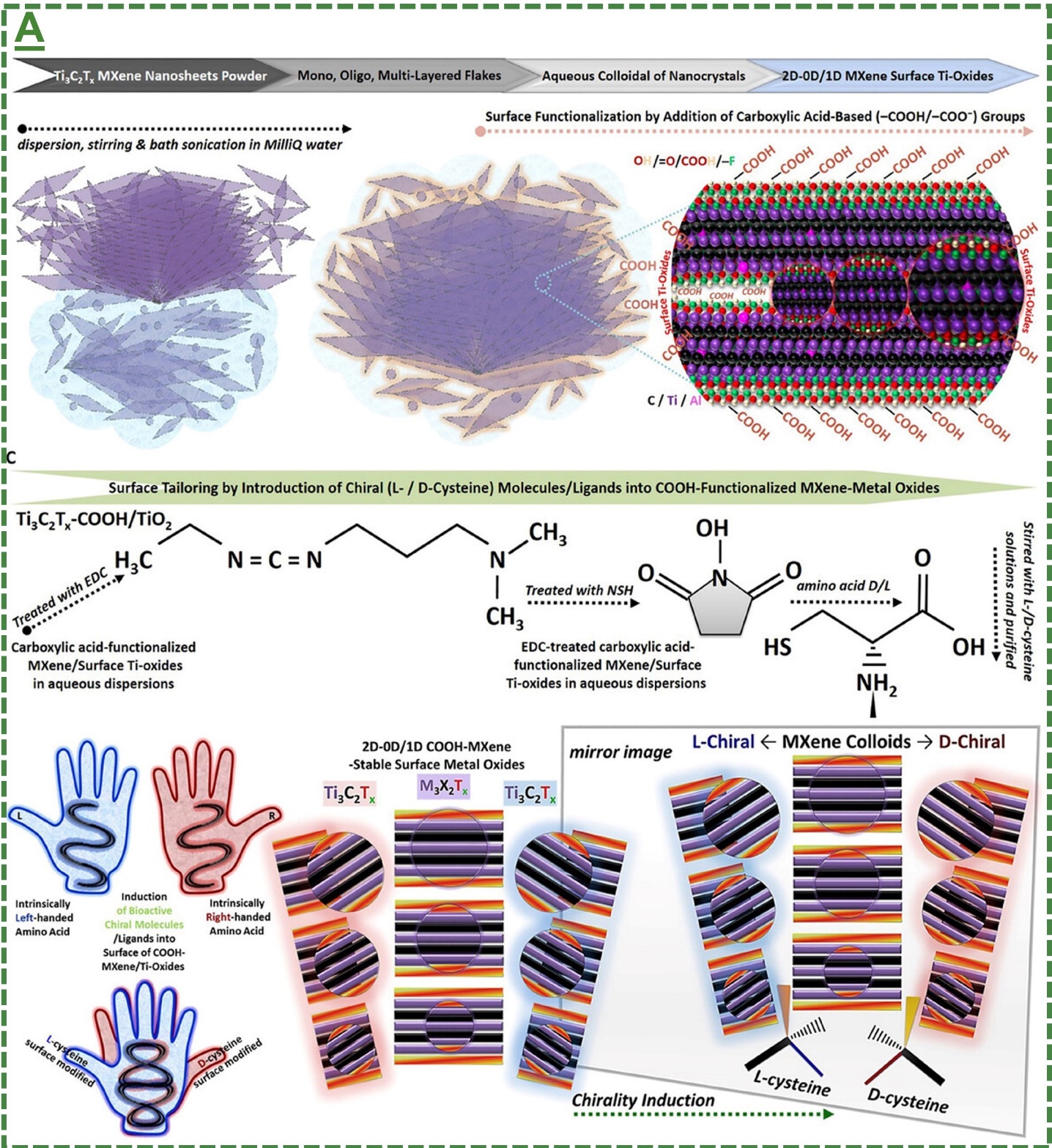


**Supplementary Figure S1:** Schematic illustration of an innovative chiral engineering of MXenes. **A**, The workflow of converting 2D Ti_3_C_2_T_x_ as a representative 2D MXene to stable chiral-engineered right/left-handed asymmetric mixed-low-dimensional heterostructures. The as-designed novel surface modification and structural functionalization method effectively induced chirality into colloidal dispersions of carboxyl-based-modified Ti_3_C_2_T_x_ MXene dispersions using a facile and universal crosslinking method (EDC/NHS, C_8_H_17_N_3_·HCl/C_4_H_5_NO_3_). *The figure is reproduced with permission, Journal copyright,* [*https://doi.org/10.1002/smll.202500654*](https://doi.org/10.1002/smll.202500654).^68 (main text)^


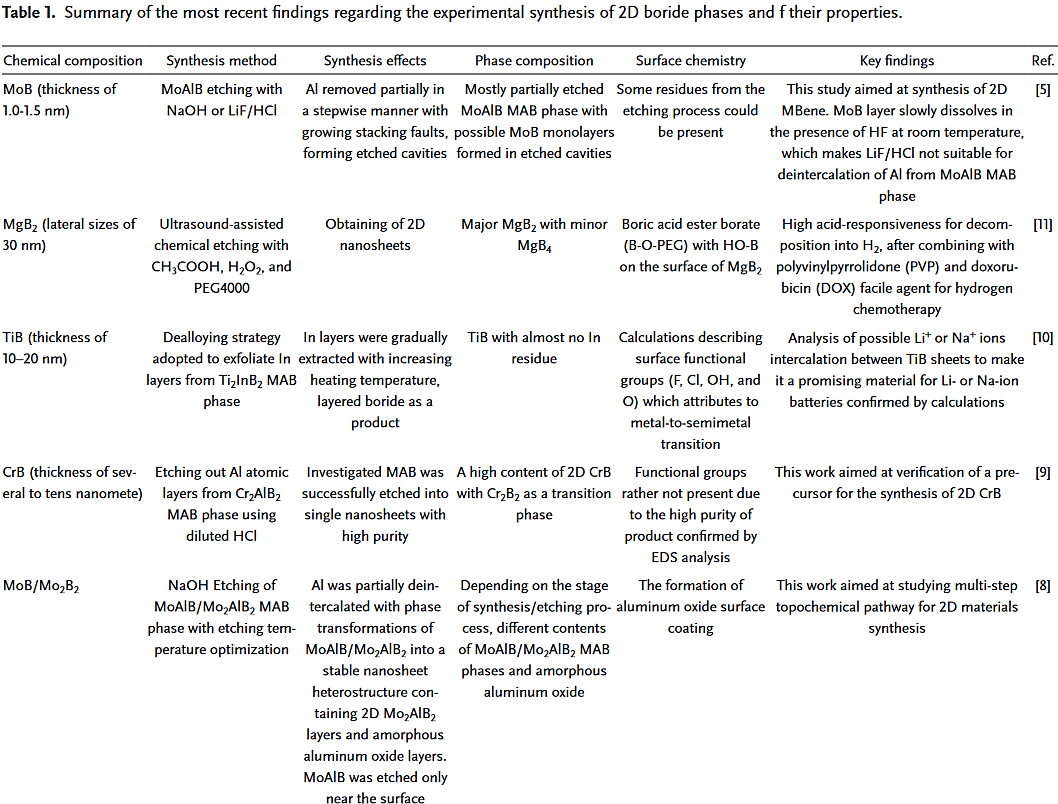


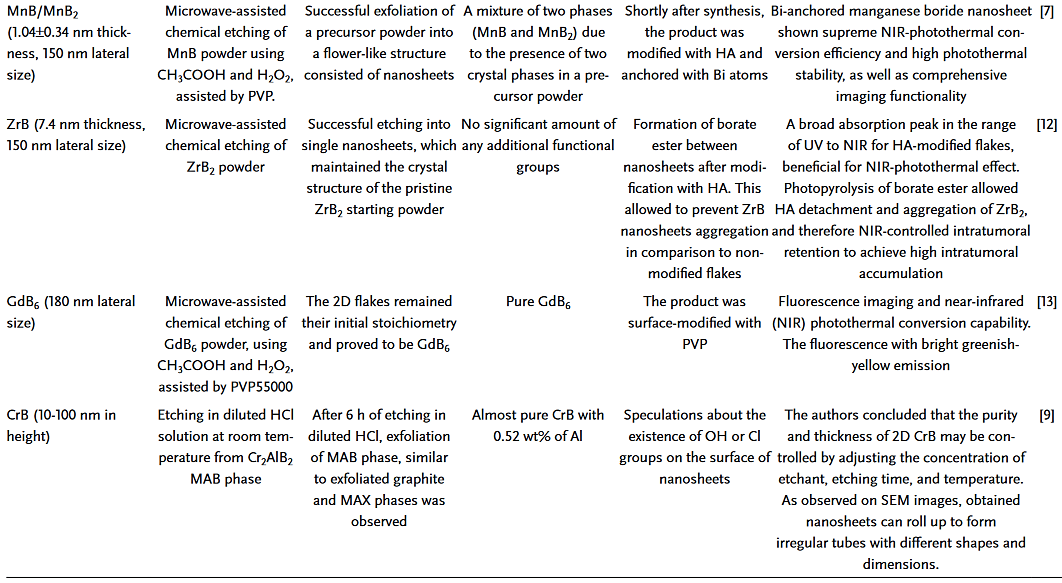


**Supplementary Figure S2:** The adapted summary of the most recent reported advances regarding the experimental development and application of 2D boride phases/MBenes through different methods. This information is adapted from Table 1 of Jakubczak et al. (2021) with permission from the reference, *Journal* *license AFM, Copyright, Wiley*.75 (main text) https://doi.org/10.1002/adfm.202103048


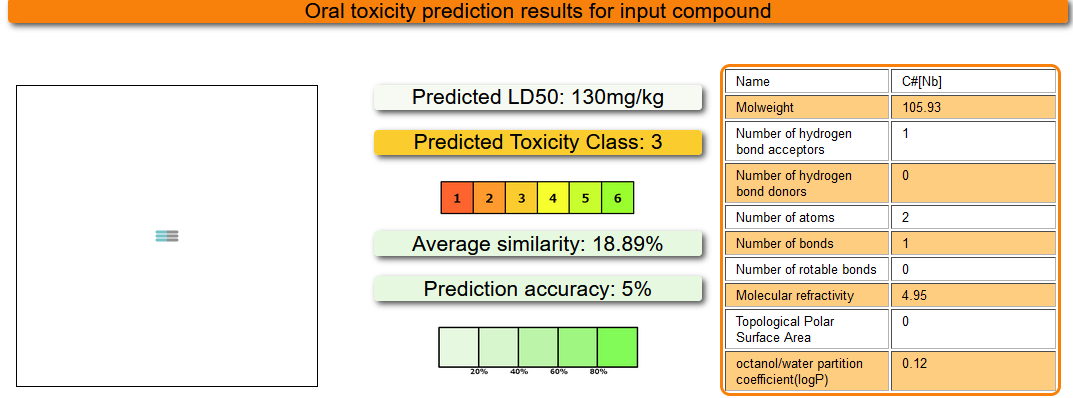


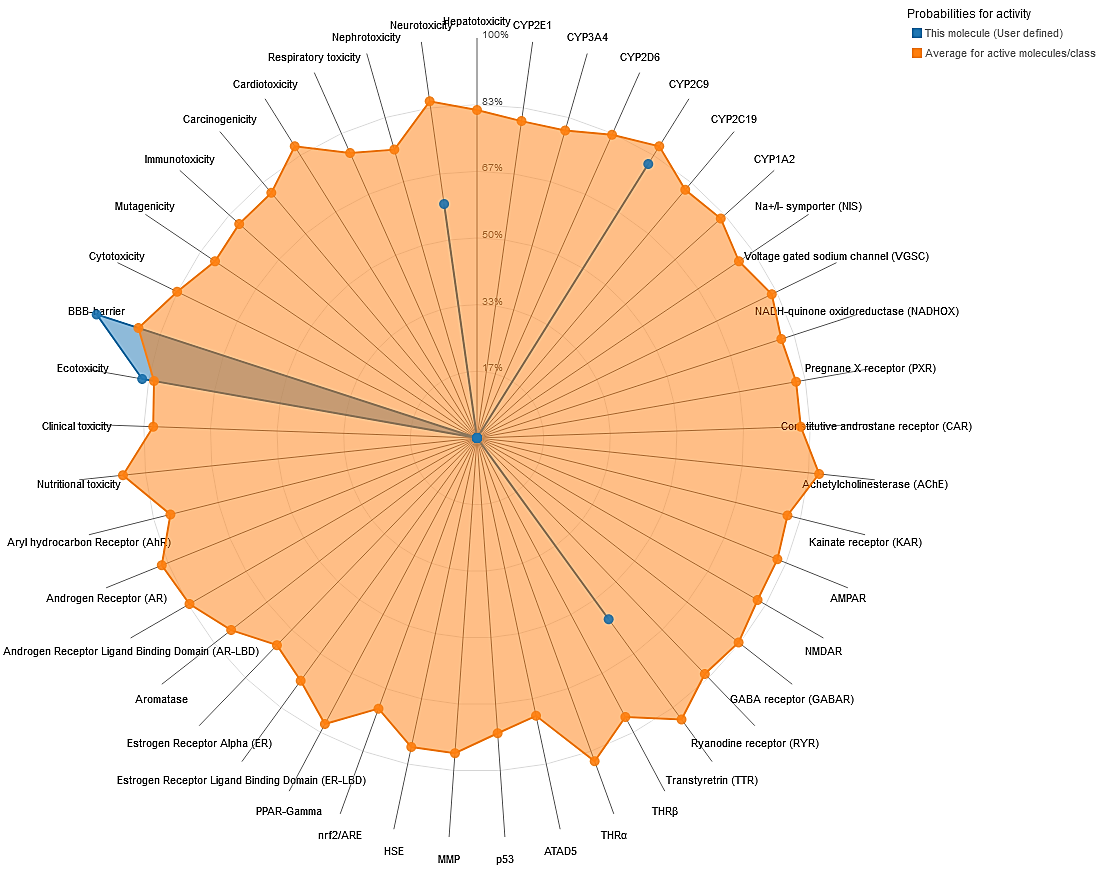


**Supplementary Figure S3:** The predicted biocompatibility/toxicity of niobium carbide, PubChem CID: 4060793, molecular weight: 105.925 g/mol (computed by PubChem 2.2 (PubChem release 2021.10.14)), Molecular Formula: CHNb, SMILES: C#[Nb], <https://pubchem.ncbi.nlm.nih.gov/compound/4060793>. The toxicity radar chart (screen sh.) is intended to illustrate the confidence of positive toxicity predictions with different bio-systems (quick results) of this chemical composition compared to the average of its class. Priyanka Banerjee, Emanuel Kemmler, Mathias Dunkel, Robert Preissner, ProTox 3.0: a webserver for the prediction of toxicity of chemicals, *Nucleic Acids Research*, Volume 52, I W1, 5 J 2024, P W513–W520 <https://tox.charite.de/>.<https://doi.org/10.1093/nar/gkae303> (***predicted LD50: 130 mg kg^−1^, toxicity class: 3***).


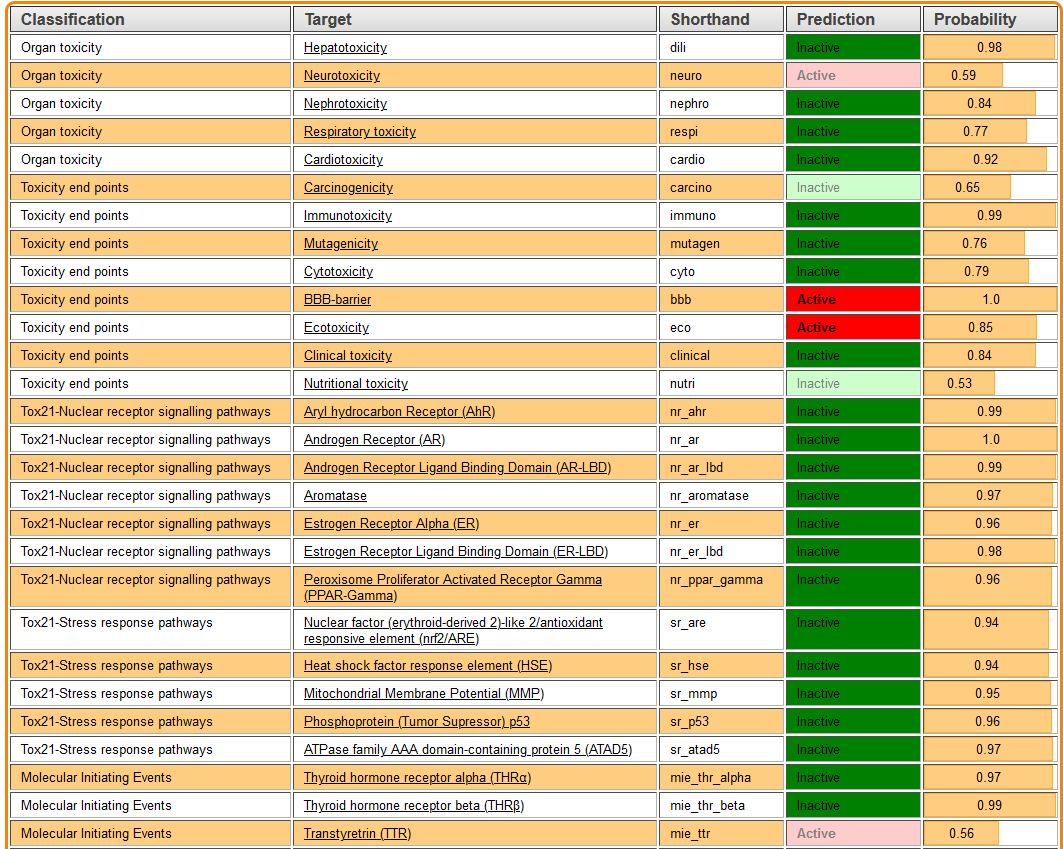


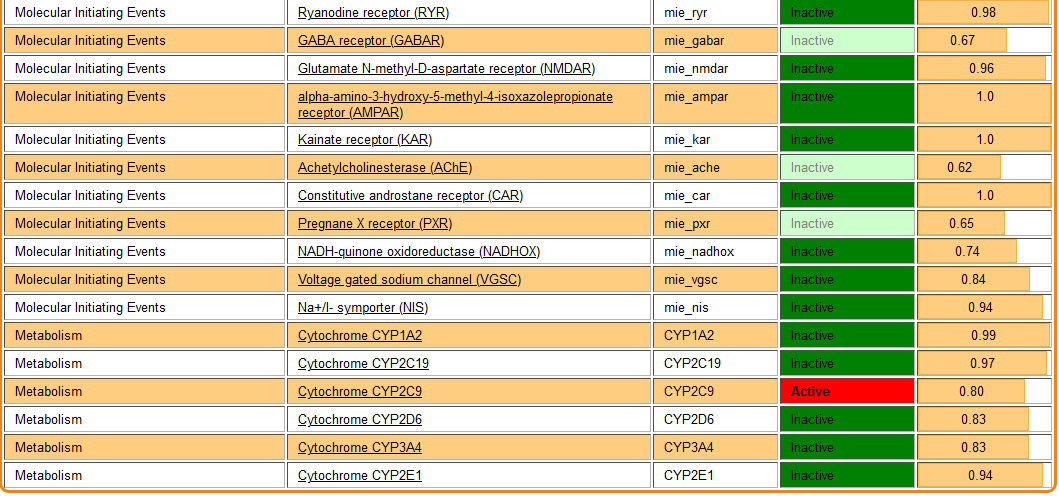


**Supplementary Figure S4:** The biocompatibility/toxicity evaluations (screen sh.) for niobium carbide to illustrate the predicted biocompatibility/toxicity with different biological systems and their probability numbers. Priyanka Banerjee, Kemmler et al, ProTox 3.0: a webserver for the prediction of toxicity of chemicals, *Nucleic Acids Research*, Volume 52, I W1, 2024, P W513–W520 <https://tox.charite.de/>.<https://doi.org/10.1093/nar/gkae303>


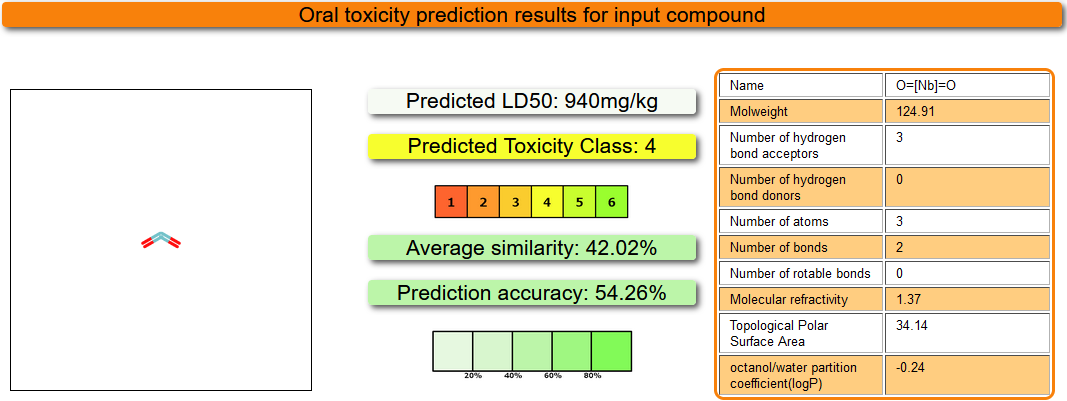


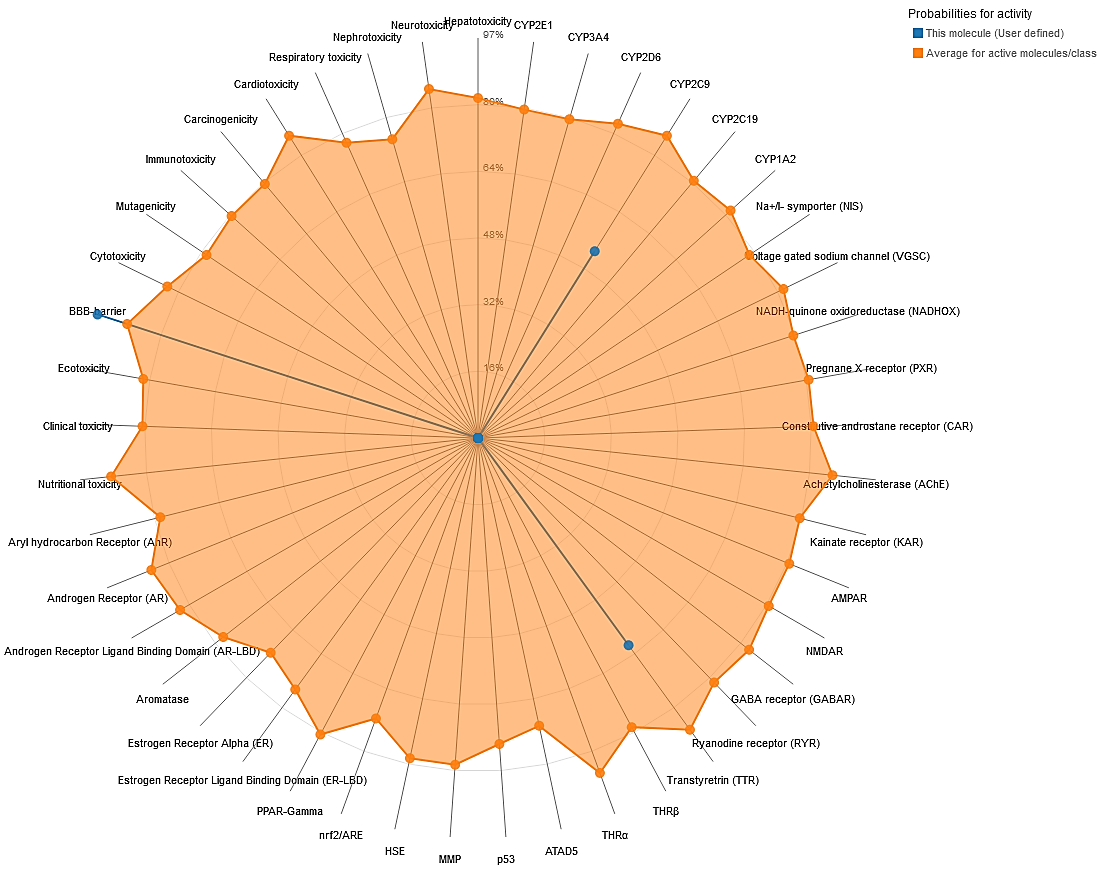


**Supplementary Figure S5:** The predicted biocompatibility/toxicity of niobium dioxide, PubChem CID: 82839, molecular weight: 124.905 g/mol (computed by PubChem 2.2 (PubChem release 2025.04.14)), Molecular Formula: NbO_2_, SMILES: O=[Nb]=O, <https://pubchem.ncbi.nlm.nih.gov/compound/82839>. The toxicity radar chart (screen sh.) is intended to illustrate the confidence of positive toxicity predictions with different bio-systems (quick results) of this chemical composition compared to the average of its class. Priyanka Banerjee, Emanuel Kemmler, Mathias Dunkel, Robert Preissner, ProTox 3.0: a webserver for the prediction of toxicity of chemicals, *Nucleic Acids Research*, Volume 52, I W1, 5 J 2024, P W513–W520 <https://tox.charite.de/>.<https://doi.org/10.1093/nar/gkae303> (***predicted LD50: 940 mg kg^−1^, toxicity class: 4***).


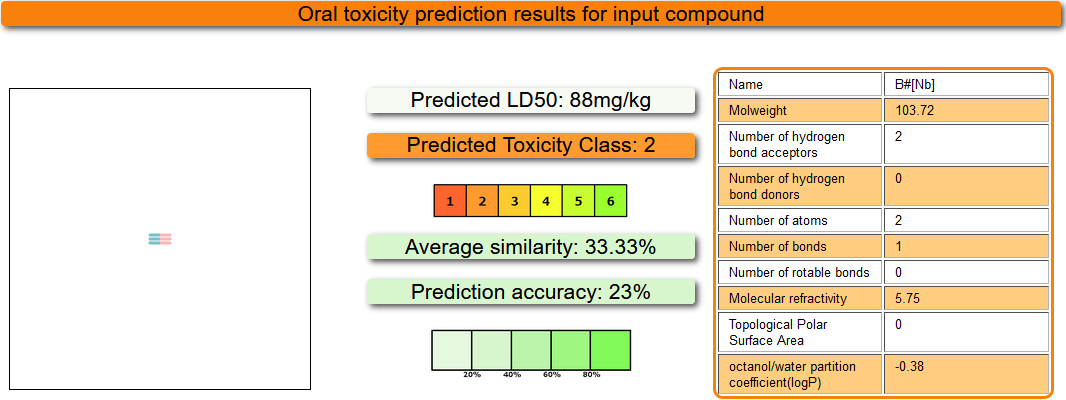


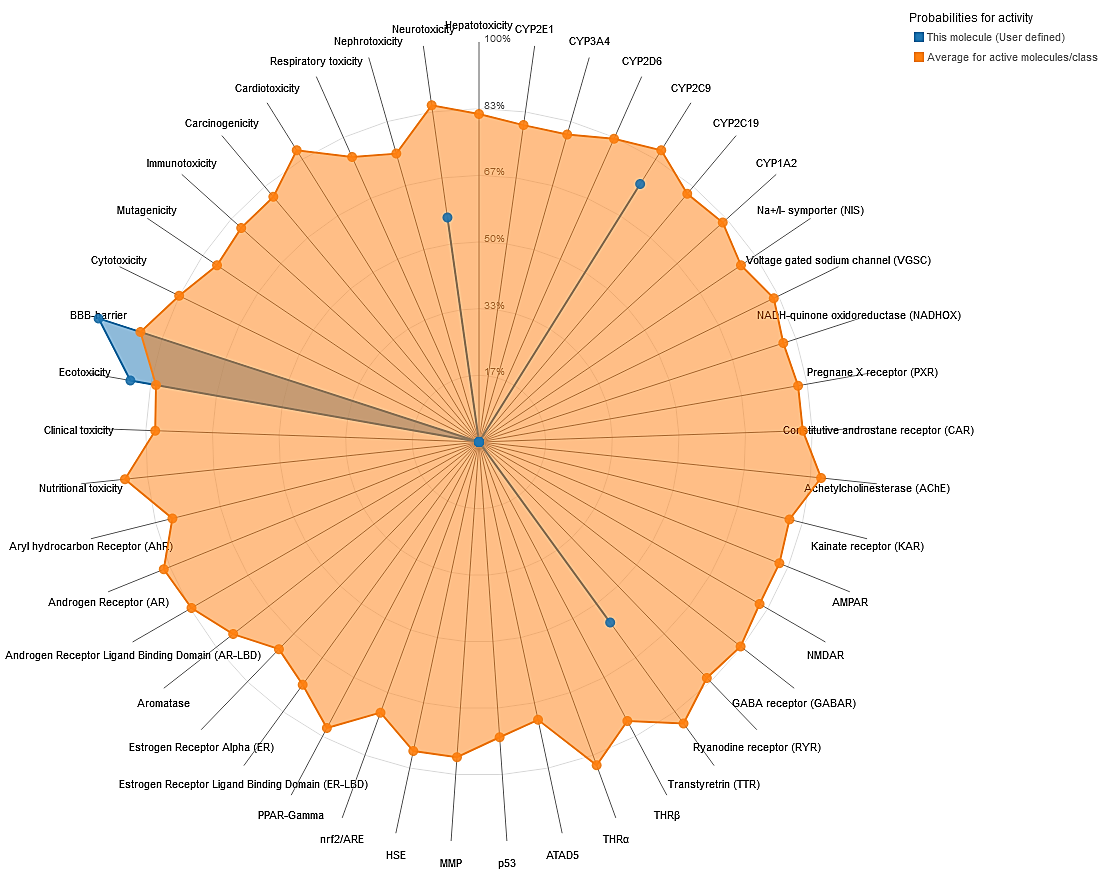


**Supplementary Figure S6:** The predicted biocompatibility/toxicity of niobium boride (NbB), PubChem CID: 82882, molecular weight: 103.72 g/mol (computed by PubChem 2.2 (PubChem release 2025.04.14)), Molecular Formula: BNb, SMILES: B#[Nb], <https://pubchem.ncbi.nlm.nih.gov/compound/82882>. The toxicity radar chart (screen sh.) is intended to illustrate the confidence of positive toxicity predictions with different bio-systems (quick results) of this chemical composition compared to the average of its class. Priyanka Banerjee, Emanuel Kemmler, Mathias Dunkel, Robert Preissner, ProTox 3.0: a webserver for the prediction of toxicity of chemicals, *Nucleic Acids Research*, Volume 52, I W1, 5 J 2024, P W513–W520 <https://tox.charite.de/>.<https://doi.org/10.1093/nar/gkae303> (***predicted LD50: 88 mg kg^−1^, toxicity class: 2***).


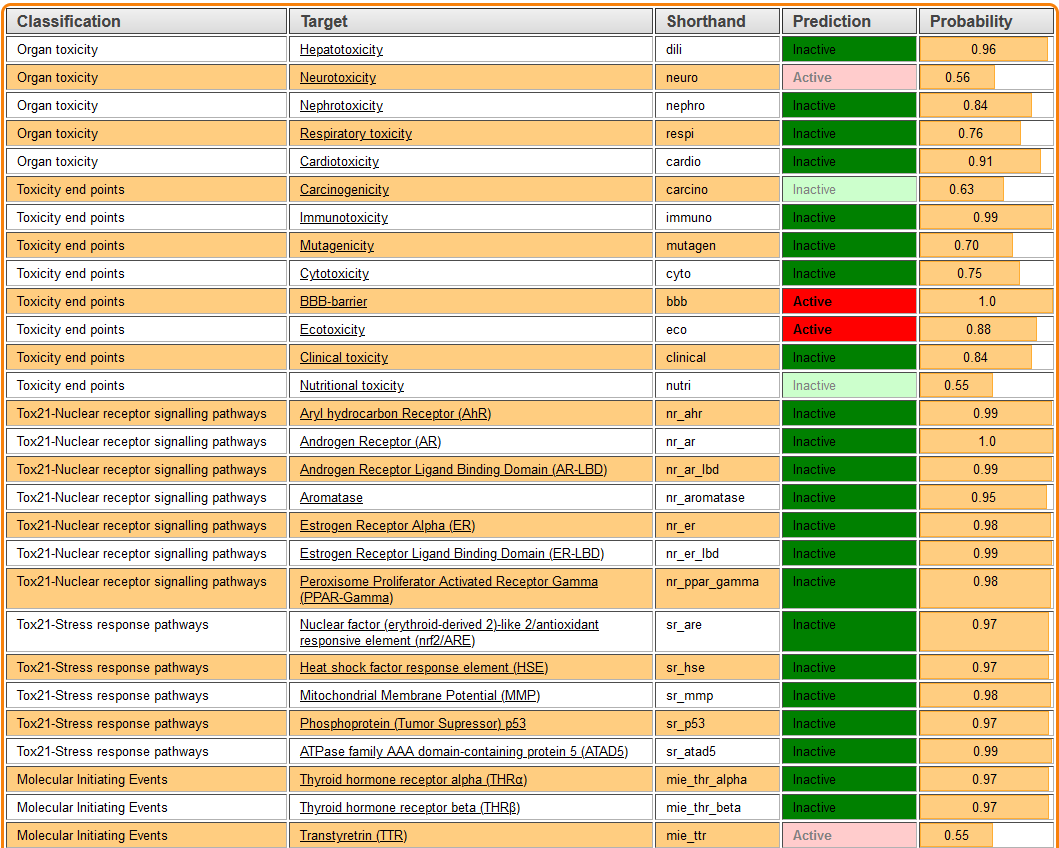


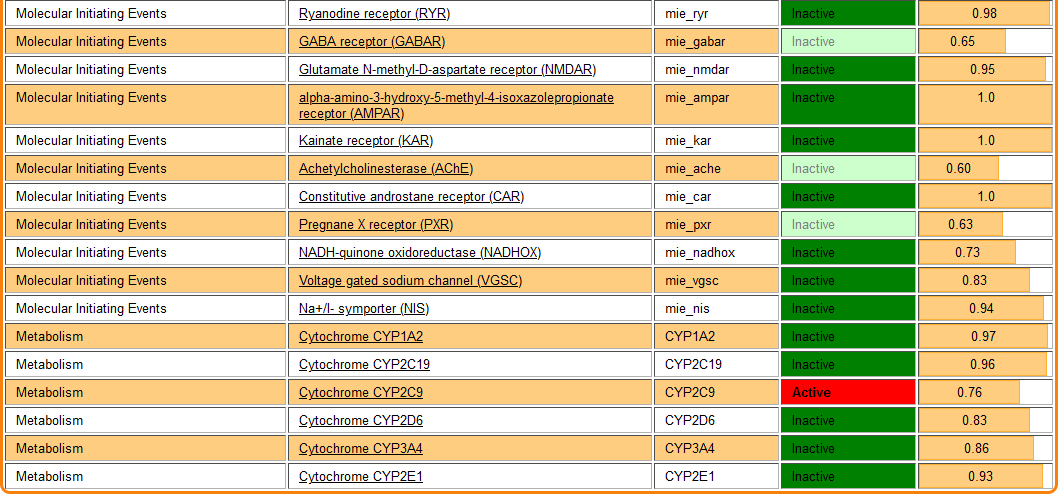


**Supplementary Figure S7:** The biocompatibility/toxicity evaluations (screen sh.) for niobium boride (NbB) to illustrate the predicted biocompatibility/toxicity with different biological systems and their probability numbers. Priyanka Banerjee, Kemmler et al, ProTox 3.0: a webserver for the prediction of toxicity of chemicals, *Nucleic Acids Research*, Volume 52, I W1, 2024, P W513–W520 <https://tox.charite.de/>.<https://doi.org/10.1093/nar/gkae303>


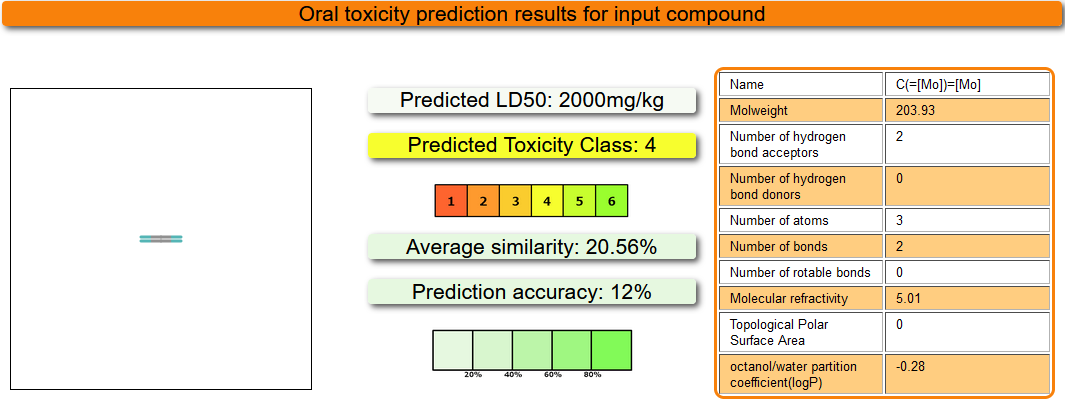


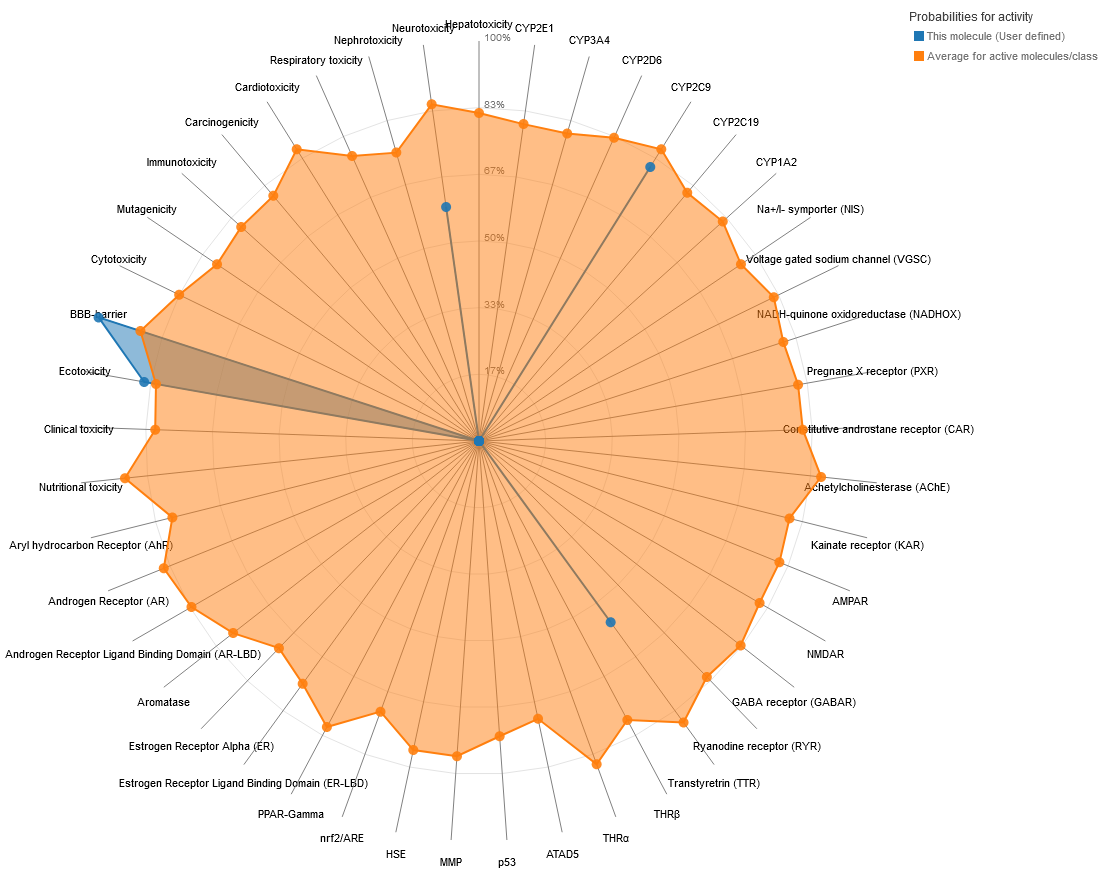


**Supplementary Figure S8:** The predicted biocompatibility/toxicity of Molybd (Mo_2_C MXene monolayer/powder), PubChem CID: 10987348 molecular weight: 203.9 g/mol (computed by PubChem 2.1 (PubChem release 2021.05.07)), molecular formula: CMo_2_ SMILES: C(=[Mo])=[Mo], <https://pubchem.ncbi.nlm.nih.gov/compound/10987348>. The toxicity radar chart (screen) is intended to illustrate the confidence of positive toxicity predictions with different bio-systems (quick results) of this chemical composition compared to the average of its class. Priyanka Banerjee, Emanuel Kemmler, Mathias Dunkel, Robert Preissner, ProTox 3.0: a webserver for the prediction of toxicity of chemicals, *Nucleic Acids Research*, V 52, I W1, 5 J 2024, P W513–W520 <https://tox.charite.de/>.<https://doi.org/10.1093/nar/gkae303> (***predicted LD50: 2000 mg kg^−1^, toxicity class: 4***).


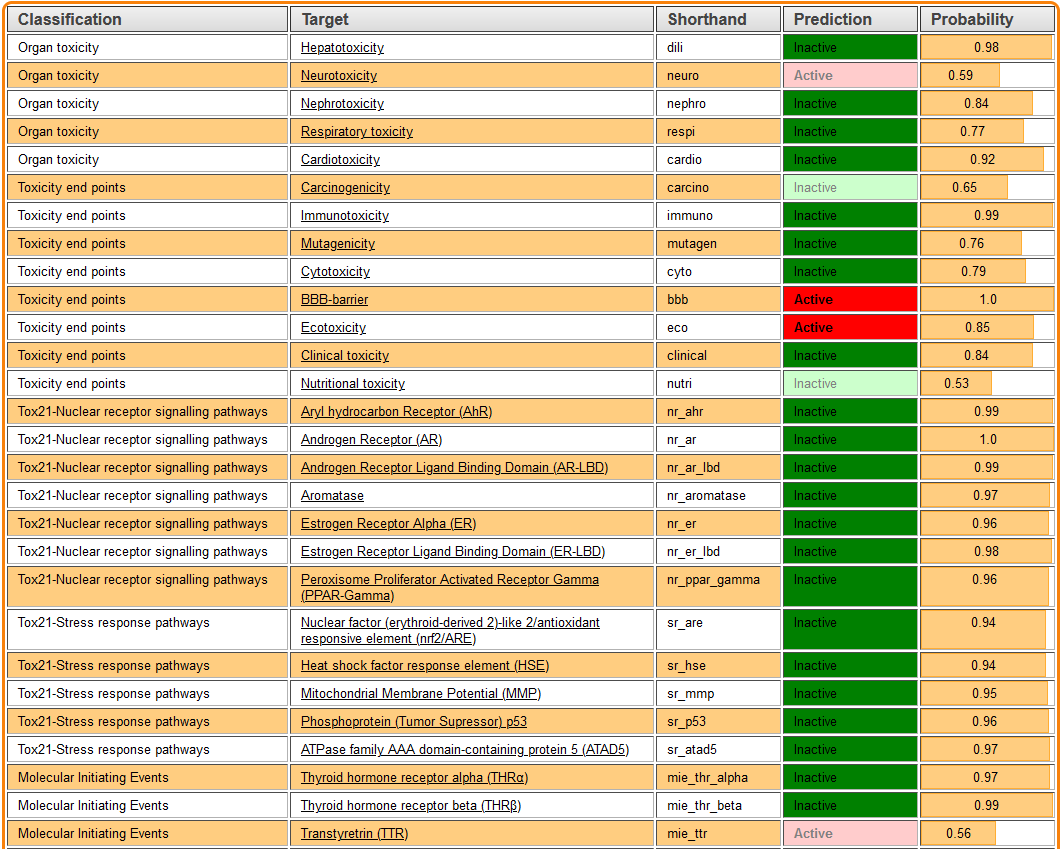


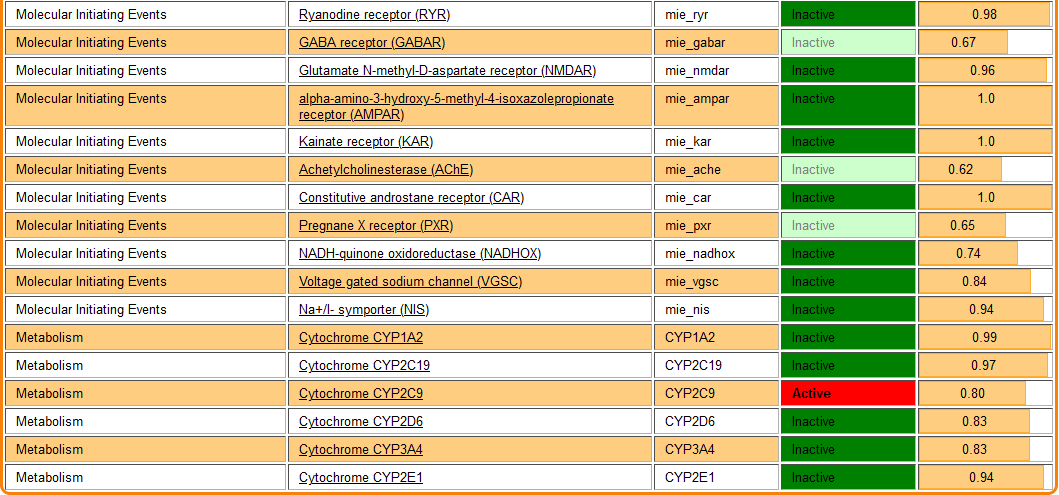


**Supplementary Figure S9:** The biocompatibility/toxicity evaluations (screen) for Molybd (Mo_2_C MXene monolayer/powder) to illustrate the predicted biocompatibility/toxicity with different biological systems and their probability numbers. Priyanka Banerjee, Kemmler et al, ProTox 3.0: a webserver for the prediction of toxicity of chemicals, *Nucleic Acids Research*, Volume 52, I W1, 2024, P W513–W520 <https://tox.charite.de/>.<https://doi.org/10.1093/nar/gkae303>


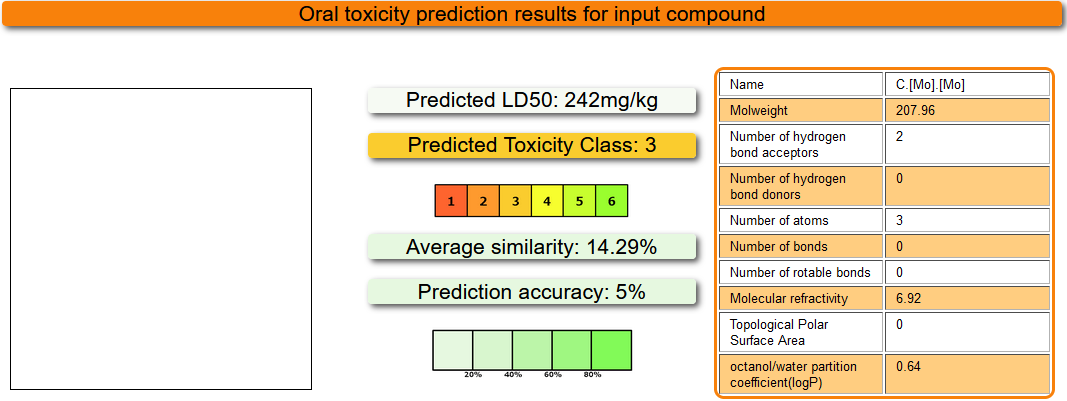


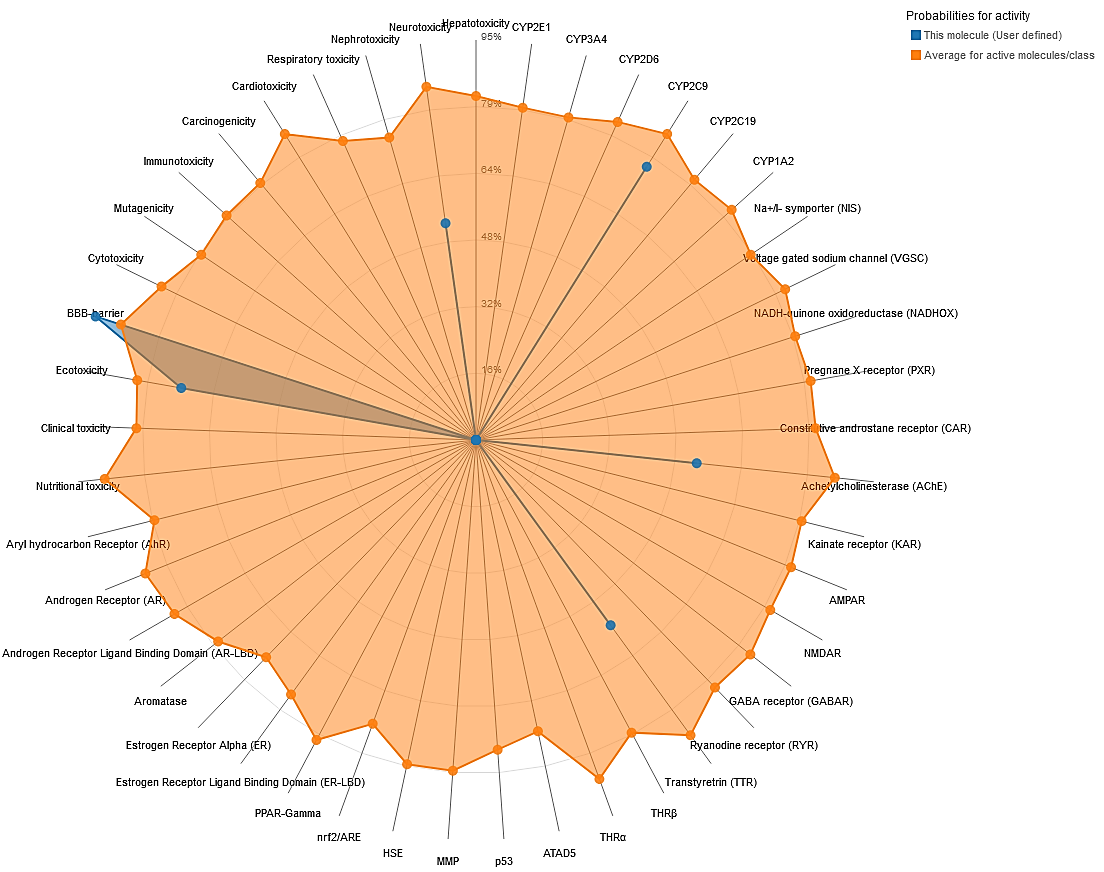


**Supplementary Figure S10:** The predicted biocompatibility/toxicity of molybdenum carbide (Mo_2_C), PubChem CID: 159430, molecular weight: 207.9 g/mol (computed by PubChem 2.2 (PubChem release 2025.04.14)), Molecular Formula: CH_4_Mo_2_, SMILES: C.[Mo].[Mo], <https://pubchem.ncbi.nlm.nih.gov/compound/159430>. The toxicity radar chart (screen) is intended to illustrate the confidence of positive toxicity predictions with different bio-systems (quick results) of this chemical composition compared to the average of its class. Priyanka Banerjee, Emanuel Kemmler, Mathias Dunkel, Robert Preissner, ProTox 3.0: a webserver for the prediction of toxicity of chemicals, *Nucleic Acids Research*, V 52, I W1, 5 J 2024, P W513–W520 <https://tox.charite.de/>.<https://doi.org/10.1093/nar/gkae303> (***predicted LD50: 242 mg kg^−1^, toxicity class: 3***).


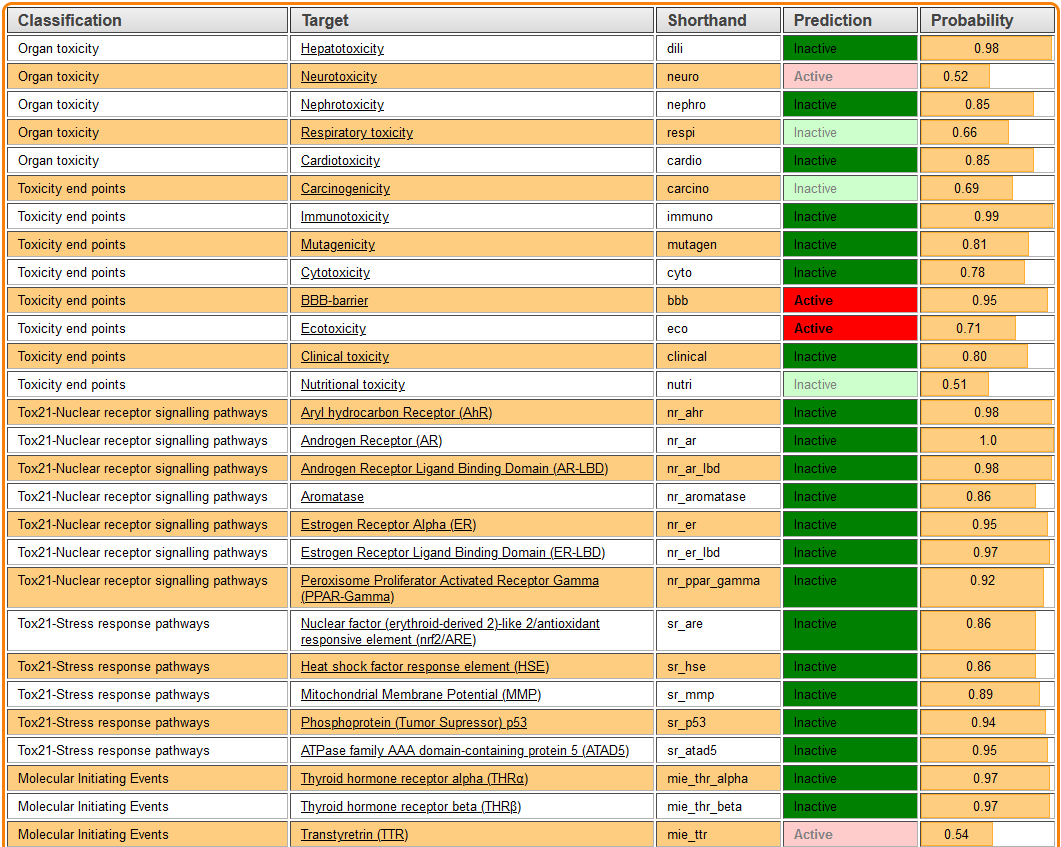


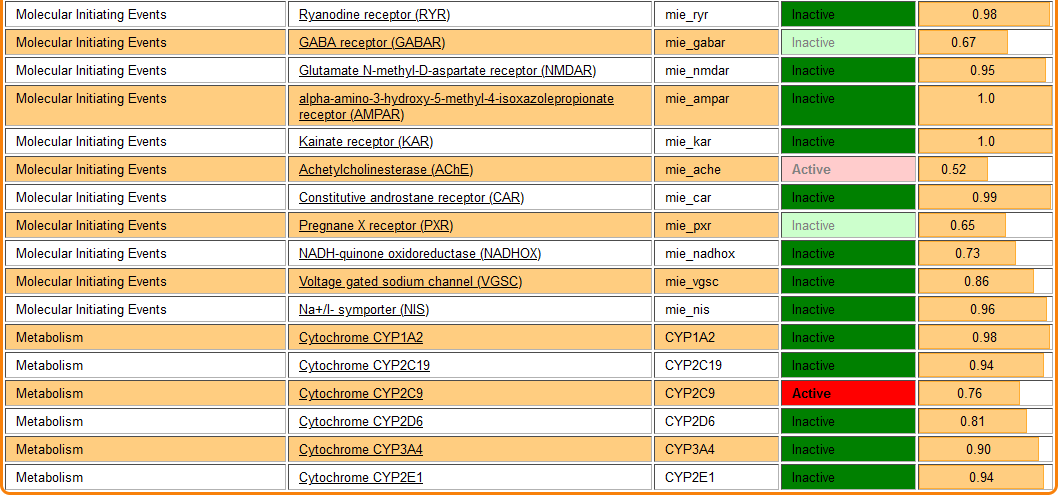


**Supplementary Figure S11:** The biocompatibility/toxicity evaluations (screen sh.) for molybdenum carbide (Mo_2_C) to illustrate the predicted biocompatibility/toxicity with different biological systems and their probability numbers. Priyanka Banerjee, Kemmler et al, ProTox 3.0: a webserver for the prediction of toxicity of chemicals, *Nucleic Acids Research*, Volume 52, I W1, 2024, P W513–W520 <https://tox.charite.de/>.<https://doi.org/10.1093/nar/gkae303>


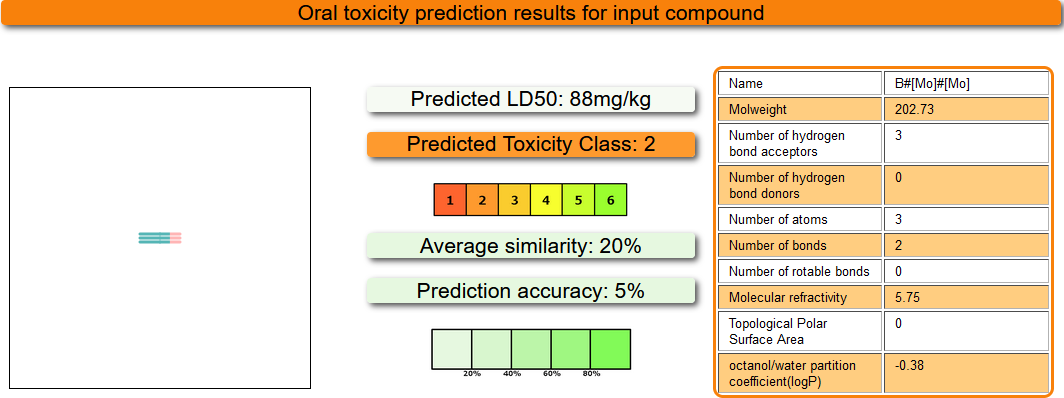


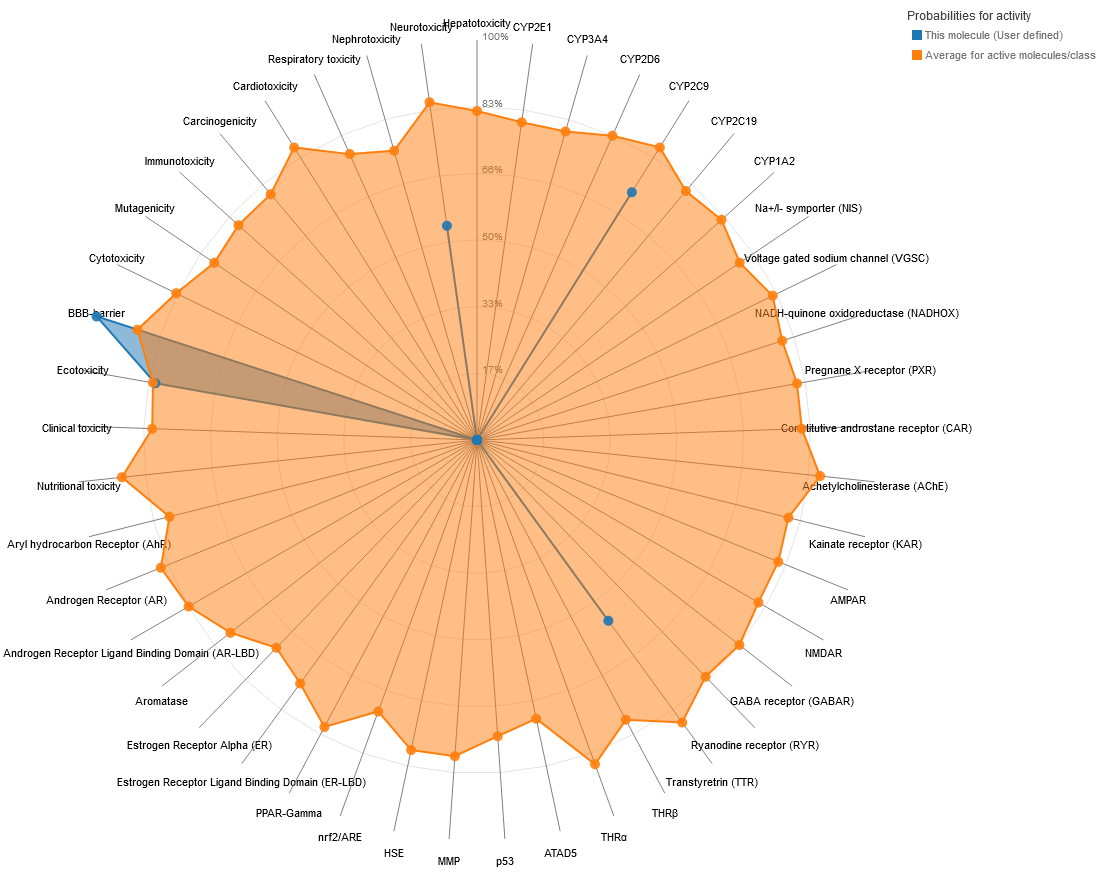


**Supplementary Figure S12:** The predicted biocompatibility/toxicity of molybdenum boride (Mo_2_B), PubChem CID: 72720418, molecular weight: 202.7 g/mol (computed by PubChem 2.2 (PubChem release 2025.04.14)), Molecular Formula: BMo_2_, SMILES: B#[Mo]#[Mo], <https://pubchem.ncbi.nlm.nih.gov/compound/72720418>. The toxicity radar chart (screen sh.) is intended to illustrate the confidence of positive toxicity predictions with different bio-systems (quick results) of this chemical composition compared to the average of its class. Priyanka Banerjee, Emanuel Kemmler, Mathias Dunkel, Robert Preissner, ProTox 3.0: a webserver for the prediction of toxicity of chemicals, *Nucleic Acids Research*, Volume 52, I W1, 5 J 2024, P W513–W520 <https://tox.charite.de/>.<https://doi.org/10.1093/nar/gkae303> (***predicted LD50: 88 mg kg^−1^, toxicity class: 2***).


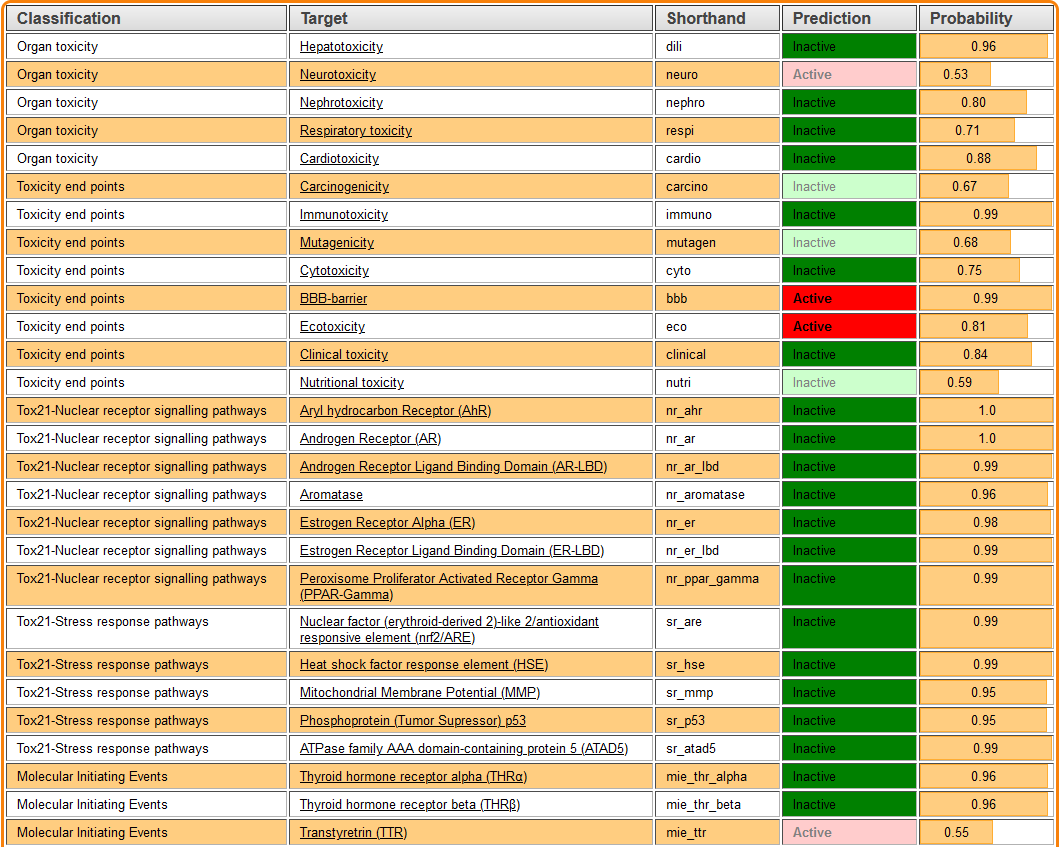


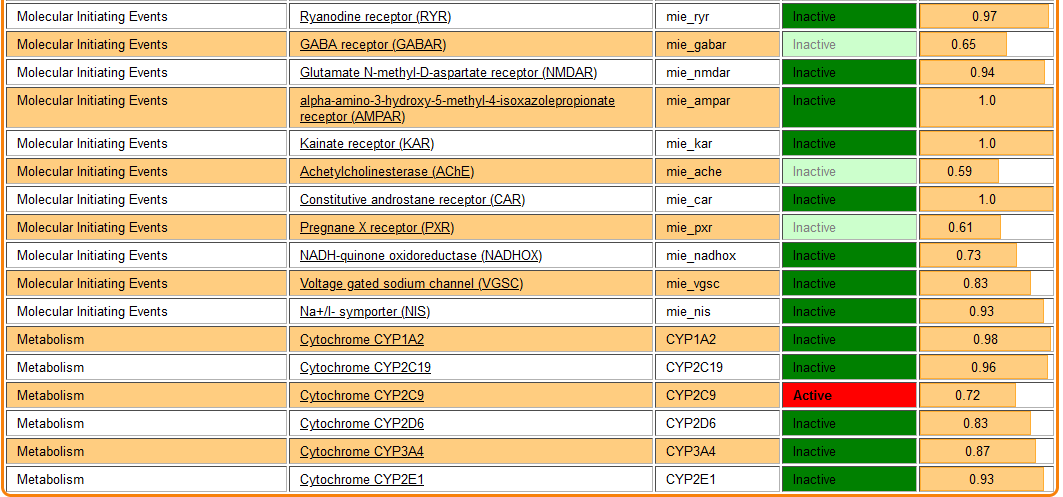


**Supplementary Figure S13:** The biocompatibility/toxicity evaluations (screen sh.) for molybdenum boride (Mo_2_B) to illustrate the predicted biocompatibility/toxicity with different biological systems and their probability numbers. Priyanka Banerjee, Kemmler et al, ProTox 3.0: a webserver for the prediction of toxicity of chemicals, *Nucleic Acids Research*, Volume 52, I W1, 2024, P W513–W520 <https://tox.charite.de/>.<https://doi.org/10.1093/nar/gkae303>


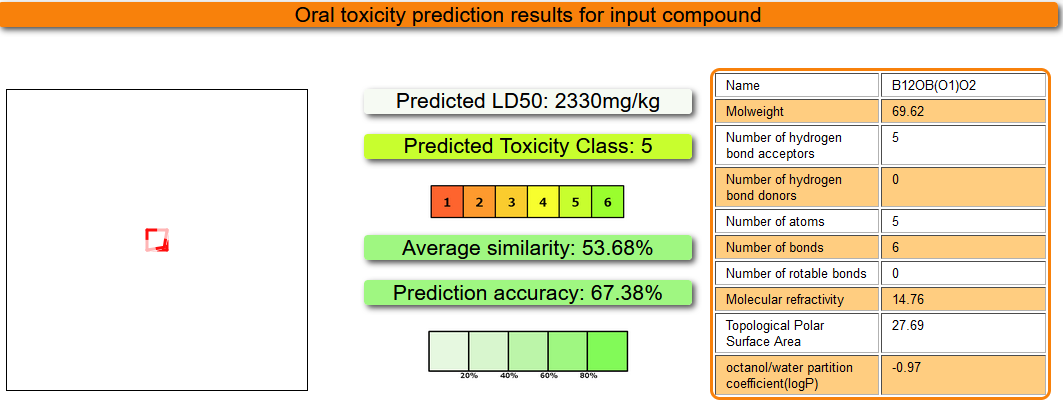


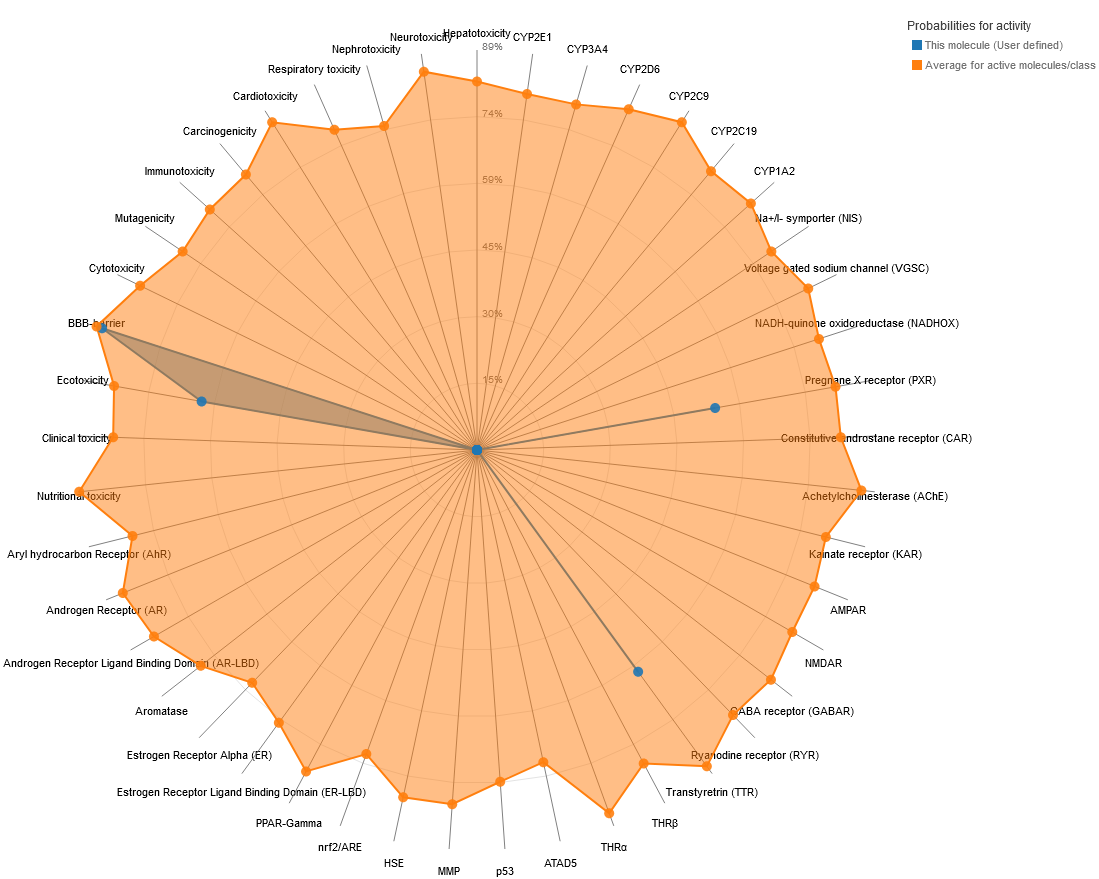


**Supplementary Figure S14:** The predicted biocompatibility/toxicity of boron oxide (B_2_O_3_), PubChem CID: 11073337, molecular weight: 69.63 g/mol (computed by PubChem 2.1 (PubChem release 2021.05.07)), Molecular Formula: B_2_O_3_, SMILES: B12OB(O1)O2, <https://pubchem.ncbi.nlm.nih.gov/compound/11073337>. The toxicity radar chart (screen sh.) is intended to illustrate the confidence of positive toxicity predictions with different bio-systems (quick results) of this chemical composition compared to the average of its class. Priyanka Banerjee, Emanuel Kemmler, Mathias Dunkel, Robert Preissner, ProTox 3.0: a webserver for the prediction of toxicity of chemicals, *Nucleic Acids Research*, Volume 52, I W1, 5 J 2024, P W513–W520 <https://tox.charite.de/>.<https://doi.org/10.1093/nar/gkae303> (***predicted LD50: 2330 mg kg^−1^, toxicity class: 5***).


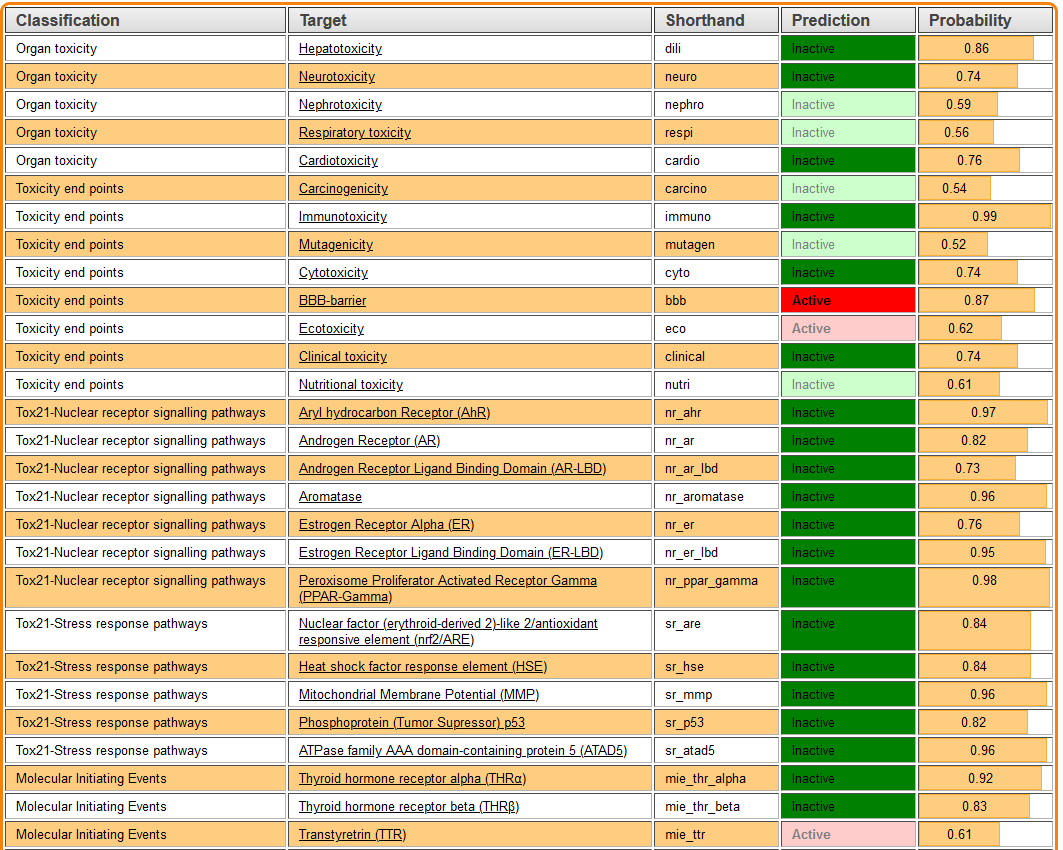


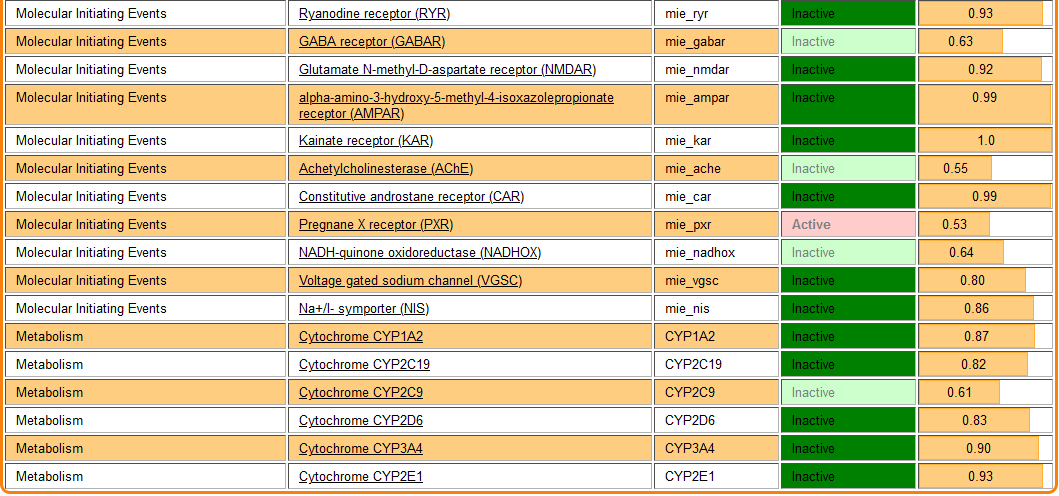


**Supplementary Figure S15:** The biocompatibility/toxicity evaluations (screen sh.) for boron oxide (B_2_O_3_), to illustrate the predicted biocompatibility/toxicity with different biological systems and their probability numbers. Priyanka Banerjee, Kemmler et al, ProTox 3.0: a webserver for the prediction of toxicity of chemicals, *Nucleic Acids Research*, Volume 52, I W1, 2024, P W513–W520 <https://tox.charite.de/>.<https://doi.org/10.1093/nar/gkae303>

**
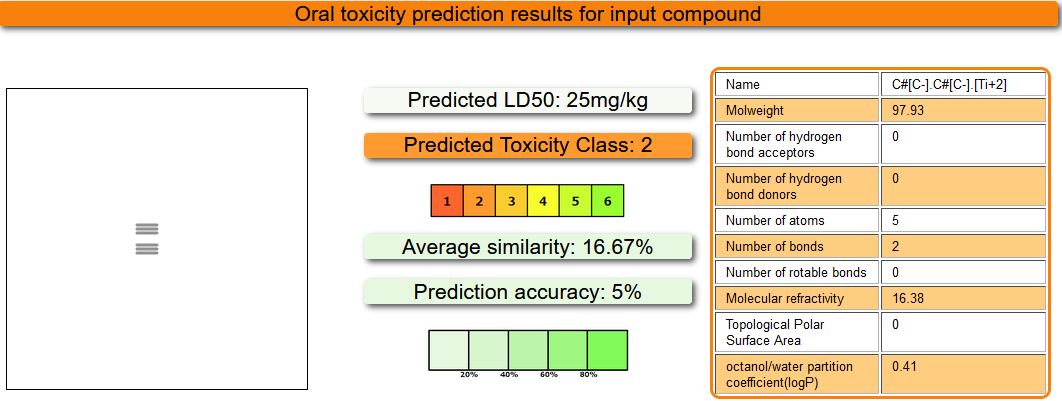
**

**
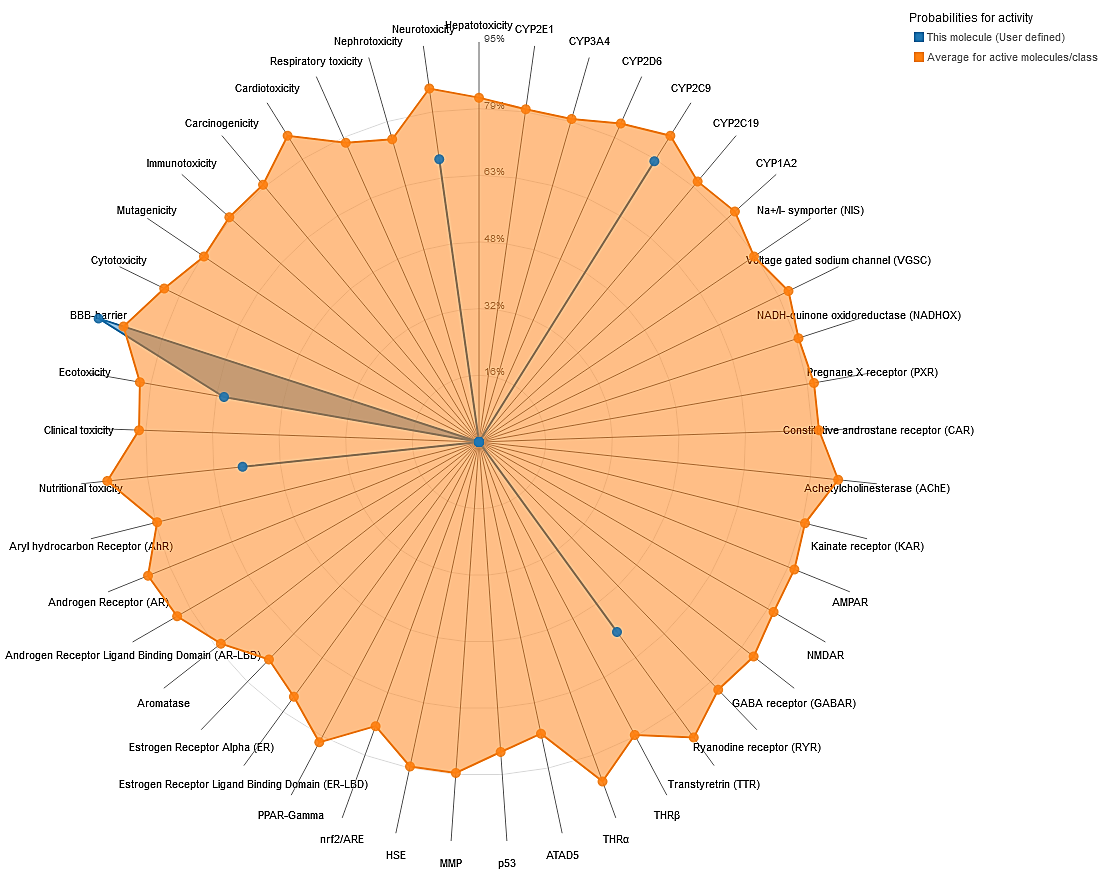
**

**Supplementary Figure S16:** The predicted biocompatibility/toxicity of titanium tetracarbide compund, PubChem CID: 139333, Molecular Weight: 97.93 g/mol (Computed by PubChem 2.2 (PubChem release 2025.04.14)), Molecular Formula: C_4_H_2_Ti, SMILES: C#[C-].C#[C-].[Ti+2], <https://pubchem.ncbi.nlm.nih.gov/compound/139333>. The toxicity radar chart (screen) is intended to illustrate the confidence of positive toxicity predictions with different bio-systems (quick results) of this chemical composition compared to the average of its class. Priyanka Banerjee, Emanuel Kemmler, Mathias Dunkel, Robert Preissner, ProTox 3.0: a webserver for the prediction of toxicity of chemicals, *Nucleic Acids Research*, Volume 52, Issue W1, 5 J 2024, Pages W513–W520 <https://tox.charite.de/>.<https://doi.org/10.1093/nar/gkae303> (***predicted LD50: 25 mg kg^−1^ and toxicity class: 2***).


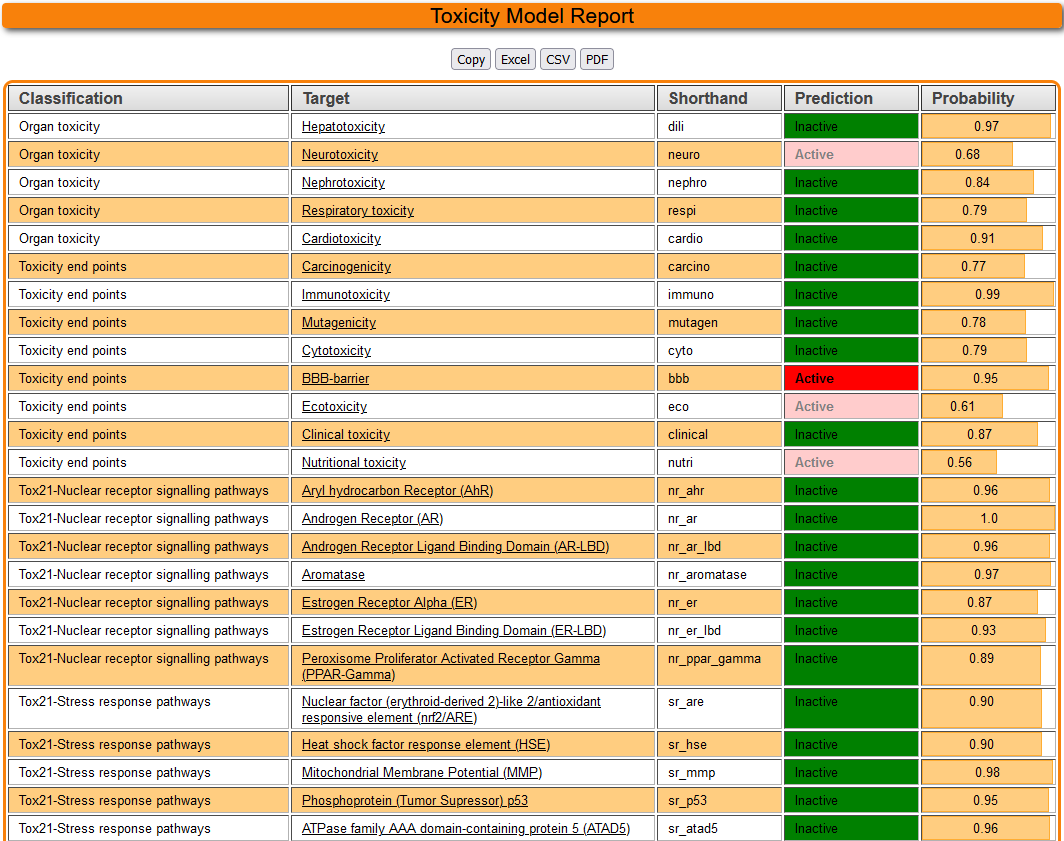


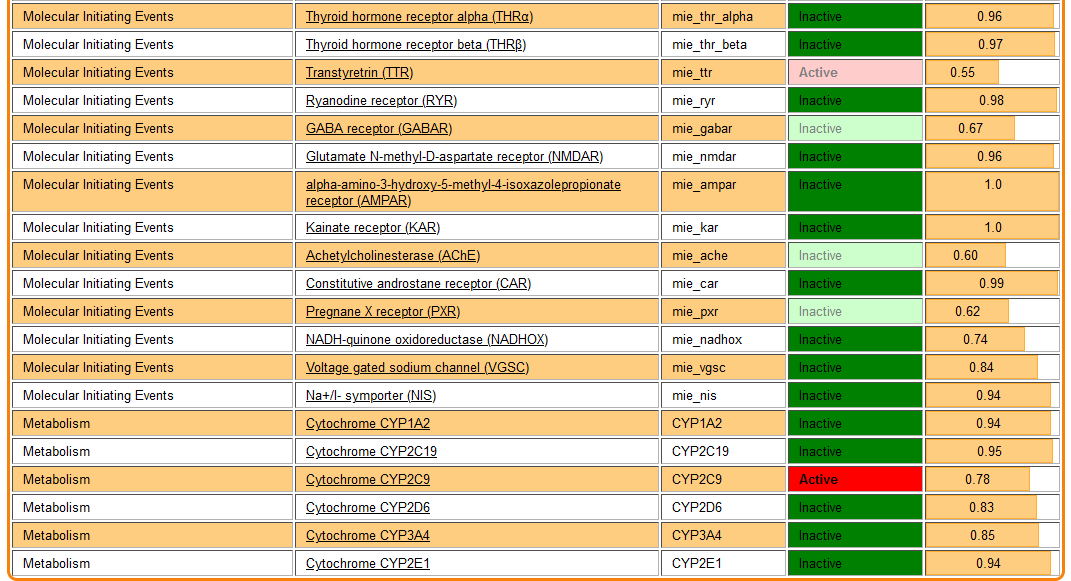


**Supplementary Figure S17:** The toxicity evaluations (screen sh.) of titanium tetracarbide to illustrate its predicted biocompatibility/toxicity with different biological systems and probability numbers. Priyanka Banerjee, Kemmler et al, ProTox 3.0: a webserver for the prediction of toxicity of chemicals, *Nucleic Acids Research*, Volume 52, I W1, 2024, P W513–W520 <https://tox.charite.de/>.<https://doi.org/10.1093/nar/gkae303>


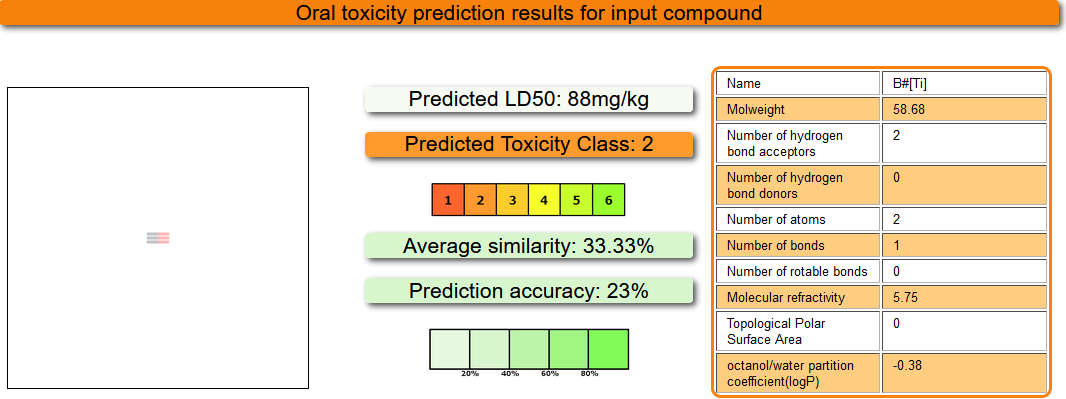


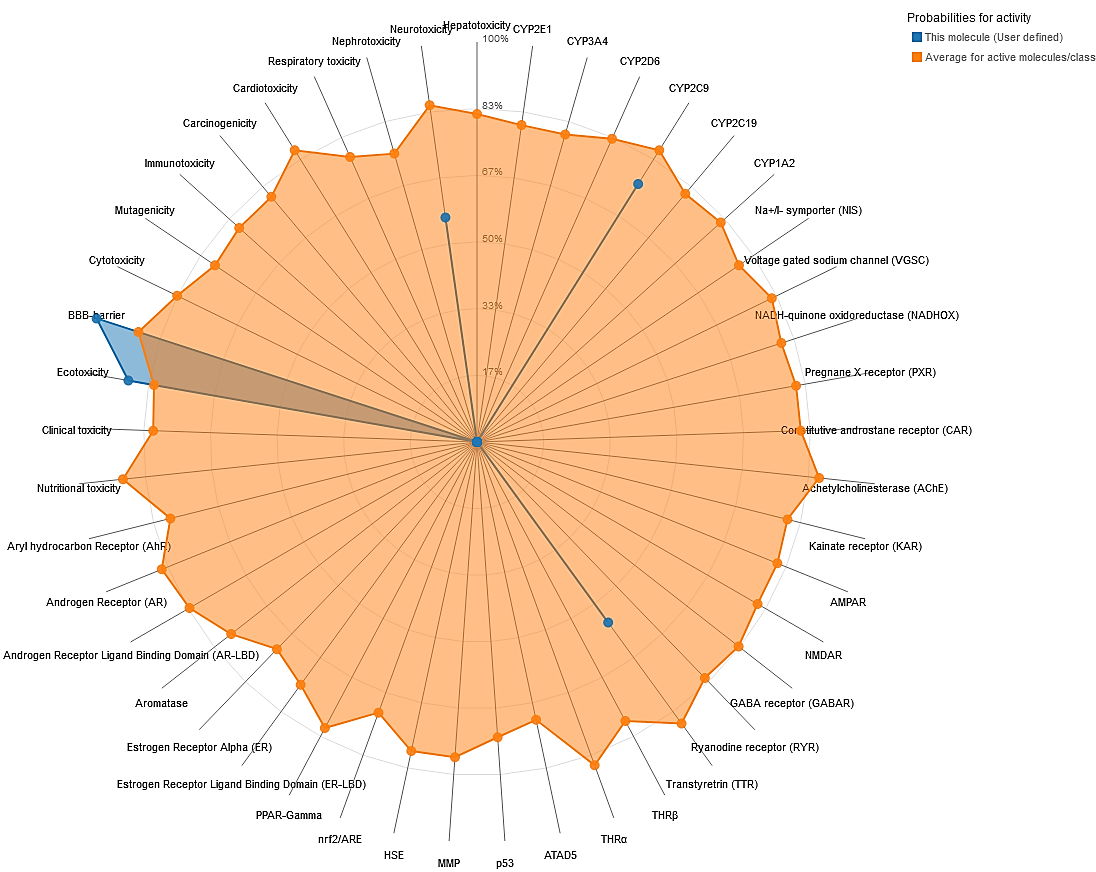


**Supplementary Figure S18:** The predicted biocompatibility/toxicity of titanium monoboride, PubChem CID: 13710852**,** Molecular Weight: 58.68 g/mol (Computed by PubChem 2.2 (PubChem release 2025.04.14)), Molecular Formula: BTi**,** SMILES: B#[Ti], <https://pubchem.ncbi.nlm.nih.gov/compound/13710852>. The toxicity radar chart (screen) is intended to illustrate the confidence of positive toxicity predictions with different bio-systems (quick results) of this chemical composition compared to the average of its class. Priyanka Banerjee, Emanuel Kemmler, Mathias Dunkel, Robert Preissner, ProTox 3.0: a webserver for the prediction of toxicity of chemicals, *Nucleic Acids Research*, Vol. 52, Issue W1, 5 J 2024, Pages W513–W520 <https://tox.charite.de/>.<https://doi.org/10.1093/nar/gkae303> (***predicted LD50: 88 mg kg^−1^ and toxicity class: 2***).


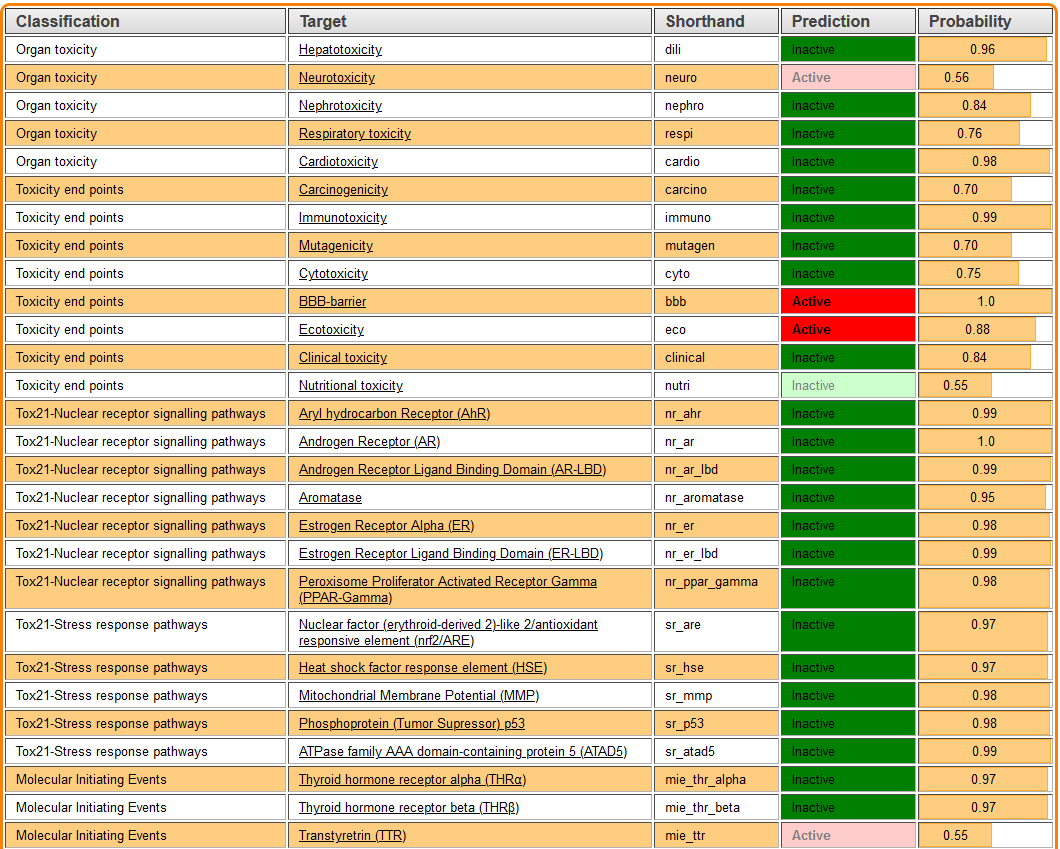


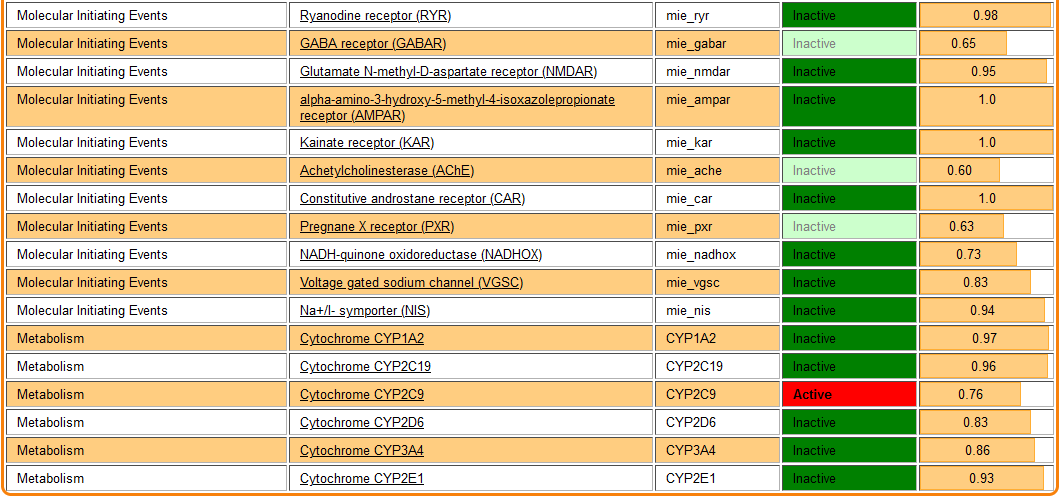


**Supplementary Figure S19:** The toxicity evaluations (screen) of titanium monoboride to illustrate its predicted biocompatibility/toxicity with different biological systems and probability numbers. Priyanka Banerjee, Kemmler et al, ProTox 3.0: a webserver for the prediction of toxicity of chemicals, *Nucleic Acids Research*, Volume 52, I W1, 2024, P W513–W520 <https://tox.charite.de/>.<https://doi.org/10.1093/nar/gkae303>


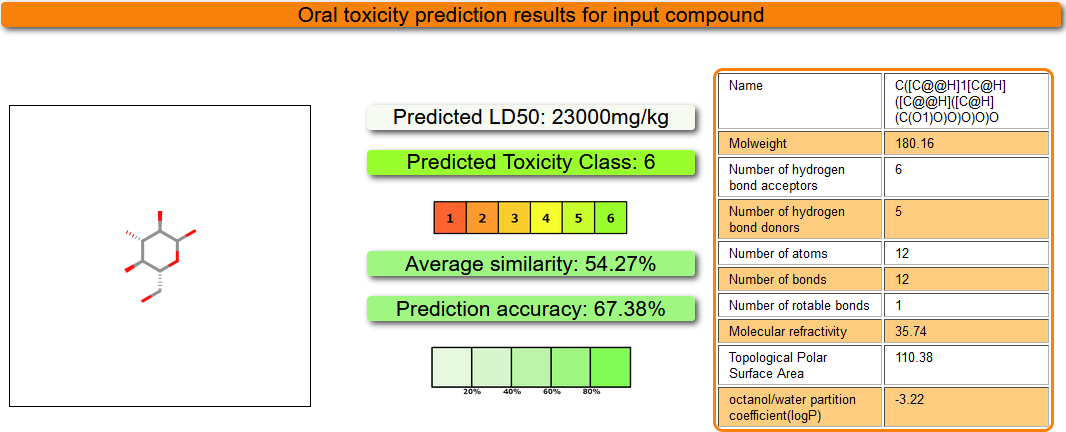


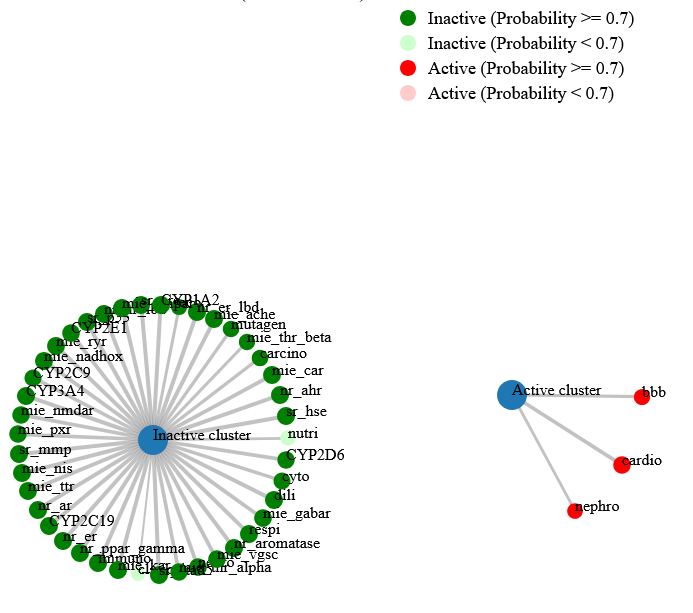


**Supplementary Figure S20:** The predicted biocompatibility/toxicity of D-Glucose, PubChem CID: 5988, MW, 180.16 g/mol (Computed by PubChem 2.2 (PubChem release 2025.04.14), FormulaC_6_H_12_O_6_, SMILES: C([C@@H]1[C@H]([C@@H]([C@H](C(O1)O)O)O)O)O, <https://pubchem.ncbi.nlm.nih.gov/compound/5793>. The toxicity network plot (screen sh.) is intended to illustrate the confidence of positive toxicity predictions with different bio-systems (quick results) of this chemical composition compared to the average of its class. P Banerjee, E Kemmler, M Dunkel, R Preissner, ProTox 3.0: *Nucleic Acids Research*, 2024, <https://tox.charite.de/>.<https://doi.org/10.1093/nar/gkae303> (***predicted LD50: 23000 mg kg^−1^, toxicity class: 6***).


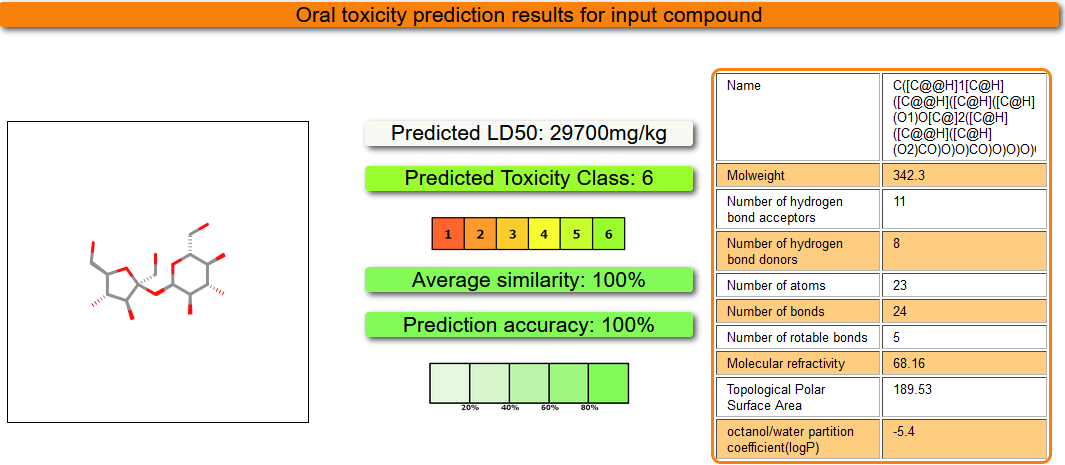


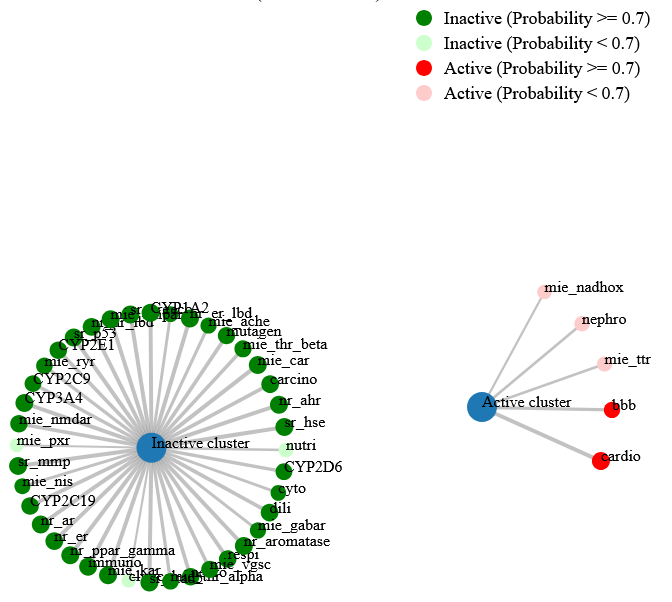


**Supplementary Figure S21:** The predicted biocompatibility/toxicity of Sucrose, PubChem CID: 5988, MW: 342.30 g/mol (computed by PubChem 2.2 (PubChem 2025.04.14)),: C12H22O11, SMILES:C([C@@H]1[C@H]([C@@H]([C@H]([C@H](O1)O[C@]2([C@H]([C@@H]([C@H](O2)CO)O)O)CO)O)O)O)O, <https://pubchem.ncbi.nlm.nih.gov/compound/5988>. The toxicity network plot (screen sh) is intended to illustrate the confidence of positive toxicity predictions with different bio-systems (quick results) of this chemical composition compared to the average of its class. P Banerjee, E Kemmler, M Dunkel, R Preissner, ProTox 3.0: *Nucleic Acids Research*, 2024, <https://tox.charite.de/>.<https://doi.org/10.1093/nar/gkae303> (***predicted LD50: 29700 mg kg^−1^, toxicity class: 6***).


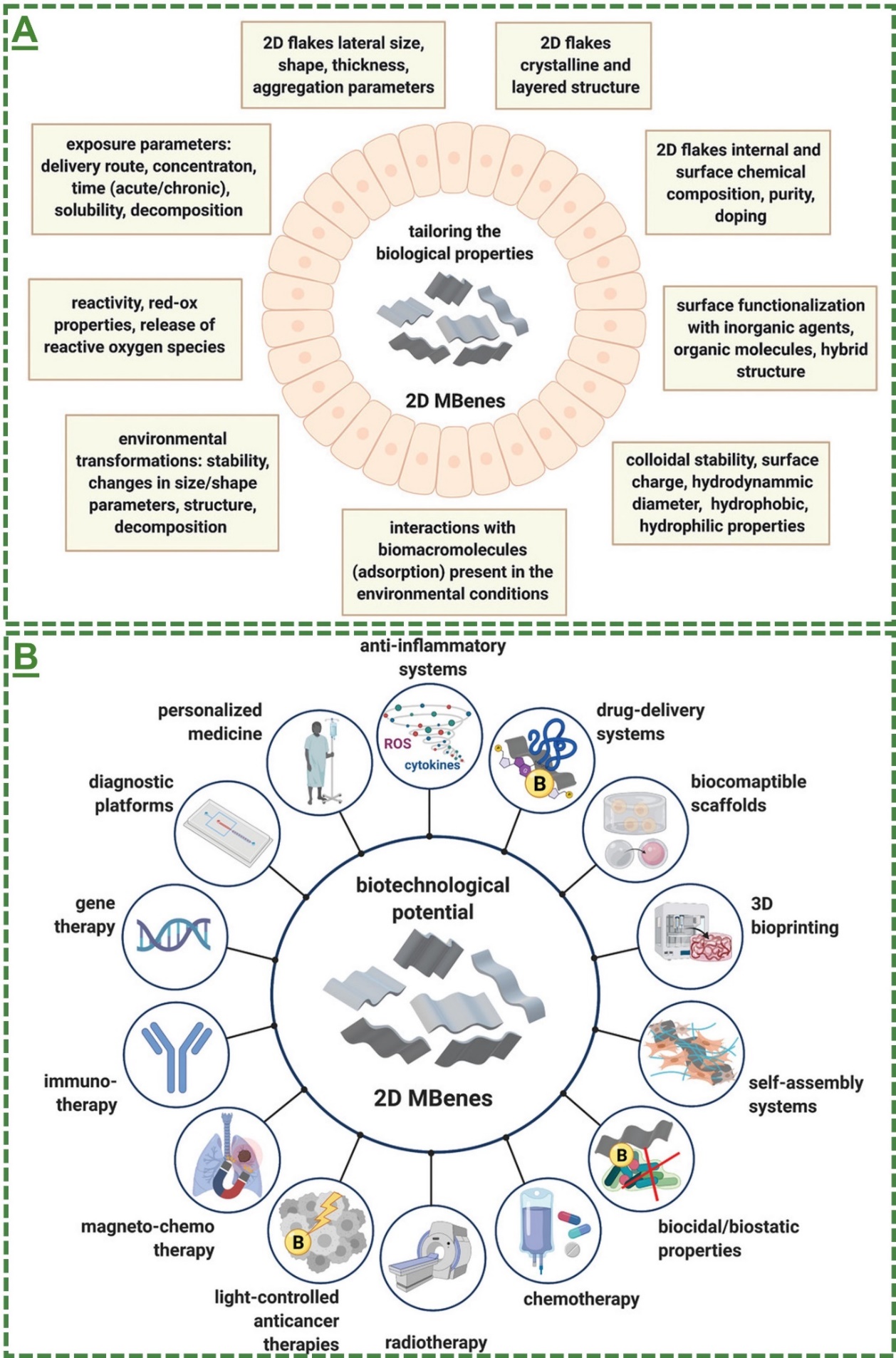


**Supplementary Figure S22:** The represented schematics depict the proposed features and expected biological impacts of 2D MBenes on living organisms and their envisioned future biotechnological applications (BioRender.com). This information is adapted with permission from Figures 5 and 6 of Jakubczak et al. (2021), *J. AFM* *Copyright*, *Wiley*.^75^, <https://doi.org/10.1002/adfm.202103048>


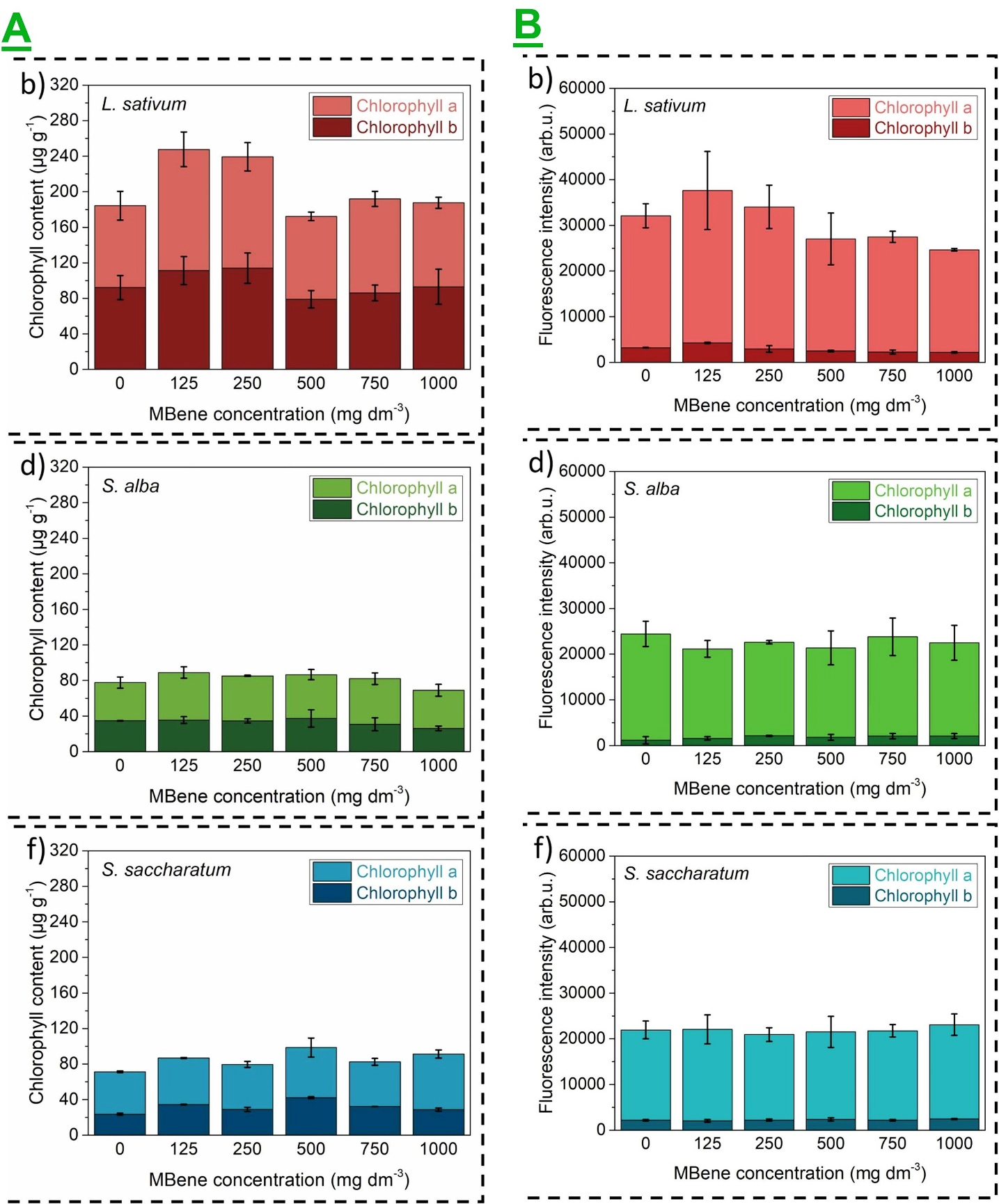


**Supplementary Figure S23:** The represented data depict the chlorophyll content from the extracts of *L. sativum*, *S. alba*, and *S. saccharatum* plant treated with MoAlB@MBene. It also shows the measured fluorescence intensity of chlorophyll a/b for the extracts collected from *these plants* incubated in the presence of different doses MoAlB@MBene. The data are adapted from Figures 5 and 6 (merged panels) Jakubczak et al. (2025), *JECE*, *Open Access,* *Elsevier* .^75^, <https://doi.org/10.1016/j.jece.2025.116971>


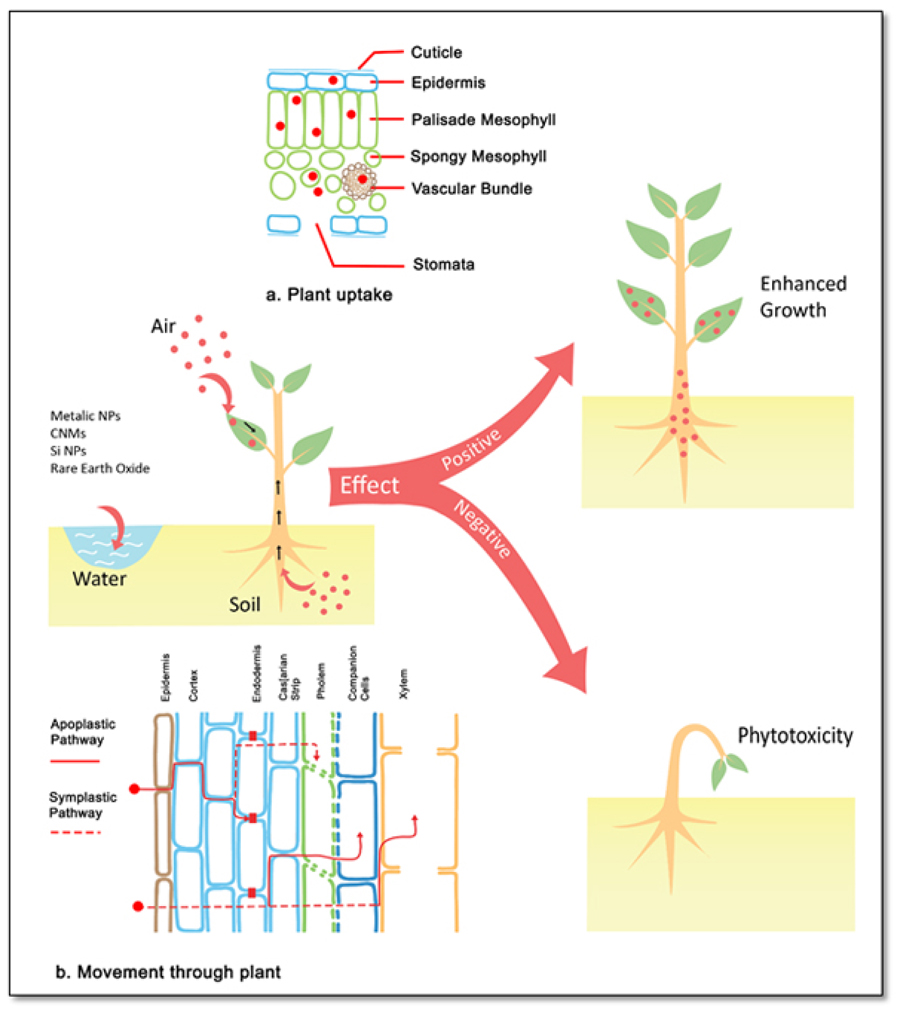


**Supplementary Figure S24:** The represented schematics depict the distribution, interactions, and the potential effects of nanomaterials on plants. This graphic is adapted from Table 1 of Madanayake1et al. (2021) with permission from *"Phytotoxicity of nanomaterials in agriculture." The Open Biotechnology Journal 15, no. 1 (2021), OA Copyright*. https://doi.org/10.2174/1874070702115010109


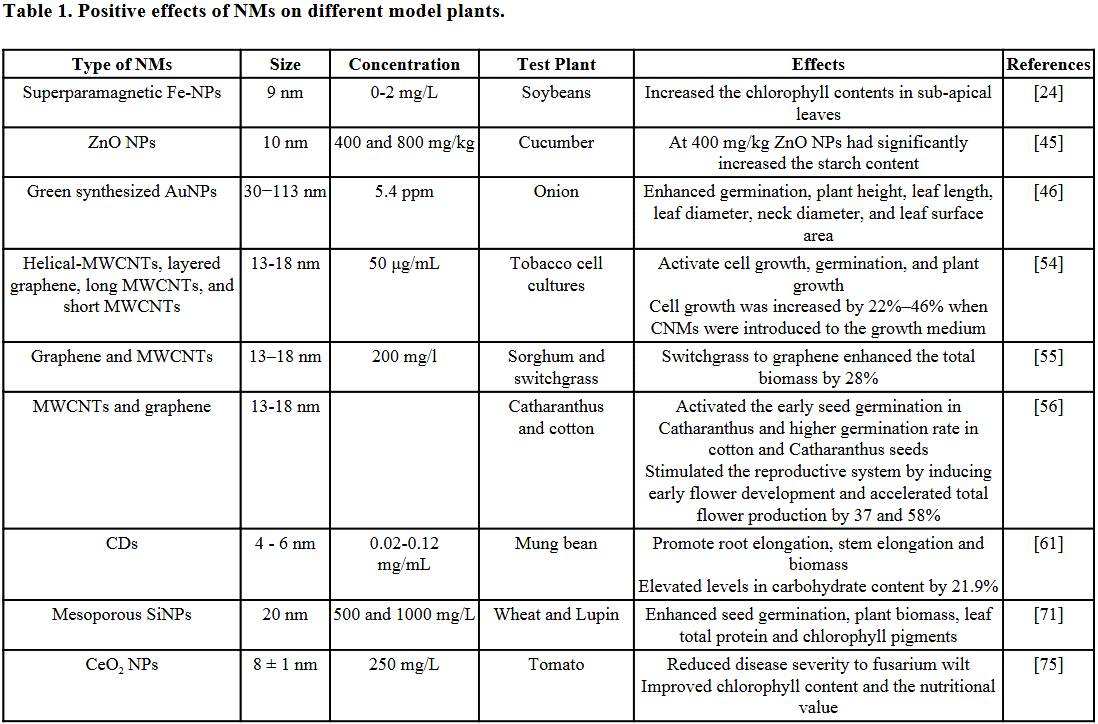


**Supplementary Figure S25:** The adapted summary of positive effects of different nanomaterials on different plant-based models. This information is adapted from Table 1 of Madanayake1et al. (2021) with permission from the reference*, "Phytotoxicity of nanomaterials in agriculture." The Open Biotechnology Journal 15, no. 1 (2021), O.A., J. Copyright*. https://doi.org/10.2174/1874070702115010109


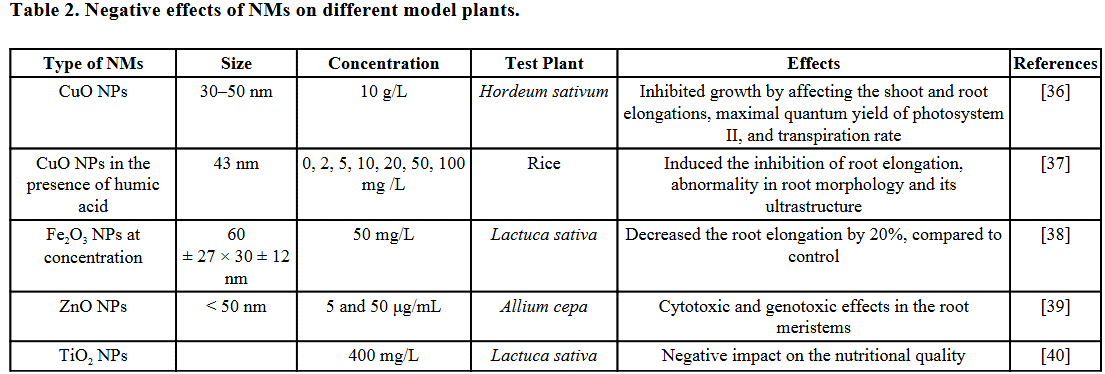


**Supplementary Figure S26:** The adapted summary of negative effects of different nanomaterials on different plant-based models. This information is adapted from Table 2: Madanayake1et al. (2021) with permission from the reference*, "Phytotoxicity of nanomaterials in agriculture." The Open Biotechnology Journal 15, no. 1 (2021), O.A., J Copyright*. https://doi.org/10.2174/1874070702115010109


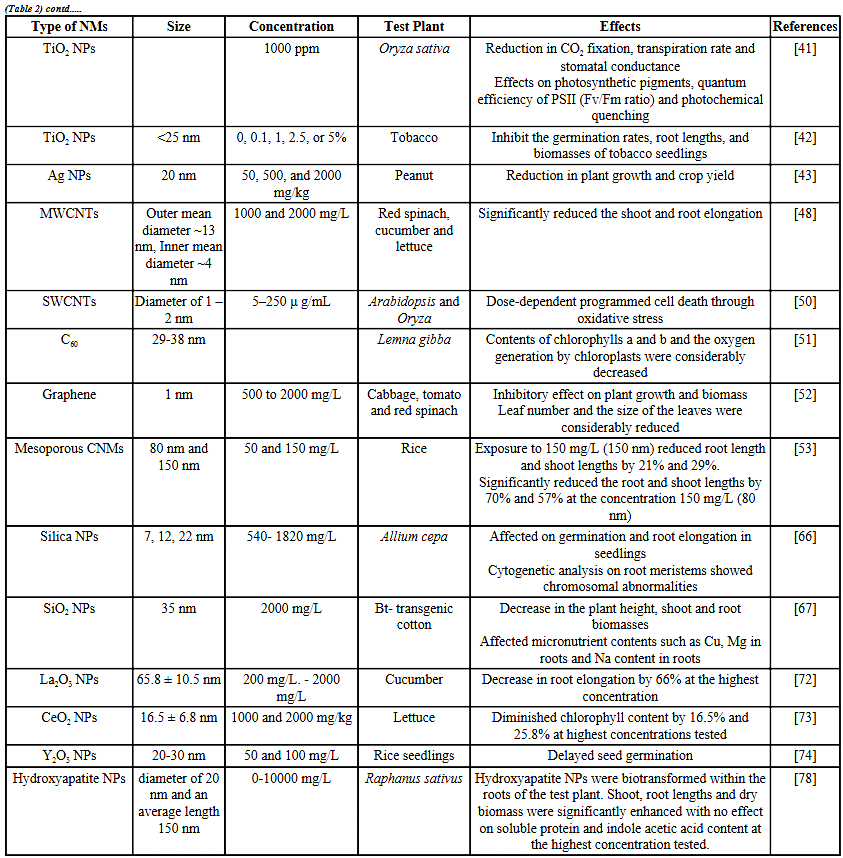


**Supplementary Figure S27:** The adapted summary of negative effects of different nanomaterials on different plant-based models. This information is adapted from Table 2: Madanayake1et al. (2021) with permission from the reference*, "Phytotoxicity of nanomaterials in agriculture." The Open Biotechnology Journal 15, no. 1 (2021), O.A., J Copyright*. https://doi.org/10.2174/1874070702115010109


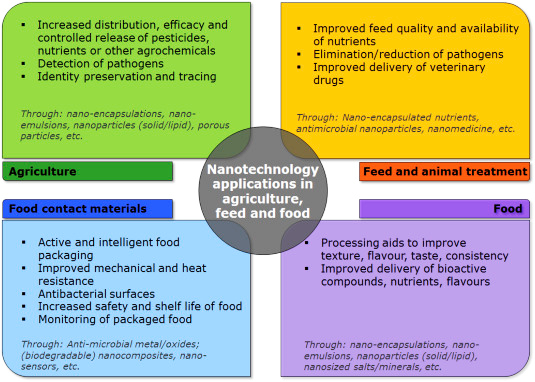


**Supplementary Figure S28:** The adapted schematic depicts the potential applications of nanotechnology and nanomaterials in agriculture, feed, and food sectors. This viewpoint is adapted from Figure 1 of Amenta et al. (2021) with permission from the reference*, "Regulatory aspects of nanotechnology in the agri/feed/food sector in EU and non-EU countries." Regulatory Toxicology and Pharmacology 73, no. 1 (2015): 463-476.Journal Copyright*. http://dx.doi.org/10.1016/j.yrtph.2015.06.016


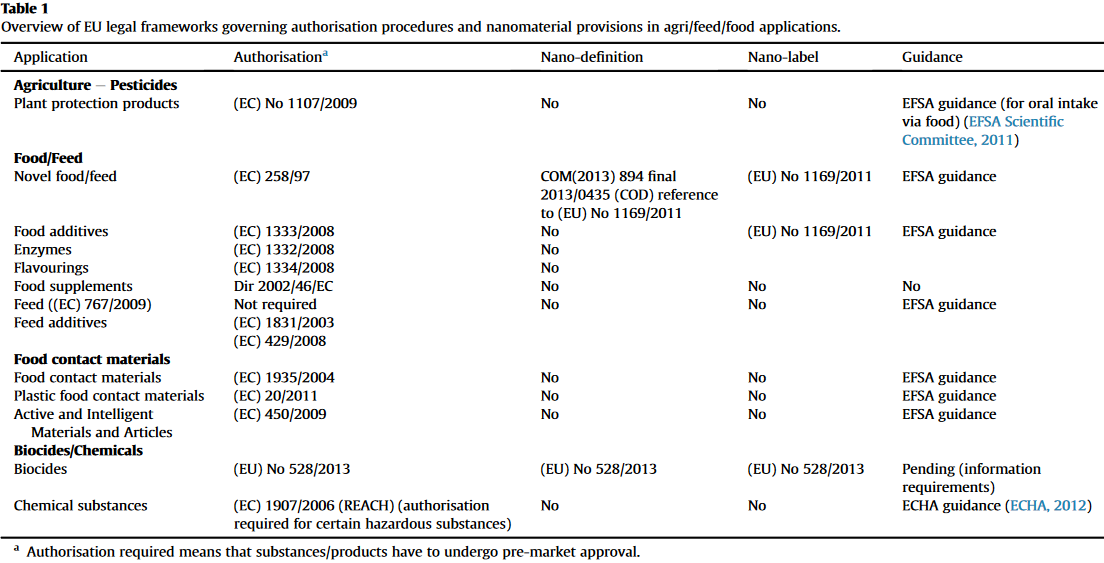


**Supplementary Figure S29:** The adapted summery depicts an overview of European legal frameworks governing authorization procedures and the proposed nanomaterial provisions for agri/feed/food applications. This tabulation data was adapted from Table 1 of Amenta et al. (2021) with permission from the reference*, "Regulatory aspects of nanotechnology in the agri/feed/food sector in EU and non-EU countries." Regulatory Toxicology and Pharmacology 73, no. 1 (2015): 463-476.Journal Copyright*. http://dx.doi.org/10.1016/j.yrtph.2015.06.016


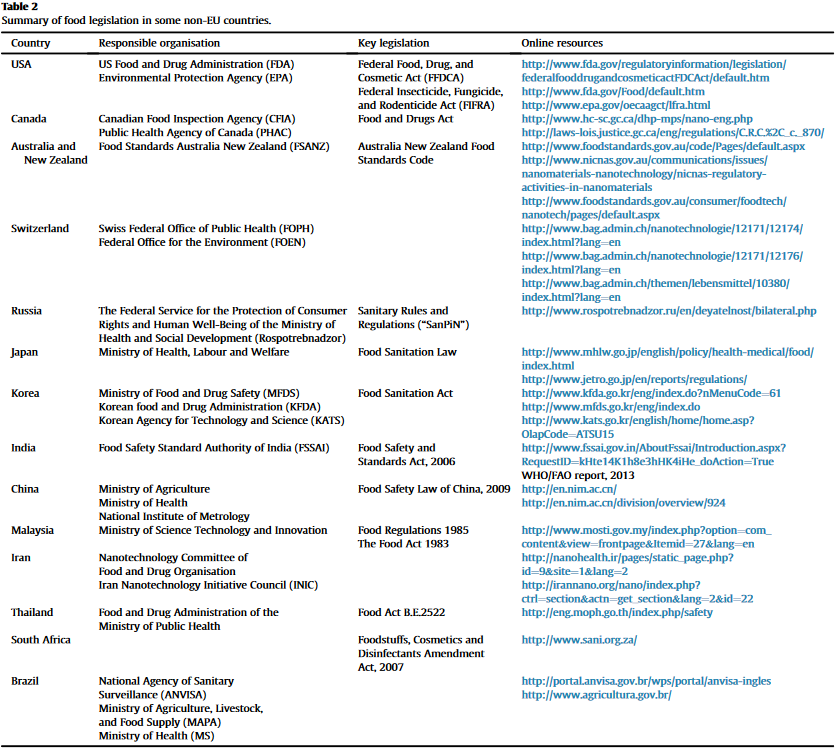


**Supplementary Figure S30:** The adapted table depicts a summary of the food legislation in some non-European countries. It was adapted from Table 2 of Amenta et al. (2021) with permission from the reference*, "Regulatory aspects of nanotechnology in the agri/feed/food sector in EU and non-EU countries." Regulatory Toxicology and Pharmacology 73, no. 1 (2015): 463-476.Journal Copyright*. http://dx.doi.org/10.1016/j.yrtph.2015.06.016


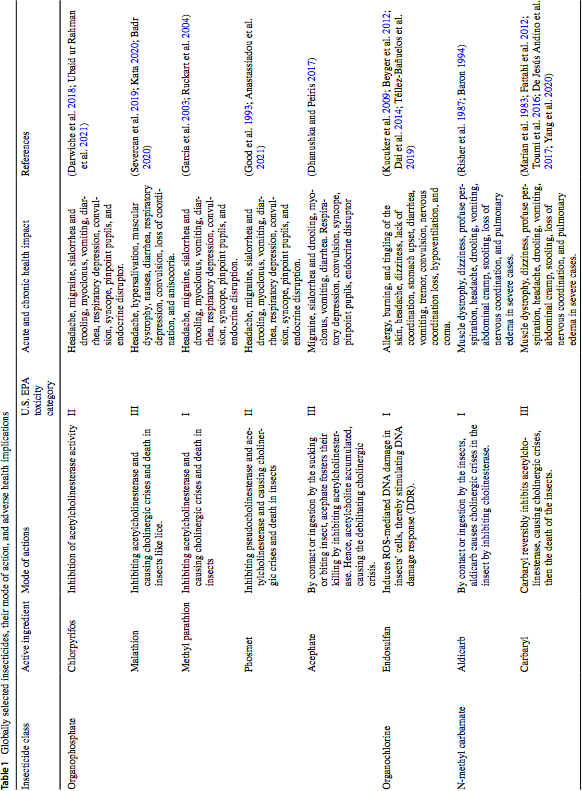


**Supplementary Figure S31:** The table depicts a summary of the globally selected insecticides: their mode-of-action and proposed adverse health implications. It was adapted from Table 1 of Okagu et al. (2023) with permission from *"Overhauling the ecotoxicological impact of synthetic pesticides using plants’ natural products: a focus on Zanthoxylum metabolites." Environmental Science and Pollution Research 30, no. 26 (2023): 67997-68021.* <https://doi.org/10.1007/s11356-023-27258-w>


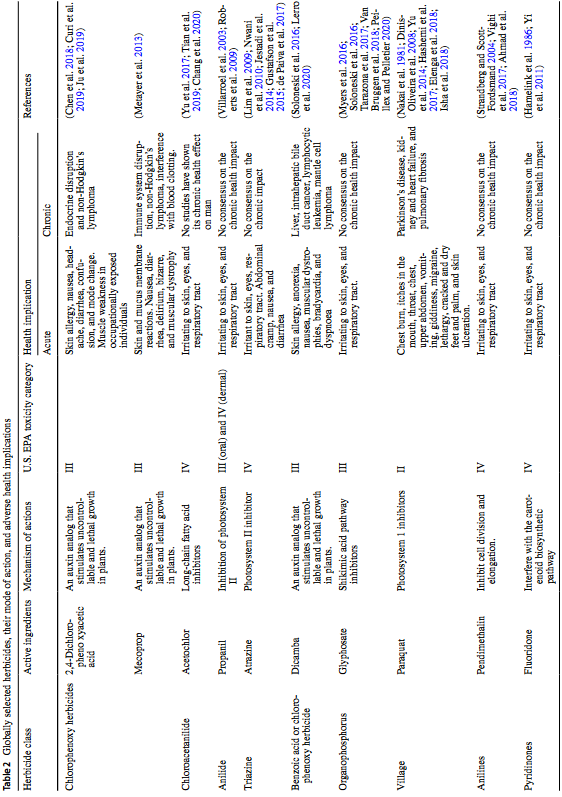


**Supplementary Figure S32:** The table depicts a summary of the globally selected herbicides: their mode-of-action and proposed adverse health implications. It was adapted from Table 1 of Okagu et al. (2023) with permission from *"Overhauling the ecotoxicological impact of synthetic pesticides using plants’ natural products: a focus on Zanthoxylum metabolites." Environmental Science and Pollution Research 30, no. 26 (2023): 67997-68021.* <https://doi.org/10.1007/s11356-023-27258-w>
